# Balancing Efficiency and Equity in Population-Wide CKD Screening

**DOI:** 10.1001/jamanetworkopen.2025.4740

**Published:** 2025-04-14

**Authors:** Marika M. Cusick, Rebecca L. Tisdale, Alyce S. Adams, Glenn M. Chertow, Douglas K. Owens, Joshua A. Salomon, Jeremy D. Goldhaber-Fiebert

**Affiliations:** 1 Department of Health Policy, School of Medicine, Stanford University, Stanford, California; 2 Veterans Affairs Palo Alto Health Care System, Palo Alto, California; 3 Division of Primary Care and Population Health, Department of Medicine, School of Medicine, Stanford University, Stanford, California; 4 Department of Epidemiology and Population Health, School of Medicine, Stanford University, Stanford, California; 5 Division of Nephrology, Department of Medicine, School of Medicine, Stanford University, Stanford, California

## Abstract

**Importance:**

In the era of sodium-glucose cotransporter 2 (SGLT2) inhibitors, population-wide screening for chronic kidney disease (CKD) may provide good value, yet implications across racial and ethnic groups are unknown.

**Objective:**

To evaluate the health outcomes, costs, and cost-effectiveness of population-wide CKD screening for 4 racial and ethnic groups.

**Design, Setting, and Participants:**

In this cost-effectiveness analysis, a decision-analytic Markov model was separately calibrated to simulate CKD progression among simulated cohorts of US Hispanic adults, non-Hispanic Black adults, non-Hispanic White adults, and adults who belong to additional racial and ethnic groups (ie, Asian and multiracial individuals and those self-reporting other race and ethnicity). Effectiveness of SGLT2 inhibitors was derived from the Dapagliflozin and Prevention of Adverse Outcomes in Chronic Kidney Disease trial. Mortality, quality-of-life weights, and cost estimates were obtained from published cohort studies, randomized clinical trials, and Centers for Medicare & Medicaid Services data. Analyses were conducted from January 1, 2023, to November 6, 2024.

**Exposures:**

One-time or periodic (every 10 or 5 years) screening for albuminuria, initiated between age 35 and 75 years, with and without addition of SGLT2 inhibitors to angiotensin-converting enzyme inhibitor or angiotensin-receptor blocker therapy for CKD.

**Main Outcomes and Measures:**

Lifetime cumulative incidence of kidney failure requiring kidney replacement therapy (KRT); discounted life-years (LYs), quality-adjusted LYs (QALYs), lifetime health care costs (in 2024 US dollars), and incremental cost-effectiveness ratios.

**Results:**

Under the status quo, non-Hispanic Black adults aged 35 years had the highest lifetime incidence of kidney failure requiring KRT (6.2% [95% UI, 2.8%-10.6%]) compared with Hispanic adults (3.6% [95% UI, 1.1%-6.7%]), non-Hispanic White adults (2.3% [95% UI, 0.4%-5.2%]), and adults from additional racial and ethnic groups (3.3% [95% UI, 1.2%-6.5%]). Screening every 5 years from ages 55 to 75 years combined with SGLT2 inhibitors reduced incidence of KRT and increased LYs across all racial and ethnic groups, with the largest average changes observed for non-Hispanic Black adults (0.8–percentage point decrease and 0.19-year increase). Every 5-year screening from age 55 to 75 years cost $99 100/QALY gained for the overall population and less than $150 000/QALY gained across racial and ethnic groups, with the lowest cost observed for non-Hispanic Black adults ($73 400/QALY gained). Screening starting at age 35 years was only cost-effective for non-Hispanic Black adults ($115 000/QALY gained).

**Conclusions and Relevance:**

In this cost-effectiveness analysis, population-wide screening for CKD from ages 55 to 75 years was projected to improve population health, was cost-effective, and reduced disparities across 4 racial and ethnic groups. Starting population-wide screening at younger ages was projected to further benefit non-Hispanic Black adults.

## Key Points

**Question:**

What are the health outcomes, costs, and cost-effectiveness of chronic kidney disease (CKD) screening across racial and ethnic groups?

**Findings:**

In this cost-effectiveness analysis, population-wide screening for CKD combined with sodium-glucose cotransporter 2 inhibitors yielded larger reductions in kidney failure incidence and gains in life expectancy among non-Hispanic Black adults compared with Hispanic adults, non-Hispanic White adults, and adults from additional racial and ethnic groups (ie, Asian and multiracial individuals and those self-reporting other race and ethnicity). While initiating screening at age 55 years was the preferred strategy under common cost-effectiveness benchmarks for the overall population, screening at younger ages further narrowed disparities.

**Meaning:**

These findings suggest that policymakers should consider trade-offs between efficiency and equity when evaluating population-wide CKD screen-and-treat interventions.

## Teaser

This cost-effectiveness analysis examines the health outcomes, costs, and cost-effectiveness of different population-wide chronic kidney disease (CKD) screening and treatment scenarios among US Black adults, Hispanic adults, White adults, and adults from additional racial and ethnic groups.

## Introduction

Chronic kidney disease (CKD) is a common and costly cause of morbidity and mortality in the United States, affecting up to 1 in 7 adults.^1^ The burden of CKD disproportionately affects persons in racial and ethnic minority groups.^2,3^ Lifetime risk of progression to kidney failure requiring kidney replacement therapy (KRT), dialysis or kidney transplantation, is more than 3-fold higher for non-Hispanic Black adults and 1.5-fold higher for Hispanic adults compared with non-Hispanic White adults.^4,5^ CKD disparities are driven by factors within and outside of the health care system, including suboptimal management of mild-to-moderate CKD, lower rates of health insurance coverage and access to routine medical care, and social determinants (eg, racial discrimination, structural inequality).^6,7,8,9,10,11,12,13^ Interventions to reduce disparities in kidney failure across racial and ethnic groups are needed.

Early detection and management of CKD through screening may help to reduce CKD disparities. In 2012, the US Preventive Services Task Force found insufficient evidence to recommend screening due to the lack of effective treatment options.^14^ However, the introduction of sodium-glucose cotransporter 2 (SGLT2) inhibitors, a practice-changing therapy for CKD, could alter the value of CKD screening.^15,16^ In previously published analyses, we demonstrated that population-wide screening for CKD in the era of SGLT2 inhibitor use increased life expectancy, reduced the burden of kidney failure, and was cost-effective.^17,18^ However, our previous analyses did not evaluate whether population-wide screening for CKD could yield differential effectiveness and reduce disparities across racial and ethnic groups. In this study, we evaluate the health outcomes, disparity reductions, costs, and cost-effectiveness associated with population-wide screening for CKD across racial and ethnic groups.

## Methods

We evaluated population-wide CKD screen-and-treat interventions in simulated cohorts representing all US adults currently aged 35, 45, 55, 65, and 75 years across 4 racial and ethnic groups: Hispanic, non-Hispanic Black, non-Hispanic White, and additional racial and ethnic groups (ie, Asian and multiracial individuals and those self-reporting other race and ethnicity). For each group, we separately calibrated a decision-analytic Markov model to replicate racial and ethnic group–specific CKD prevalence and progression and computed the clinical outcomes, costs, and cost-effectiveness of interventions that vary by initiation age, screening frequency, and treatment regimen. We assessed changes in health outcomes across racial and ethnic groups to quantify reductions in CKD disparities. We aggregated race and ethnicity–specific results to assess clinical outcomes, costs, and cost-effectiveness across the overall population.

This study was exempt from institutional review and the requirement for informed consent because all data were publicly available and did not involve human participants. This study followed the Consolidated Health Economic Evaluation Reporting Standards (CHEERS) reporting guidelines.^19^

### Populations Studied

Racial and ethnic groups were defined for compatibility with National Health and Nutrition Examination Survey (NHANES) 2013-2018 self-reported race and ethnicity categories.^20^ Hispanic includes individuals self-reporting as either Mexican American or other Hispanic. Additional racial and ethnic groups includes those self-identifying as non-Hispanic Asian or other race (including multiracial).

### Clinical Interventions

We assessed screen-and-treat interventions initiated and stopped between ages 35 and 75 years at 10-year increments. We considered one-time and periodic (every 10 or 5 years) screening through age 75 years. Screening for CKD included testing for albuminuria (≥30 mg/g) using the urine-albumin creatinine ratio (UACR) test and subsequent serum creatinine testing to calculate estimated glomerular filtration rate (eGFR) by individual sex and age.^21^ Patients with albuminuria and eGFR of less than 60 mL/min/1.73 m^2^ underwent retroperitoneal ultrasound to detect structural kidney abnormalities.

Patients with detected CKD may receive treatment. We considered screen-and-treat strategies with and without the addition of SGLT2 inhibitors to conventional CKD therapy of angiotensin-converting enzyme (ACE) inhibitors or angiotensin-receptor blockers (ARBs). Patients receiving conventional CKD therapy benefited from slowed CKD progression.^22,23,24,25^ Benefits of combined therapy of SGLT2 inhibitors and conventional CKD therapy included slowed CKD progression and reductions in all-cause mortality, as derived from the Dapagliflozin and Prevention of Adverse Outcomes in Chronic Kidney Disease (DAPA-CKD) clinical trial.^15^ Patients were eligible for SGLT2 inhibitors if they had albuminuria and eGFR of less than 60 mL/min/1.73 m^2^. Given possible heterogeneity in effectiveness of the combined therapy by diabetes status, we computed age-specific SGLT2 inhibitor effectiveness as the average of effectiveness in groups with and without diabetes, weighted by diabetes prevalence in the treatment-eligible population from each racial and ethnic group.

Patients with false-positive albuminuria screening results on treatment did not receive any clinical benefits yet incurred treatment costs. Treated patients could experience rare adverse events, including angioedema, euglycemic diabetic ketoacidosis, and genital yeast or fungal infections.^26,27^ Treatment discontinuation rates were derived from the DAPA-CKD trial.^15^ We assumed equal rates of adverse events and treatment discontinuation by racial and ethnic group.

### Natural History Model

We used our previously published state-transition Markov cohort model to simulate the natural history of CKD in the 4 racial and ethnic groups.^17,18^ Patients transitioned at 3-month intervals between model stages defined by eGFR, UACR, and CKD detection and treatment status.

We classified eGFR in stages: greater than 90 mL/min/1.73 m^2^ (stage G1), 60 to 89 mL/min/1.73 m^2^ (stage G2), 45 to 59 mL/min/1.73 m^2^ (stage G3a), 30 to 44 mL/min/1.73 m^2^ (stage G3b), 15 to 29 mL/min/1.73 m^2^ (stage G4), 12 to 14 mL/min/1.73 m^2^ (kidney failure not requiring KRT), and less than 12 mL/min/1.73 m^2^ (kidney failure requiring KRT).^17,28,29^ We classified albuminuria stages using UACR: less than 30 mg/g (no albuminuria, A1), 30 to 299 mg/g (microalbuminuria, A2), and greater than 300 mg/g (macroalbuminuria, A3). We assumed patients progressed only 1 eGFR and/or albuminuria stage within a 3-month interval and could not progress to a less severe CKD stage.^28,29^ Once CKD was detected, patients remained detected for their remaining lifetime and were eligible for treatment.

We used a bayesian approach (sampling importance resampling) to calibrate our model to racial and ethnic group–specific estimates of CKD prevalence, detection, and treatment status from NHANES.^20^ Further details on NHANES estimation, calibration methods, and model fit are provided in the eMethods and eTable 1 in Supplement 1.

### Mortality, Costs, and Quality of Life

Mortality, costs, and health-related quality-of-life weights differ across CKD stages according to eGFR. We obtained baseline mortality rates from 2019 US age-, sex-, and racial and ethnic group–specific life tables (eMethods in Supplement 1). Mortality rates increased for patients in stage G2 and above according to eGFR stage–specific mortality hazard ratios.^30^ We assumed patients with kidney failure not requiring KRT had the same mortality hazards as patients in stage G4.

We obtained age- and sex-specific baseline health care expenditures from the Medical Expenditure Panel Survey.^31^ While patients in stage G1 and G2 incurred the same costs as the general population, patients in more advanced eGFR stages incurred additional CKD stage–specific costs (eMethods and eTable 2 in Supplement 1).^32^ We used annual per-person cost estimates from the US Renal Data System for persons with kidney failure requiring KRT.^33^

Screening costs included costs for conducting UACR, serum creatinine, and retroperitoneal ultrasonography tests. We did not include clinician costs under the assumption that screening would take place at a routine primary care visit. Treated patients incurred costs for conventional CKD therapy and/or SGLT2 inhibitors according to Centers for Medicare & Medicaid Services negotiated prices.^34,35,36^ Patients with treatment-related adverse events incurred reaction-specific costs.^37,38,39^

We assumed health-related quality-of-life weights declined with more advanced eGFR stages according to published literature.^40,41^ Patients experiencing adverse events from treatment incurred 3-month health-related quality-of-life decrements.^42,43,44^

### Statistical Analysis

#### Base Case Analysis

Table 1 reports model parameter values and ranges explored in sensitivity analyses.^2,15,17,20,22,23,24,25,27,30,32,35,36,39,40,42,43,44,45,46,47,48,49,50,51,52,53,54,55,56^ Our cost-effectiveness analysis was conducted from the health care sector perspective according to Second Panel on Cost-Effectiveness in Health and Medicine recommendations.^36^ Outcomes included lifetime cumulative incidence of kidney failure requiring KRT, averted cases of kidney failure requiring KRT (eMethods in Supplement 1), health care sector costs (in 2024 US dollars), life-years (LYs), quality-adjusted LYs (QALYs), and incremental cost-effectiveness ratios. We discounted LYs, QALYs, and costs at 3% annually. Costs were inflated to 2024 US dollars using Personal Health Care Expenditure deflators.^57,58^ We computed differences in incidence of kidney failure requiring KRT, LYs, and QALYs among racial and ethnic groups across scenarios to evaluate intervention effects on health disparities. We conducted probabilistic analyses to compute point estimates and 95% uncertainty intervals (UIs) for all outcomes (eMethods and eTable 3 in Supplement 1).^59^ We report main results for the 35-year-old cohort across our 4 racial and ethnic groups. Results for older age cohorts are provided in eTables 24 to 27 in Supplement 1. We aggregated results among all racial and ethnic groups to reflect the overall population using group-specific population proportions from the US Census (eMethods in Supplement 1). Analyses were conducted from January 1, 2023, to November 6, 2024.

**Table 1.** Base Case Model Parameters

Parameter	Value (95% UI)	Source
**Screening parameters**		
UACR screening sensitivity	0.87 (0.81-0.91)	Wu et al,^45^ 2014
UACR screening specificity	0.88 (0.84-0.91)	Wu et al,^45^ 2014
Cost of UACR screening, $	49 (37-62)	Sanders et al,^36^ 2016; and FindLabTest^46^
Probability of treatment initiation after diagnosis	0.75 (0.50-1.00)	Golan et al,^47^ 1999; and Boulware et al,^48^ 2003
**Diagnosis parameters**		
Cost of estimated GFR, $	23 (17-29)	Sanders et al,^36^ 2016; and FindLabTest^49^
Cost of retroperitoneal ultrasound, $	420 (312-526)	New Choice Health^50^
**Treatment parameters**		
ACE inhibitor and ARB therapy, CKD progression reduction, hazard ratio	0.81 (0.52-1.00)	Ruggenenti et al,^22^ 1999; Brenner et al,^23^ 2001; Hou et al,^24^ 2006; and Hou et al,^25^ 2007
Monthly cost of ACE inhibitor and ARB therapy, $	34 (25-43)	Nichols et al,^32^ 2020; and Sanders et al,^36^ 2016
SGLT2 inhibitor, CKD progression reduction among individuals without diabetes, hazard ratio	0.51 (0.34-0.72)	Heerspink et al,^15^ 2020; and Goldhaber-Fiebert and Jalal,^51^ 2016
SGLT2 inhibitor, all-cause mortality reduction among individuals without diabetes, hazard ratio	0.54 (0.32-0.86)	Heerspink et al,^15^ 2020; and Goldhaber-Fiebert and Jalal,^51^ 2016
SGLT2 inhibitor, CKD progression reduction among individuals with diabetes, hazard ratio	0.57 (0.45-0.70)	Heerspink et al,^15^ 2020; and Goldhaber-Fiebert and Jalal,^51^ 2016
SGLT2 inhibitor, all-cause mortality reduction among individuals with diabetes, hazard ratio	0.75 (0.56-0.98)	Heerspink et al,^15^ 2020; and Goldhaber-Fiebert and Jalal,^51^ 2016
Annual discontinuation rate for SGLT2 inhibitors	0.051 (0.027-0.083)	Heerspink et al,^15^ 2020
Monthly cost of SGLT2 inhibitors, $	180 (134-234)	Centers for Medicare & Medicaid Services^35^; and Sanders et al,^36^ 2016
Disutility associated with medication-related angioedema adverse event	0.01 (0.0027-0.02)	Richman et al,^42^ 2016
Cost increase from angioedema medication-related adverse event, $	3876 (2887-4868)	Centers for Medicare & Medicaid Services^39^
Proportion of diagnosed persons who experience an angioedema medication-related serious adverse event, %	0.1 (0.01-1.0)	Vleeming et al,^52^ 1998
Disutility associated with genital infection adverse event	0.001 (0.0002-0.006)	Sullivan and Ghushchyan,^43^ 2016
Cost increase from genital infection adverse event, $	150 (111-189)	Neslusan et al,^53^ 2018
Annual rate of genital infection adverse event	0.037 (0.027-0.051)	Clar et al,^27^ 2012
Disutility associated with euglycemic diabetic ketoacidosis adverse event	0.0098 (0.005-0.016)	Peasgood et al,^44^ 2016
Cost increase from euglycemic diabetic ketoacidosis adverse event, $	30 597 (22 815-38 423)	Lyerla et al,^54^ 2021
Annual rate of euglycemic diabetic ketoacidosis adverse event	0.002 (0.0002-0.006)	Douros et al,^55^ 2020
**Age-specific diabetes prevalence (among those eligible for SGLT2 inhibitor treatment), %**		
Hispanic		
30-39 y	7.8 (0-33.3)	US CDC^20^
40-49 y	45.9 (18.2-72.7)	US CDC^20^
50-59 y	49.1 (27.3-68.2)	US CDC^20^
60-69 y	50.6 (38.4-61.6)	US CDC^20^
70-79 y	51.2 (41.7-60.0)	US CDC^20^
Non-Hispanic Black		
30-39 y	22.5 (0-50.0)	US CDC^20^
40-49 y	33.6 (15.4-53.8)	US CDC^20^
50-59 y	31.9 (21.5-43.1)	US CDC^20^
60-69 y	41.3 (32.5-50.9)	US CDC^20^
70-79 y	51.3 (41.9-61.0)	US CDC^20^
Non-Hispanic White		
30-39 y	6.4 (0-21.4)	US CDC^20^
40-49 y	25.9 (5.6-44.4)	US CDC^20^
50-59 y	50.9 (34.3-68.6)	US CDC^20^
60-69 y	42.9 (33.0-53.2)	US CDC^20^
70-79 y	34.2 (28.9-39.4)	US CDC^20^
Additional racial and ethnic groups^a^		
30-39 y	8.0 (0-40.0)	US CDC^20^
40-49 y	51.7 (22.2-44.1)	US CDC^20^
50-59 y	44.1 (24.0-64.0)	US CDC^20^
60-69 y	51.9 (38.3-66.0)	US CDC^20^
70-79 y	39.4 (27.1-52.5)	US CDC^20^
**CKD mortality parameters, hazard ratio**		
Mortality risk, CKD stage G3a	1.2 (1.1-1.3)	Go et al,^30^ 2004
Mortality risk, CKD stage G3b	1.8 (1.7-1.9)	Go et al,^30^ 2004
Mortality risk, CKD stage G4	3.2 (3.0-3.4)	Go et al,^30^ 2004
Mortality risk, kidney failure not requiring KRT	3.2 (3.0-3.4)	Go et al,^30^ 2004
Mortality risk, kidney failure requiring KRT	5.9 (5.4-6.4)	Go et al,^30^ 2004
**CKD quality-of-life adjustments for health states parameters**		
CKD stage G2	0.85 (0.70-0.96)	Cooper et al,^40^ 2020; and Goldhaber-Fiebert and Jalal,^51^ 2016
CKD stage G3a	0.81 (0.66-0.92)	Cooper et al,^40^ 2020; and Goldhaber-Fiebert and Jalal,^51^ 2016
CKD stage G3b	0.81 (0.66-0.92)	Cooper et al,^40^ 2020; and Goldhaber-Fiebert and Jalal,^51^ 2016
CKD stage G4	0.74 (0.61-0.85)	Cooper et al,^40^ 2020; and Goldhaber-Fiebert and Jalal,^51^ 2016
Kidney failure not requiring KRT	0.74 (0.61-0.85)	Cooper et al,^40^ 2020; and Goldhaber-Fiebert and Jalal,^51^ 2016
Kidney failure requiring KRT	0.60 (0.51-0.68)	Cooper et al,^40^ 2020; and Goldhaber-Fiebert and Jalal,^51^ 2016
**CKD stage–specific cost parameters, $**		
Monthly added cost of nondiabetic CKD stage G3a	32 (24-41)	Nichols et al,^32^ 2020; and Sanders et al,^36^ 2016
Monthly added cost of nondiabetic CKD stage G3b	108 (80-140)	Nichols et al,^32^ 2020; and Sanders et al,^36^ 2016
Monthly added cost of nondiabetic CKD stage G4	463 (346-598)	Nichols et al,^32^ 2020; and Sanders et al,^36^ 2016
Monthly added cost of kidney failure not requiring KRT among nondiabetic CKD population	463 (346-598)	Nichols et al,^32^ 2020; and Sanders et al,^36^ 2016
Monthly added cost of diabetic CKD stage G3a	438 (243-688)	Nichols et al,^32^ 2020; and Sanders et al,^36^ 2016
Monthly added cost of diabetic CKD stage G3b	806 (445-806)	Nichols et al,^32^ 2020; and Sanders et al,^36^ 2016
Monthly added cost of diabetic CKD stage G4	1824 (1022-2953)	Nichols et al,^32^ 2020; and Sanders et al,^36^ 2016
Monthly added cost of kidney failure not requiring KRT among diabetic CKD population	1824 (1022-2953)	Nichols et al,^32^ 2020; and Sanders et al,^36^ 2016
Monthly added cost of kidney failure requiring KRT	7020 (5217-8824)	Nichols et al,^32^ 2020; and Sanders et al,^36^ 2016
Monthly added cost of self-reported diabetes (undetected CKD stage G3a)	405 (302-526)	Nichols et al,^32^ 2020; and Sanders et al,^36^ 2016
Monthly added cost of self-reported diabetes (undetected CKD stage G3b)	684 (509-887)	Nichols et al,^32^ 2020; and Sanders et al,^36^ 2016
Monthly added cost of self-reported diabetes (undetected CKD stage G4)	1360 (1009-1752)	Nichols et al,^32^ 2020; and Sanders et al,^36^ 2016
Monthly added cost of self-reported diabetes (undetected kidney failure not requiring KRT)	1360 (1009-1752)	Nichols et al,^32^ 2020; and Sanders et al,^36^ 2016
Baseline costs	AHRQ × US expenditure Table [2013 converted to 2024] (75%-25%)	AHRQ^56^
**CKD stage-specific self-reported diabetes prevalence, %**		
Hispanic		
CKD stage G3a	27.4 (19.7-35.2)	US CDC^20^
CKD stage G3b	61.1 (48.1-74.1)	US CDC^20^
CKD stage G4	55.5 (34.6-73.1)	US CDC^20^
Non-Hispanic Black		
CKD stage G3a	27.3 (22.5-32.4)	US CDC^20^
CKD stage G3b	33.2 (25.0-40.9)	US CDC^20^
CKD stage G4	51.6 (35.7-66.7)	US CDC^20^
Non-Hispanic White		
CKD stage G3a	23.3 (19.5-27.2)	US CDC^20^
CKD stage G3b	34.4 (28.0-41.1)	US CDC^20^
CKD stage G4)	32.1 (19.0-47.6)	US CDC^20^
Additional racial and ethnic groups^a^		
CKD stage G3a	42.4 (31.3-53.8)	US CDC^20^
CKD stage G3b	51.4 (28.6-71.4)	US CDC^20^
CKD stage G4	64.5 (30.0-90.0)	US CDC^20^
Calibration parameters	NA	Cusick et al,^17^ 2023; and US CDC^20^

Abbreviations: ACE, angiotensin-converting enzyme; AHRQ, Agency for Healthcare Research and Quality; ARB, angiotensin receptor blocker; CDC, Centers for Disease Control and Prevention; CKD, chronic kidney disease; GFR, glomerular filtration rate; KRT, kidney replacement therapy; NA, not applicable; SGLT2, sodium-glucose cotransporter 2; UACR, urine-albumin creatinine ratio; UI, uncertainty interval.

^a^ Additional racial and ethnic groups includes those self-identifying as non-Hispanic Asian or other race (including multiracial).

#### Sensitivity Analysis

We conducted univariate sensitivity analyses using ranges reported in Table 1 stratified by racial and ethnic group and for the overall population.^60^ In additional sensitivity analyses, we assessed model outcomes under simultaneous SGLT2 inhibitor cost reductions and changes to SGLT2 inhibitor effectiveness in reducing all-cause mortality and slowing CKD progression (eMethods and eTable 4 in Supplement 1).^61^ In interpreting sensitivity analyses, we referenced a conventional cost-effectiveness threshold of $150 000/QALY gained.^62,63,64,65^

## Results

Our calibrated model produced high concordance with age-specific estimates of CKD prevalence, detection, and treatment across racial and ethnic groups (eTables 5-21 and eFigures 1-5 in Supplement 1). Cumulative incidence of kidney failure requiring KRT matched racial and ethnic group–specific estimates from published literature (eMethods in Supplement 1).^4^

### Effectiveness

#### Cumulative Incidence of Kidney Failure Requiring KRT

In the status quo scenario without screening and with treatment with conventional CKD therapy, overall lifetime cumulative incidence of kidney failure requiring KRT for 35-year-olds was 3.2% (95% UI, 1.8%-4.9%), with non-Hispanic Black 35-year-olds experiencing the highest average incidence at 6.2% (95% UI, 2.8%-10.6%), compared with Hispanic adults (3.6% [95% UI, 1.1%-6.7%]), non-Hispanic White adults (2.3% [95% UI, 0.4%-5.2%]), and adults from additional racial and ethnic groups (3.3% [95% UI, 1.2%-6.5%]) (Table 2). The largest average gap in cumulative incidence of kidney failure requiring KRT was between non-Hispanic Black and non-Hispanic White adults, with a gap of 4.0 percentage points (pp) (95% UI, −0.6 to 8.7 pp).

**Table 2.** Main Outcomes for Key Screen-and-Treat Interventions Across the Overall Population and 4 Racial and Ethnic Groups

Outcome	Status quo with ACE inhibitors or ARBs	With SGLT2 inhibitors^a^			
		Screen every 10 y, ages 55-75 y	Screen every 5 y, ages 55-75 y	Screen every 5 y, ages 45-75 y	Screen every 5 y, ages 35-75 y
**Cumulative incidence of kidney failure requiring KRT, %**					
Hispanic	3.56 (1.10-6.65)	2.96 (0.78-5.89)	2.91 (0.77-5.82)	2.88 (0.77-5.77)	2.86 (0.76-5.75)
Non-Hispanic Black	6.23 (2.81-10.62)	5.51 (2.29-9.67)	5.44 (2.25-9.58)	5.36 (2.20-9.51)	5.29 (2.17-9.45)
Non-Hispanic White	2.29 (0.36-5.17)	1.94 (0.29-4.54)	1.91 (0.29-4.47)	1.89 (0.29-4.43)	1.87 (0.28-4.39)
Additional groups^b^	3.30 (1.16-6.5)	2.72 0.84-5.59)	2.67 (0.83-5.53)	2.64 (0.81-5.49)	2.61 (0.80-5.46)
Overall	3.16 (1.78-4.93)	2.69 (1.41-4.39)	2.65 (1.39-4.33)	2.61 (1.37-4.29)	2.59 (1.35-4.26)
**Discounted life-years**					
Hispanic	24.63 (24.51-24.73)	24.74 (24.61-24.86)	24.74 (24.62-24.86)	24.75 (24.62-24.87)	24.75 (24.62-24.88)
Non-Hispanic Black	22.41 (22.25-22.56)	22.59 (22.40-22.76)	22.60 (22.41-22.77)	22.62 (22.43-22.80)	22.63 (22.44-22.82)
Non-Hispanic White	23.80 (23.69-23.90)	23.91 (23.79-24.03)	23.92 (23.80-24.04)	23.92 (23.80-24.04)	23.93 (23.80-24.05)
Additional groups^b^	24.82 (24.70-24.92)	24.92 (24.80-25.03)	24.93 (24.80-25.04)	24.93 (24.81-25.05)	24.93 (24.81-25.05)
Overall	23.90 (23.82-23.97)	24.01 (23.92-24.11)	24.02 (23.92-24.12)	24.03 (23.93-24.13)	24.03 (23.93-24.14)
**Discounted QALYs**					
Hispanic	20.10 (19.03-20.84)	20.18 (19.10-20.93)	20.18 (19.11-20.94)	20.19 (19.11-20.94)	20.19 (19.12-20.95)
Non-Hispanic Black	17.57 (15.91-18.78)	17.69 (16.02-18.90)	17.70 (16.02-18.92)	17.72 (16.04-18.94)	17.73 (16.05-18.95)
Non-Hispanic White	18.97 (17.46-20.03)	19.05 (17.52-20.11)	19.05 (17.52-20.12)	19.06 (17.53-20.12)	19.06 (17.53-20.13)
Additional groups^b^	20.06 (18.78-20.95)	20.13 (18.83-21.03)	20.13 (18.84-21.03)	20.14 (18.84-21.04)	20.14 (18.85-21.04)
Overall	19.14 (17.75-20.12)	19.22 (17.81-20.21)	19.22 (17.81-20.22)	19.23 (17.82-20.22)	19.24 (17.83-20.23)
**Discounted health care costs, $**					
Hispanic	256 200 (192 400-319 500)	260 600 (196 200-324 600)	261 200 (196 600-325 300)	262 200 (197 600-326 000)	263 200 (198 400-327 100)
Non-Hispanic Black	232 100 (176 500-287 000)	237 800 (181 300-293 400)	238 500 (181 900-294 200)	240 000 (183 400-295 700)	241 300 (184 700-296 900)
Non-Hispanic White	239 900 (179 200-299 600)	244 400 (183 000-304 400)	245 000 (183 500-305 100)	246 100 (184 600-306 500)	247 200 (185 700-307 900)
Additional groups^b^	260 400 (195 500-324 500)	264 500 (198 900-328 900)	265 000 (199 500-329 500)	266 000 (200 600-330 500)	267 000 (201 600-331 600)
Overall	242 300 (181 800-301 900)	236 900 (185 600-306 900)	247 500 (186 100-307 600)	248 700 (187 300-309 000)	249 800 (188 400-310 200)

Abbreviations: ACE, angiotensin-converting enzyme; ARB, angiotensin receptor blocker; KRT, kidney replacement therapy; SGLT2, sodium-glucose cotransporter 2; QALY, quality-adjusted life-year.

^a^ Addition of SGLT2 inhibitors to ACE inhibitors or ARBs.

^b^ Additional racial and ethnic groups includes those self-identifying as non-Hispanic Asian or other race (including multiracial).

**Figure 1.**
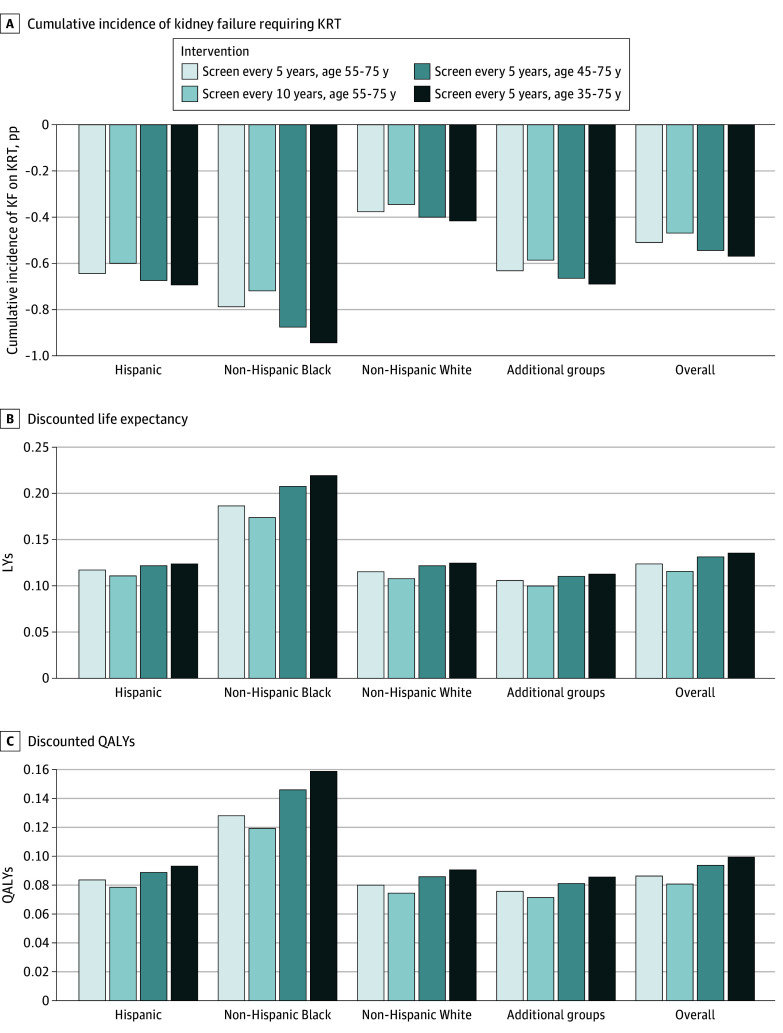
Changes in Main Outcomes From Select Population-Wide Chronic Kidney Disease Screen-and-Treat Interventions Compared With Status Quo for the Overall Population and Across 4 Racial and Ethnic Groups The status quo was defined as case detection and treatment with conventional chronic kidney disease therapy (angiotensin-converting enzyme inhibitors or angiotensin receptor blockers). Intervention was defined as periodic screening with the addition of sodium-glucose cotransporter 2 inhibitor therapy to conventional chronic kidney disease therapy. Additional racial and ethnic groups included Asian individuals, multiracial individuals, and those with other race or ethnicity. KRT indicates kidney replacement therapy; LY, life-year; pp, percentage point; QALY, quality-adjusted life-year.

Population-wide screening reduced the cumulative incidence of kidney failure requiring KRT across the overall population and all racial and ethnic groups (Figure 1). Under screening every 5 years from ages 55 to 75 years, cumulative incidence of kidney failure requiring KRT across the overall population decreased on average by 0.5 (95% UI, −0.1 to 1.2) pp to 2.7% (95% UI, 1.4% to 4.3%), compared with the status quo. Average reductions were larger among Hispanic adults (−0.7 [95% UI, −1.7 to 0.2] pp), non-Hispanic Black adults (−0.8 [95% UI, −2.3 to 0.6] pp), and adults from additional racial and ethnic group adults (−0.6 [95% UI, −1.6 to 0.0] pp) compared with non-Hispanic White adults (−0.4 [95% UI, −1.1 to 0.1] pp), reducing disparities in expected KRT incidence (Table 2). Initiating screening every 5 years at age 35 years led to an average projected reduction of 1.0 (95% UI, −0.4 to 2.5) pp compared with status quo for non-Hispanic Black adults, further reducing disparities.

#### Averted Cases of Kidney Failure Requiring KRT

Under status quo, we projected 4.7 million total lifetime cases of kidney failure requiring KRT among US adults currently aged between 35 and 75 years, with Hispanic adults, non-Hispanic Black adults, and adults from additional racial and ethnic groups disproportionately affected. While non-Hispanic Black adults make up 12% of the population, they accounted for 24% of KRT cases (eTable 22 in Supplement 1). Implementing screening every 5 years from age 55 to 75 years was projected to avert a total of 689 000 cases across the overall population compared with status quo. This strategy was estimated to prevent more cases of KRT per 10 000 persons screened among Hispanic adults (62), non-Hispanic Black adults (68), and adults from additional racial and ethnic groups (58) compared with non-Hispanic White adults (33) (eTable 22 in Supplement 1). Screening earlier, starting at age 35 years, was projected to prevent an additional 27 000 cases, with one-third of these cases among non-Hispanic Black adults.

#### Life Expectancy and QALYs

Under status quo, discounted LYs and QALYs for 35-year-old adults were 23.90 (95% UI, 23.82-23.97) LYs and 19.14 (95% UI, 17.75-20.12) QALYs, respectively (Table 2). The largest disparities in life expectancy were observed between non-Hispanic Black adults (22.41 LYs; 95% UI, 22.25-22.56 LYs) and adults from additional racial and ethnic groups (24.82 LYs; 95% UI, 24.70-24.92 LYs) (Table 2), with a difference of 2.41 (95% UI, 2.22-2.60) LYs.

With screening every 5 years from ages 55 to 75 years, overall LYs and QALYs increased for all racial and ethnic groups, with the largest gains observed among non-Hispanic Black adults (Figure 1 and Table 2). Screening earlier at age 45 or 35 years produced additional gains in LYs and QALYs for all racial and ethnic groups and narrowed gaps in expected LYs between non-Hispanic Black adults and non-Hispanic White adults by 0.08 (95% UI, 0-0.19) LYs compared with status quo (Table 2).

### Cost-Effectiveness

Adding screen-and-treat interventions for CKD increased health care costs across all racial and ethnic groups. For a 35-year-old, adding screening every 5 years from 55 to 75 years increased health care costs on average between $4600 and $6400, depending on the racial and ethnic group (Table 2).

**Figure 2.**
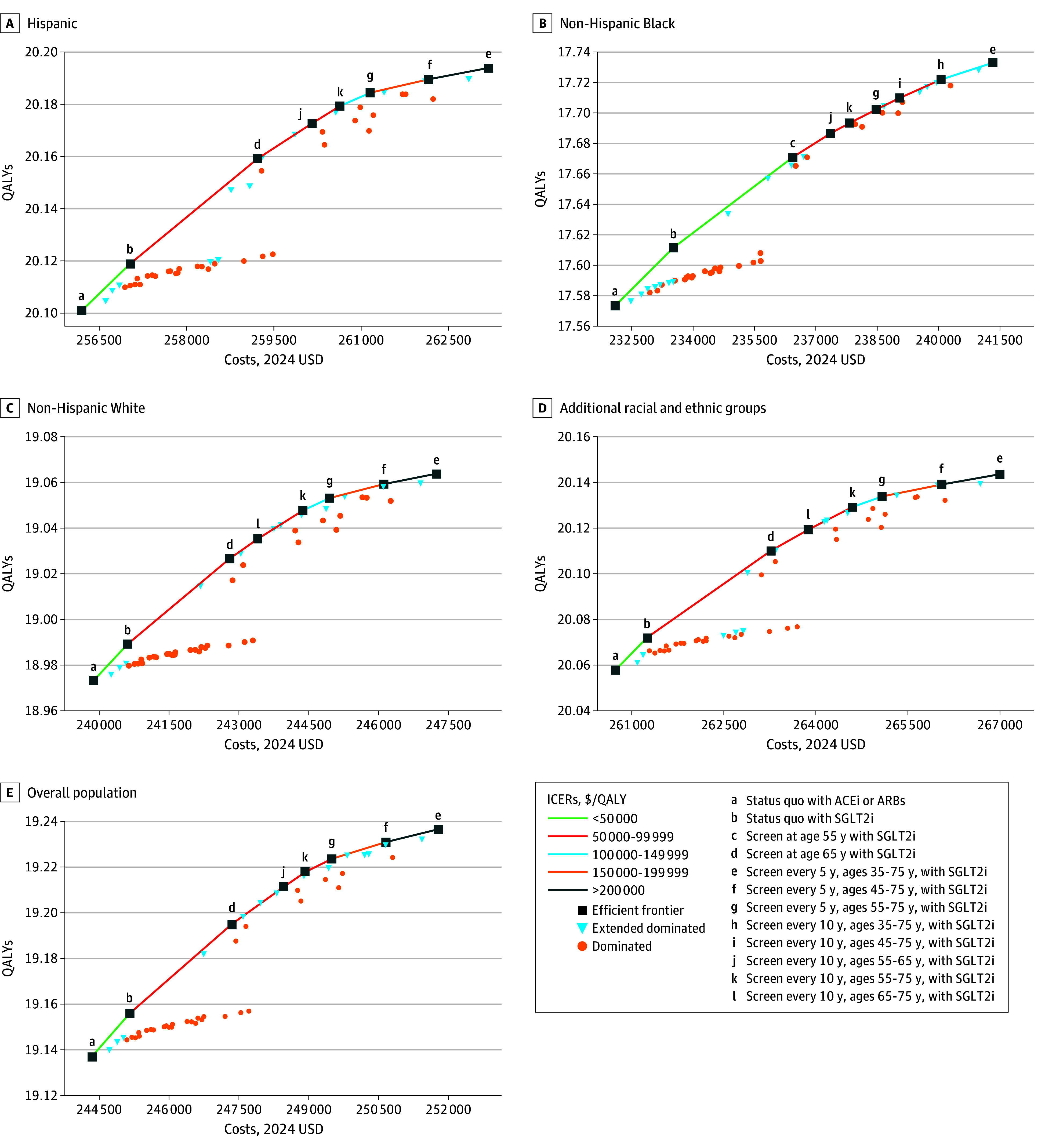
Cost-Effectiveness Planes for the 35-Year-Old Cohort Across 4 Racial and Ethnic Groups and the Overall Population Additional racial and ethnic groups included Asian individuals, multiracial individuals, and those identifying as other race. With SGLT2i is the addition of sodium glucose cotransporter 2 inhibitors (SGLT2i) to angiotensin-converting enzyme inhibitor (ACEi) or angiotensin receptor blocker (ARB) therapy. ICER indicates incremental cost-effectiveness ratio; QALY, quality-adjusted life-year. Cost-effectiveness tables are reported in eTable 23 in Supplement 1.

For 35-year-olds across the overall population, initiating screening every 5 years from age 55 to 75 years cost $99 100 per QALY gained compared with less frequent screening every 10 years from age 55 to 75 years (eTable 23 in Supplement 1). Across racial and ethnic groups, screening every 5 years from age 55 to 75 years cost less than $150 000/QALY gained, with non-Hispanic Black adults having the lowest cost per QALY gained at $73 400 (Figure 2; eTable 23 in Supplement 1). Results for the 45- to 75-year-old cohorts are provided in eTables 24 to 27 in Supplement 1. Initiating screening every 5 years at younger ages (eg, 45 or 35 years), increased ICERs, exceeding $150 000 per QALY gained for Hispanic adults, non-Hispanic White adults, and adults from additional racial and ethnic groups. For non-Hispanic Black adults, earlier screening at age 35 years remained cost-effective at $115 000/QALY gained (Figure 2; eTable 23 in Supplement 1).

### Sensitivity Analyses

**Figure 3.**
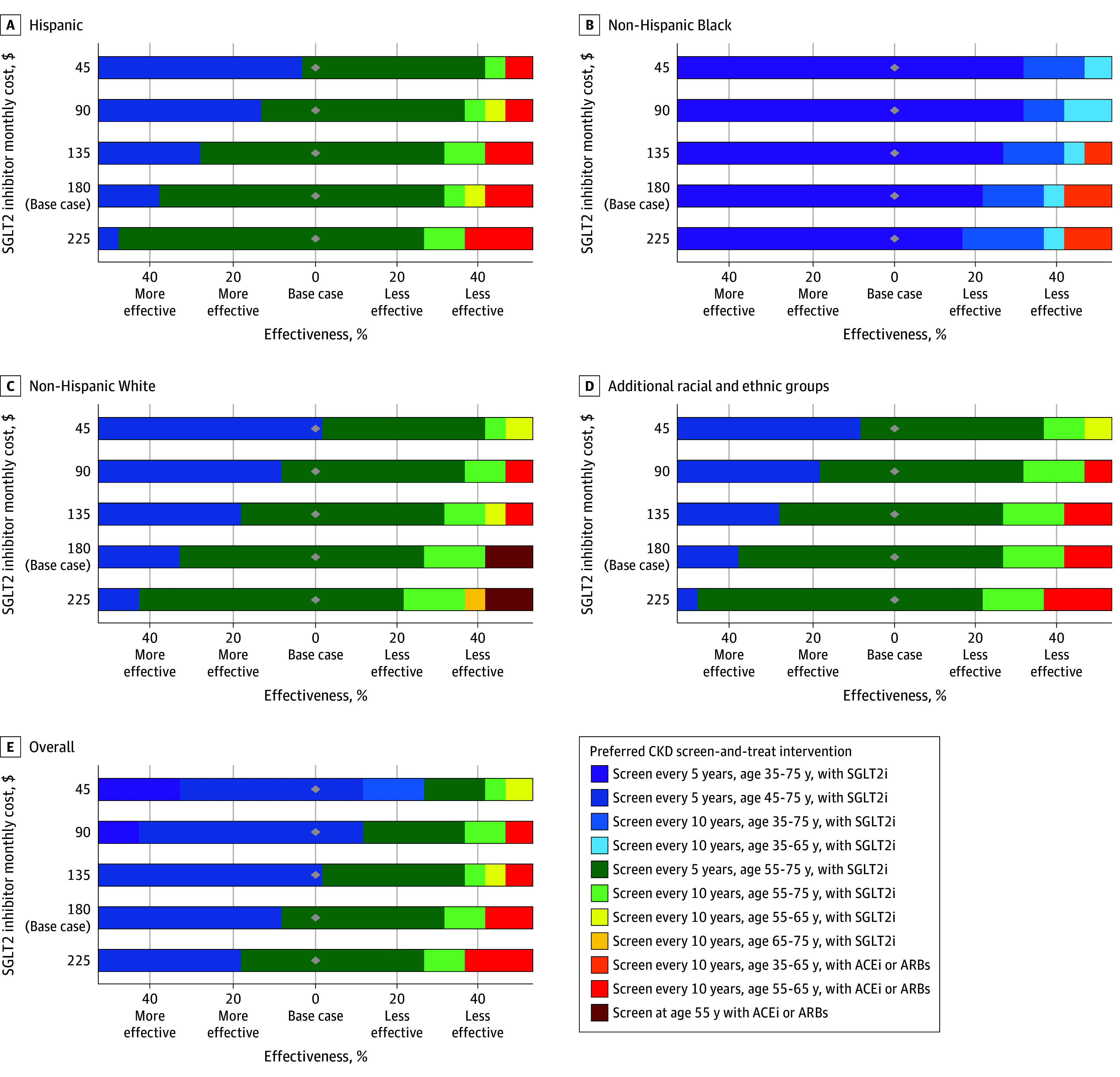
Preferred Population-Wide Chronic Kidney Disease (CKD) Screen-and-Treat Interventions Under 2-Way Sensitivity Analysis on Sodium-Glucose Cotransporter Inhibitor (SGLT2i) Effectiveness and Costs for the Overall Population and Across 4 Racial and Ethnic Groups Additional racial and ethnic groups included Asian individuals, multiracial individuals, and those identifying as other race. With SGLT2i is the addition of SGLT2is to angiotensin-converting enzyme inhibitor (ACEi) or angiotensin receptor blocker (ARB) therapy. Preferred population-wide CKD screen-and-treat interventions were evaluated at a willingness-to-pay threshold of $150 000 per quality-adjusted life-year gained.

Across the overall population and within each racial and ethnic group, the cost-effectiveness of population-wide screening was driven by the effectiveness of SGLT2 inhibitors in reducing all-cause mortality and slowing CKD progression, eGFR stage–specific quality-of-life weights, SGLT2 inhibitor and conventional CKD therapy costs, and treatment initiation after screening (eFigures 6-8 in Supplement 1). If SGLT2 inhibitor effectiveness were reduced by 35%, the cost-effectiveness of screening every 5 years from age 55 to 75 years increased to $157 800/QALY gained for the overall population, exceeding $150 000/QALY gained for all racial and ethnic groups except non-Hispanic Black adults. SGLT2 inhibitor price reductions would make initiation of screening at younger ages more favorable (Figure 3). Under base case SGLT2 inhibitor effectiveness and a 50% reduction in SGLT2 inhibitor costs ($90 per month), screening every 5 years from age 45 to 75 years cost $132 900/QALY gained for the overall population. Under this scenario, the most favorable results were observed for non-Hispanic Black adults ($67 500/QALY gained), while the least favorable were for adults from additional racial and ethnic groups ($172 200/QALY gained) (Figure 3).

## Discussion

In the era of SGLT2 inhibitor use, population-wide screening for CKD can increase life expectancy and reduce the burden of kidney failure requiring KRT across the population for all racial and ethnic groups. Projected health benefits from screening were greatest for non-Hispanic Black adults, who face the highest burden of kidney failure under status quo conditions. Across the overall population and all racial and ethnic groups, screening every 5 years from age 55 to 75 years was cost-effective at a threshold of $150 000/QALY gained. Population-wide screening strategies initiated at age 45 or 35 years were not cost-effective at this threshold yet produced additional population health gains and were more effective in narrowing disparities in health outcomes between racial and ethnic groups.

We observed differences in cost-effectiveness results across the racial and ethnic groups. While screening from age 35 or 45 years was cost-effective at a $150 000/QALY gained threshold for non-Hispanic Black adults, these strategies were not cost-effective in Hispanic adults, non-Hispanic White adults, and adults from additional racial and ethnic groups. Cost-effectiveness differences were largely driven by heterogeneity in kidney disease progression among racial and ethnic groups. Prior research has cited biological factors, including the prevalence of *APOL1* variants, as well as social factors, including socioeconomic status, health care access, and systemic racism, as factors that contribute to faster CKD progression among Hispanic and non-Hispanic Black adults.^8,9,66,67^

Interventions to improve screening among non-Hispanic Black adults will likely require addressing barriers at the individual, social, and system level. For example, patient- and community-level engagement may be valuable for implementing population-wide CKD screening.^68^ Public education programs, culturally appropriate messaging, online tools and mobile applications, community outreach and resources, support groups, and Indigenous health coaches can improve CKD knowledge and self-management.^69,70^ Such interventions have been effective in improving diabetes prevention and screening attendance across diverse populations.^71,72,73^ Research is needed to identify additional opportunities for intervention at the health system level.

### Limitations

Our study has several limitations. First, given insufficient data, we were unable to conduct specific analysis for American Indian and Alaska Native, Asian, and Native Hawaiian and Pacific Islander groups. Given heterogeneity in CKD prevalence and kidney failure risk for these groups, interpretability of results for the group labeled as additional racial and ethnic groups is limited.^4^ Second, due to insufficient data, our study did not stratify by sex, which could provide additional insights given sex-based differences in CKD prevalence and progression.^74^ Third, given limited data on the long-term effectiveness of SGLT2 inhibitors, our analysis depended on a single randomized clinical trial, DAPA-CKD.^15^ Other randomized clinical trials have tested the efficacy of SGLT2 inhibitors for CKD, yet these trials’ inclusion criteria, such as patients with and without albuminuria (EMPA-KIDNEY) and only patients with diabetes (CREDENCE and SCORED), did not allow us to incorporate these results.^75,76,77^ In sensitivity analyses, we considered a wide range of SGLT2 inhibitor effectiveness values, including those observed in these trials. Fourth, our model did not explicitly simulate incidence and prevalence of key comorbidities, including diabetes, hypertension, and cardiovascular disease. In prior work, the cost-effectiveness of CKD screening has been evaluated in populations with comorbidities, including our subgroup analysis by self-reported diabetes status.^17,78,79^ Inclusion of SGLT2 inhibitor effectiveness on reducing cardiovascular events (eg, nonfatal heart failure hospitalizations) and anemia-related events (eg, transfusion and use of erythropoiesis stimulating agents) would likely make screening more favorable across racial and ethnic groups, particularly for groups with higher burdens of cardiovascular disease and anemia.^80,81^ Fifth, due to data limitations in productivity and caregiver time, we did not conduct an analysis from the societal perspective. Sixth, we did not consider differential real-world implementation challenges across racial and ethnic groups, including health care access, take-up of screening, and treatment adherence, which could influence cost-effectiveness results.

## Conclusions

In this cost-effectiveness analysis, population-wide screening for CKD combined with ACE inhibitors or ARBs along with SGLT2 inhibitors over ages 55 to 75 years was estimated to have large population health benefits, was cost-effective, and narrowed CKD disparities across 4 racial and ethnic groups. Earlier initiation of screening at age 45 or 35 years yielded further benefits for all patient groups—in particular non-Hispanic Black adults—and additional reductions in CKD disparities but was not cost-effective for the overall population under base case assumptions. In determining the optimal population-wide screening strategy, policymakers should consider trade-offs between efficiency and equity. Tailored messaging and promotion for CKD screening engagement may be warranted for non-Hispanic Black adults, for whom screening may have the greatest benefit.

## Notes

Supplement 1.
eMethods.
**eTable 1.** Effective Sample Size by Racial and Ethnic Group **eTable 2.** eGFR G3a Specific Costs by Racial and Ethnic Group **eTable 3.** Probabilistic Sensitivity Analysis Input Table **eTable 4.** SGLT2 Inhibitor Hazard Ratio Ranges in Sensitivity Analyses **eTable 5.** Concordance Between Calibration Targets and Model Projections **eTable 6.** CKD Distribution Estimates and Calibration Targets (NHANES Estimates) and Calibrated Model Projections for Hispanic Adults Aged 40 to 49 Years **eTable 7.** CKD Distribution Estimates and Calibration Targets (NHANES Estimates) and Calibrated Model Projections for Hispanic Adults Aged 50 to 59 Years **eTable 8.** CKD Distribution Estimates and Calibration Targets (NHANES Estimates) and Calibrated Model Projections for Hispanic Adults Aged 60 to 69 Years **eTable 9.** CKD Distribution Estimates and Calibration Targets (NHANES Estimates) and Calibrated Model Projections for Hispanic Adults Aged 70 to 79 Years **eTable 10.** CKD Distribution Estimates and Calibration Targets (NHANES Estimates) and Calibrated Model Projections for Non-Hispanic Black Adults Aged 40 to 49 Years **eTable 11.** CKD Distribution Estimates and Calibration Targets (NHANES Estimates) and Calibrated Model Projections for Non-Hispanic Black Adults Aged 50 to 59 Years **eTable 12.** CKD Distribution Estimates and Calibration Targets (NHANES Estimates) and Calibrated Model Projections for Non-Hispanic Black Adults Aged 60 to 69 Years **eTable 13.** CKD Distribution Estimates and Calibration Targets (NHANES Estimates) and Calibrated Model Projections for Non-Hispanic Black Adults Aged 70 to 79 Years **eTable 14.** CKD Distribution Estimates and Calibration Targets (NHANES Estimates) and Calibrated Model Projections for Non-Hispanic White Adults Aged 40 to 49 Years **eTable 15.** CKD Distribution Estimates and Calibration Targets (NHANES Estimates) and Calibrated Model Projections for Non-Hispanic White Adults Aged 50 to 59 Years **eTable 16.** CKD Distribution Estimates and Calibration Targets (NHANES Estimates) and Calibrated Model Projections for Non-Hispanic White Adults Aged 60 to 69 Years **eTable 17.** CKD Distribution Estimates and Calibration Targets (NHANES Estimates) and Calibrated Model Projections for Non-Hispanic White Adults Aged 70 to 79 Years **eTable 18.** CKD Distribution Estimates and Calibration Targets (NHANES Estimates) and Calibrated Model Projections for Adults From Additional Racial and Ethnic Groups Aged 40 to 49 Years **eTable 19.** CKD Distribution Estimates and Calibration Targets (NHANES Estimates) and Calibrated Model Projections for Adults From Additional Racial and Ethnic Groups Aged 50 to 59 Years **eTable 20.** CKD Distribution Estimates and Calibration Targets (NHANES Estimates) and Calibrated Model Projections for Adults From Additional Racial and Ethnic Groups Aged 60 to 69 Years **eTable 21.** CKD Distribution Estimates and Calibration Targets (NHANES Estimates) and Calibrated Model Projections for Adults From Additional Racial and Ethnic Groups Aged 70 to 79 Years **eFigure 1.** Calibrated eGFR Stage Marginal Distribution Ribbon Plot for Hispanic Adults, Non-Hispanic Black Adults, Non-Hispanic White Adults, and Adults From Additional Racial and Ethnic Groups **eFigure 2.** Calibrated Stage 5 (eGFR <15) and Kidney Failure Receiving Kidney Replacement Therapy Marginal Distribution Ribbon Plot for Hispanic Adults, Non-Hispanic Black Adults, Non-Hispanic White Adults, and Adults From Additional Racial and Ethnic Groups **eFigure 3.** Calibrated Albuminuria Stage Prevalence Marginal Distribution Ribbon plot for Hispanic Adults, Non-Hispanic Black Adults, Non-Hispanic White Adults, and Adults From Additional Racial and Ethnic Groups **eFigure 4.** Calibrated CKD Detection/Awareness Prevalence Marginal Distribution Ribbon Plot for Hispanic Adults, Non-Hispanic Black Adults, Non-Hispanic White Adults, and Adults From Additional Racial and Ethnic Groups **eFigure 5.** Calibrated CKD Detection and Treatment Marginal Distribution Ribbon Plot for Hispanic Adults, Non-Hispanic Black Adults, Non-Hispanic White Adults, and Adults From Additional Racial and Ethnic Groups **eTable 22.** Projected Cases of Kidney Failure (KF) Requiring Kidney Replacement Therapy (KRT) in the US Population by Racial and Ethnic Group **eTable 23.** Cost-Effectiveness Table for the 35-Year-Old Cohort Across Racial and Ethnic Groups **eTable 24.** Cost-Effectiveness Table for the 45-Year-Old Cohort Across Racial and Ethnic Groups **eTable 25.** Cost-Effectiveness Table for the 55-Year-Old Cohort Across Racial and Ethnic Groups **eTable 26.** Cost-Effectiveness Table for the 65-Year-Old Cohort Across Racial and Ethnic Groups **eTable 27.** Cost-Effectiveness Table for the 75-Year-Old Cohort Across Racial and Ethnic Groups **eFigure 6. **Univariate Sensitivity Analysis for Every 5 Years Screening From Ages 55 to 75 Years With SGLT2i for Hispanic Adults, Non-Hispanic Black Adults, Non-Hispanic White Adults, Adults From Additional Racial and Ethnic Groups, and the Overall Population Aged 35 Years **eFigure 7. **Univariate Sensitivity Analysis for Every 5 Years Screening From Ages 45 to 75 Years With SGLT2i for Hispanic Adults, Non-Hispanic Black Adults, Non-Hispanic White Adults, Adults From Additional Racial and Ethnic Groups, and the Overall Population Aged 35 Years **eFigure 8. **Univariate Sensitivity Analysis for Every 5 Years Screening From Ages 35 to 75 Years With SGLT2i for Hispanic Adults, Non-Hispanic Black Adults, Non-Hispanic White Adults, Adults From Additional Racial and Ethnic Groups, and the Overall Population Aged 35 Years
eReferences.

Supplement 2.
Data Sharing Statement
