# Supplementary material for: Balancing Efficiency and Equity in Population-Wide CKD Screening
Source: JAMA Netw Open. 2025 Apr 14;8(4):e254740. doi: 10.1001/jamanetworkopen.2025.4740 (PMC11997725; doi:10.1001/jamanetworkopen.2025.4740)
Supplement: Supplement 1. — eMethods. eTable 1. Effective Sample Size by Racial and Ethnic Group eTable 2. eGFR G3a Specific Costs by Racial and Ethnic Group eTable 3. Probabilistic Sensitivity Analysis Input Table eTable 4. SGLT2 Inhibitor Hazard Ratio Ranges in Sensitivity Analyses eTable 5. Concordance Between Calibration Targets and Model Projections eTable 6. CKD Distribution Estimates and Calibration Targets (NHANES Estimates) and Calibrated Model Projections for Hispanic Adults Aged 40 to 49 Years eTable 7. CKD Distribution Estimates and Calibration Targets (NHANES Estimates) and Calibrated Model Projections for Hispanic Adults Aged 50 to 59 Years eTable 8. CKD Distribution Estimates and Calibration Targets (NHANES Estimates) and Calibrated Model Projections for Hispanic Adults Aged 60 to 69 Years eTable 9. CKD Distribution Estimates and Calibration Targets (NHANES Estimates) and Calibrated Model Projections for Hispanic Adults Aged 70 to 79 Years eTable 10. CKD Distribution Estimates and Calibration Targets (NHANES Estimates) and Calibrated Model Projections for Non-Hispanic Black Adults Aged 40 to 49 Years eTable 11. CKD Distribution Estimates and Calibration Targets (NHANES Estimates) and Calibrated Model Projections for Non-Hispanic Black Adults Aged 50 to 59 Years eTable 12. CKD Distribution Estimates and Calibration Targets (NHANES Estimates) and Calibrated Model Projections for Non-Hispanic Black Adults Aged 60 to 69 Years eTable 13. CKD Distribution Estimates and Calibration Targets (NHANES Estimates) and Calibrated Model Projections for Non-Hispanic Black Adults Aged 70 to 79 Years eTable 14. CKD Distribution Estimates and Calibration Targets (NHANES Estimates) and Calibrated Model Projections for Non-Hispanic White Adults Aged 40 to 49 Years eTable 15. CKD Distribution Estimates and Calibration Targets (NHANES Estimates) and Calibrated Model Projections for Non-Hispanic White Adults Aged 50 to 59 Years eTable 16. CKD Distribution Estimates and Calibration Targets (NHANES Estim [file jamanetwopen-e254740-s001.pdf]

Supplemental Online Content

Cusick MM, Tisdale RL, Adams AS, et al. Balancing efficiency and equity in population-wide CKD screening. *JAMA Netw Open.* 2025;8(4):e254740. doi:10.1001/jamanetworkopen.2025.4740

eMethods.

eTable 1. Effective Sample Size by Racial and Ethnic Group

eTable 2. eGFR G3a Specific Costs by Racial and Ethnic Group

eTable 3. Probabilistic Sensitivity Analysis Input Table

eTable 4. SGLT2 Inhibitor Hazard Ratio Ranges in Sensitivity Analyses

eTable 5. Concordance Between Calibration Targets and Model Projections

eTable 6. CKD Distribution Estimates and Calibration Targets (NHANES Estimates) and Calibrated Model Projections for Hispanic Adults Aged 40 to 49 Years

eTable 7. CKD Distribution Estimates and Calibration Targets (NHANES Estimates) and Calibrated Model Projections for Hispanic Adults Aged 50 to 59 Years

eTable 8. CKD Distribution Estimates and Calibration Targets (NHANES Estimates) and Calibrated Model Projections for Hispanic Adults Aged 60 to 69 Years

eTable 9. CKD Distribution Estimates and Calibration Targets (NHANES Estimates) and Calibrated Model Projections for Hispanic Adults Aged 70 to 79 Years

eTable 10. CKD Distribution Estimates and Calibration Targets (NHANES Estimates) and Calibrated Model Projections for Non-Hispanic Black Adults Aged 40 to 49 Years

eTable 11. CKD Distribution Estimates and Calibration Targets (NHANES Estimates) and Calibrated Model Projections for Non-Hispanic Black Adults Aged 50 to 59 Years

eTable 12. CKD Distribution Estimates and Calibration Targets (NHANES Estimates) and Calibrated Model Projections for Non-Hispanic Black Adults Aged 60 to 69 Years

eTable 13. CKD Distribution Estimates and Calibration Targets (NHANES Estimates) and Calibrated Model Projections for Non-Hispanic Black Adults Aged 70 to 79 Years

eTable 14. CKD Distribution Estimates and Calibration Targets (NHANES Estimates) and Calibrated Model Projections for Non-Hispanic White Adults Aged 40 to 49 Years

eTable 15. CKD Distribution Estimates and Calibration Targets (NHANES Estimates) and Calibrated Model Projections for Non-Hispanic White Adults Aged 50 to 59 Years

eTable 16. CKD Distribution Estimates and Calibration Targets (NHANES Estimates) and Calibrated Model Projections for Non-Hispanic White Adults Aged 60 to 69 Years

© 2025 Cusick MM et al. JAMA Network Open.

eTable 17. CKD Distribution Estimates and Calibration Targets (NHANES Estimates) and Calibrated Model Projections for Non-Hispanic White Adults Aged 70 to 79 Years

eTable 18. CKD Distribution Estimates and Calibration Targets (NHANES Estimates) and Calibrated Model Projections for Adults From Additional Racial and Ethnic Groups Aged 40 to 49 Years

eTable 19. CKD Distribution Estimates and Calibration Targets (NHANES Estimates) and Calibrated Model Projections for Adults From Additional Racial and Ethnic Groups Aged 50 to 59 Years

eTable 20. CKD Distribution Estimates and Calibration Targets (NHANES Estimates) and Calibrated Model Projections for Adults From Additional Racial and Ethnic Groups Aged 60 to 69 Years

eTable 21. CKD Distribution Estimates and Calibration Targets (NHANES Estimates) and Calibrated Model Projections for Adults From Additional Racial and Ethnic Groups Aged 70 to 79 Years

eFigure 1. Calibrated eGFR Stage Marginal Distribution Ribbon Plot for Hispanic Adults, Non-Hispanic Black Adults, Non-Hispanic White Adults, and Adults From Additional Racial and Ethnic Groups

eFigure 2. Calibrated Stage 5 (eGFR <15) and Kidney Failure Receiving Kidney Replacement Therapy Marginal Distribution Ribbon Plot for Hispanic Adults, Non-Hispanic Black Adults, Non-Hispanic White Adults, and Adults From Additional Racial and Ethnic Groups

eFigure 3. Calibrated Albuminuria Stage Prevalence Marginal Distribution Ribbon plot for Hispanic Adults, Non-Hispanic Black Adults, Non-Hispanic White Adults, and Adults From Additional Racial and Ethnic Groups

eFigure 4. Calibrated CKD Detection/Awareness Prevalence Marginal Distribution Ribbon Plot for Hispanic Adults, Non-Hispanic Black Adults, Non-Hispanic White Adults, and Adults From Additional Racial and Ethnic Groups

eFigure 5. Calibrated CKD Detection and Treatment Marginal Distribution Ribbon Plot for Hispanic Adults, Non-Hispanic Black Adults, Non-Hispanic White Adults, and Adults From Additional Racial and Ethnic Groups

eTable 22. Projected Cases of Kidney Failure (KF) Requiring Kidney Replacement Therapy (KRT) in the US Population by Racial and Ethnic Group

eTable 23. Cost-Effectiveness Table for the 35-Year-Old Cohort Across Racial and Ethnic Groups

eTable 24. Cost-Effectiveness Table for the 45-Year-Old Cohort Across Racial and Ethnic Groups

eTable 25. Cost-Effectiveness Table for the 55-Year-Old Cohort Across Racial and Ethnic Groups

eTable 26. Cost-Effectiveness Table for the 65-Year-Old Cohort Across Racial and Ethnic Groups

© 2025 Cusick MM et al. JAMA Network Open.

eTable 27. Cost-Effectiveness Table for the 75-Year-Old Cohort Across Racial and Ethnic Groups

eFigure 6. Univariate Sensitivity Analysis for Every 5 Years Screening From Ages 55 to 75 Years With SGLT2i for Hispanic Adults, Non-Hispanic Black Adults, Non-Hispanic White Adults, Adults From Additional Racial and Ethnic Groups, and the Overall Population Aged 35 Years

eFigure 7. Univariate Sensitivity Analysis for Every 5 Years Screening From Ages 45 to 75 Years With SGLT2i for Hispanic Adults, Non-Hispanic Black Adults, Non-Hispanic White Adults, Adults From Additional Racial and Ethnic Groups, and the Overall Population Aged 35 Years

eFigure 8. Univariate Sensitivity Analysis for Every 5 Years Screening From Ages 35 to 75 Years With SGLT2i for Hispanic Adults, Non-Hispanic Black Adults, Non-Hispanic White Adults, Adults From Additional Racial and Ethnic Groups, and the Overall Population Aged 35 Years

eReferences.

This supplemental material has been provided by the authors to give readers additional information about their work.

eMethods

Calibration target estimation

We split National Health and Nutrition Examination Survey (NHANES) data according to self-reported race/ethnicity into four distinct racial/ethnic groups: Hispanic, non-Hispanic Black, non-Hispanic white, and other race/ethnicity. We classified individuals self-reporting as Mexican American and other Hispanic as Hispanic, and given small sample sizes, individuals self-reporting as non-Hispanic Asian and other race (including multi-racial) as additional racial and ethnic groups.

We separately estimated race-specific calibration targets using a similar approach as in our original analysis (further described in the Supplement Methods 1 in Cusick et. al[1] for each of the four racial/ethnic groups. We selected all NHANES participants who were at least 30 years old with non-missing eGFR values. Sample sizes from 2013-2018 NHANES for Hispanic, non-Hispanic Black, non-Hispanic white, and additional racial and ethnic group adults were 2684, 3253, 4930, and 2067, respectively.

For each individual racial/ethnic group, we estimated age-specific eGFR stage marginal distributions (ordered logistic regression), age-eGFR-specific albuminuria stage marginal distributions (multinomial logistic regression), and age-eGFR-albuminuria-specific detection distributions (logistic regression) using the NHANES subset corresponding to each racial/ethnic group. Equations for these regressions (Equations 1-3) are provided below.

Equation 1

$$logit(P(Y \leq j)) = \beta_0 - (\beta_1 AgeGrp);\ Y = eGFR\ stage$$

Equation 2

$$logit\big(P(Y \leq j)\big) = \beta_0 - (\beta_1 AgeGrp - \beta_2 eGFR - \beta_3 I(Stage\ 3a) - \beta_4 I(Stage\ 3b) - \beta_5 I(Stage\ 4) - \beta_6 I(Stage\ 5));\ Y = Albuminuria\ stage$$

Equation 3

$$Y = \beta_0 + \beta_1 ns(age, 4) + \beta_2 eGFRStage + \beta_3 AlbuminuriaStage; Y = Detected$$

Age-eGFR-albuminuria-specific treatment distributions were estimated among those who responded yes to the NHANES survey question on ever being told about their weak or failing kidneys (detected for CKD). Due to sample size limitations in each individual race group who were detected for CKD, we were unable to produce stable and realistic estimates for age-eGFR-specific treatment distributions for each individual race group. We relied on the following logistic regression (Equation 4) to obtain these distributions, where the outcome was an individual's response to being treated now with anti-hypertensive medication (conventional CKD therapy).

Equation 4

$$Y = \beta_0 + \beta_1 ns(age, 4) + \beta_2 eGFRStage + \beta_3 AlbuminuriaStage + \beta_4 RaceGroup + \beta_5 RaceGroup{:}ns(age, 4)$$

$$Y = Treated\ Now$$

© 2025 Cusick MM et al. JAMA Network Open.

We fit our decision-analytic Markov model to simulate CKD progression in these four distinct racial/ethnic groups using Bayesian calibration (sampling-importance-resampling) using the same procedure as in our original analysis (further described in the Supplement Methods 1 in Cusick et. al.[1] To simulate adults in other racial/ethnic groups, we separately ran models using non-Hispanic Asian and of additional racial and ethnic group mortality rates. We combined model results according to a weighted average of those identifying as Non-Hispanic Asian and other race/ethnicity according to NHANES data. We computed multinomial logarithmic transformed likelihood scores with respect to the mean race-and-age-specific calibration target distribution obtained from NHANES and uncertainties determined by the effective sample size according to fitted Dirichlet distributions (eTable 1).

Life table estimation for additional racial and ethnic group

We relied on U.S. age, sex, race, and ethnicity-specific life tables for baseline mortality rates for Hispanic, non-Hispanic Black, and non-Hispanic white adults. Because U.S. life tables are not available for the additional racial and ethnic groups, we estimated these rates as a function of the overall age-sex-specific U.S. life table, age-sex-race-specific proportions of each available racial/ethnic group, and the annual estimates of the U.S. resident population by age, sex, race, and Hispanic origin.

CKD-stage-specific costs

CKD-stage-specific costs were computed between a weighted average of non-diabetic CKD and diabetic CKD costs according to self-reported diabetes prevalence, further described in Cusick et. al Supplement Methods 4.[1] Given differences in eGFR stage-specific self-reported diabetes prevalence estimates between race/ethnic groups in NHANES, we used differential prevalence estimates in our model, leading to different CKD stage-specific cost estimates for each race/ethnic group. We illustrate this in eTable 2 below specifically for eGFR Stage G3a, where base case non-diabetic CKD and diabetic CKD costs were $32 and $438, respectively.

Cases of KRT

We obtained the annual estimates of the resident population by single year of age according to the Census in 2019.[2] Next, we estimated the total proportion of the population that was each specific race/ethnicity group according to the Census breakdowns in 5-year age groups. We assumed that the proportions were constant within each race group.

Probabilistic Sensitivity Analysis

In probabilistic sensitivity analysis, we sampled 10,000 sets of model parameters from distributions according to eTable 3. We correlated eGFR stage-specific quality-of-life weights, costs, and self-reported diabetes prevalence to preserve rank ordering of eGFR-stages.[3] SGLT2 inhibitor effectiveness hazard ratios (HRs) in reducing all-cause mortality and slowing CKD progression were simultaneously sampled from a multivariate lognormal distribution. In deterministic univariate and bivariate sensitivity analyses, we grouped these parameters and modified them simultaneously.

Aggregated results for the overall population

Race/ethnic group results were aggregated to obtain results for the overall population using racial/ ethnic group-specific population proportions in the U.S. population. Racial/ethnic group-specific population proportions were 20.7%, 12.8%, 56.3%, and 10.0% for Hispanic, non-Hispanic Black, non-Hispanic white, and additional racial and ethnic group 35-year-olds according to the 2019 U.S. Census.[2]

Sensitivity analyses on SGLT2 inhibitor effectiveness

We evaluated the cost-effectiveness of SGLT2 inhibitors under varying levels of effectiveness. We modified effectiveness to range from 50% less effective to 50% more effective by applying percentage changes to the hazard ratios for SGLT2 inhibitor effects on all-cause mortality and kidney composite outcomes. Because SGLT2 inhibitor

© 2025 Cusick MM et al. JAMA Network Open.

effectiveness was computed as the average effectiveness across diabetic and non-diabetic groups, we modified effectiveness hazard ratios for both groups. The ranges of SGLT2 inhibitor effectiveness hazard ratios are in eTable 4.

Calibration results

Goodness of fit between our model projections and calibration targets was determined by overlap between the 95% uncertainty intervals (UI), defined as 95% interquartile interviews and the model's mean estimate being within the calibration target 95% UI. Age-specific 95% UIs from calibration model projections and NHANES calibration targets are available in eTables 6-21. We also generated marginal distribution ribbon plots (eFigures 1-5) for our model's projections with the NHANES calibration targets and 95% UIs shown as points with error bars for eGFR stage distribution, albuminuria stage distribution, and prevalence of population detected and treated for CKD.

Among all racial/ethnic groups, we achieved the highest concordance for the additional racial and ethnic group (overlap: 99%, model mean measure: 92%) and the lowest for the Non-Hispanic Black group (overlap 93%, model mean measure: 71%).

In a previous analysis, the lifetime risk of ESRD from birth was reported as 3.1% (95% CI: 3.0-3.1%) and 2.0% (95% CI: 2.0%, 2.1%) for Non-Hispanic white male and females; 8.0% (7.9%, 8.2%) and 6.8% (6.7%, 6.9%) for non-Hispanic Black male and females; 3.8% (95% CI: 3.4%, 4.9%) and 3.6% (95% CI: 3.3%, 4.2%) for NH Native American male and females; 5.1% (95% CI: 4.8%, 5.4%) and 3.8% (95% CI: 3.6%, 4.0%) for NH Asian/Pacific Islander male and females; and 6.2% (95% CI: 6.1%, 6.4%) and 4.3% (95% CI: 4.2, 4.5%) for Hispanic male and females.[4]

Under status quo, our model projected cumulative incidence of KF requiring KRT to be: 2.3% (95% UI: 0.4%, 5.2%) for non-Hispanic white, 6.2% (95% UI: 2.8%, 10.6%) for non-Hispanic Black 35-year-olds, 3.6% (95% UI: 1.1%, 6.7%) for Hispanic, and 3.3% (95% UI: 1.2%, 6.5%) for additional racial and ethnic group 35-year-olds. Our model closely replicated results in this previous study.

© 2025 Cusick MM et al. JAMA Network Open.

eTable 1. Effective Sample Size by Racial and Ethnic Group

Age	non-Hispanic Black	Non-Hispanic white	Hispanic	Additional groups
30-39	404	401	516	53
40-49	592	211	417	222
50-59	412	749	568	124
60-69	790	729	642	167
70-79	2268	2642	661	392

eTable 2. eGFR G3a Specific Costs by Racial and Ethnic Group

Race group	Self-reported diabetes prevalence	Overall G3a costs
Hispanic	27.43%	$144
Non-Hispanic Black	27.33%	$143
Non-Hispanic white	23.28%	$127
Additional groups	42.31%	$204

© 2025 Cusick MM et al. JAMA Network Open.

eTable 3. Probabilistic Sensitivity Analysis Input Table

Parameters	Value	Range (95% UI)	Distribution
**Screening parameters**			
UACR screening sensitivity[5]	0.87	(0.81, 0.91)	Multivariate normal
UACR screening specificity[5]	0.88	(0.84. 0.91)	Multivariate normal
Cost of UACR screening[6,7]	$49	($37, $62)	Symmetrical beta
Probability of treatment initiation after diagnosis[8,9]	0.75	(0.5,1)	Symmetrical beta
**Diagnosis parameters**			
Cost of estimated GFR[7,10]	$23	($17, $29)	Symmetrical beta
Cost of retroperitoneal ultrasound[11]	$420	($312, $526)	Symmetrical beta
**Treatment parameters**			
ACE/ARBs – CKD progression reduction – hazards ratio[12–15]	0.81	(0.52, 1)	Lognormal
Monthly cost of ACE/ARBs[7,16]	$34	($25, $43)	Beta
SGLT2 inhibitors – CKD progression reduction – hazards ratio (persons without diabetes)[3,17]	0.51	(0.34, 0.72)	Multivariate lognormal
SGLT2 inhibitors – all-cause mortality reduction – hazards ratio (persons without diabetes)[2,35]	0.54	(0.32, 0.86)	Multivariate lognormal
SGLT2 inhibitors – CKD progression reduction – hazards ratio (persons with diabetes)[3,17]	0.57	(0.45, 0.70)	Multivariate lognormal
SGLT2 inhibitors – all-cause mortality reduction – hazards ratio (persons with diabetes)[3,17]	0.75	(0.56, 0.98)	Multivariate lognormal

© 2025 Cusick MM et al. JAMA Network Open.

Annual discontinuation rate (SGLT2 inhibitors)[17]	0.051	(0.027, 0.083)	Beta
Monthly cost of SGLT2 inhibitors[7,18]	$180	($134, $234)	Symmetrical beta
Disutility associated with medication related angioedema adverse event[19]	0.01	(0.0027, 0.02)	Beta
Cost increase from angioedema medication-related adverse event[20]	$3,876	($2,887, $4,868)	Symmetrical beta
Proportion of diagnosed persons who experience an angioedema medication-related serious adverse event[21]	0.1%	(0.01%, 1.0%)	Beta
Disutility associated with genital infection adverse event[22]	0.001	(0.0002, 0.006)	Beta
Cost increase from genital infection adverse event[23]	$150	($111, $189)	Symmetrical beta
Annual rate of genital infection adverse event[24]	0.037	(0.027, 0.051)	Beta
Disutility associated with euglycemic diabetic ketoacidosis adverse event[25]	0.0098	(0.005, 0.016)	Beta
Cost increase from euglycemic diabetic ketoacidosis adverse event[26]	$30,597	($22,815, $38,423)	Symmetrical beta
Annual rate of euglycemic diabetic ketoacidosis adverse event[27]	0.002	(0.0002, 0.006)	Beta
**Age-specific diabetes prevalence (among those eligible for SGLT2 inhibitor treatment)**[28]			
Hispanic			
Diabetes prevalence (30-39-year-olds)	7.8%	(0%, 33.3%)	Binomial
Diabetes prevalence (40-49-year-olds)	45.9%	(18.2%, 72.7%)	Binomial
Diabetes prevalence (50-59-year-olds)	49.1%	(27.3%, 68.2%)	Binomial
Diabetes prevalence (60-69-year-olds)	50.6%	(38.4%, 61.6%)	Binomial

© 2025 Cusick MM et al. JAMA Network Open.

Diabetes prevalence (70-79-year-olds)	51.2%	(41.7%, 60.0%)	Binomial
Non-Hispanic Black			
Diabetes prevalence (30-39-year-olds)	22.5%	(0%, 50.0%)	Binomial
Diabetes prevalence (40-49-year-olds)	33.6%	(15.4%, 53.8%)	Binomial
Diabetes prevalence (50-59-year-olds)	31.9%	(21.5%, 43.1%)	Binomial
Diabetes prevalence (60-69-year-olds)	41.3%	(32.5%, 50.9%)	Binomial
Diabetes prevalence (70-79-year-olds)	51.3%	(41.9%, 61.0%)	Binomial
Non-Hispanic white			
Diabetes prevalence (30-39-year-olds)	6.4%	(0%, 21.4%)	Binomial
Diabetes prevalence (40-49-year-olds)	25.9%	(5.6%, 44.4%)	Binomial
Diabetes prevalence (50-59-year-olds)	50.9%	(34.3%, 68.6%)	Binomial
Diabetes prevalence (60-69-year-olds)	42.9%	(33.0%, 53.2%)	Binomial
Diabetes prevalence (70-79-year-olds)	34.2%	(28.9%, 39.4%)	Binomial
Additional groups			
Diabetes prevalence (30-39-year-olds)	8.0%	(0%, 40.0%)	Binomial
Diabetes prevalence (40-49-year-olds)	51.7%	(22.2%, 44.1%)	Binomial
Diabetes prevalence (50-59-year-olds)	44.1%	(24.0%, 64.0%)	Binomial
Diabetes prevalence (60-69-year-olds)	51.9%	(38.3%, 66.0%)	Binomial
Diabetes prevalence (70-79-year-olds)	39.4%	(27.1%, 52.5%)	Binomial
**CKD mortality parameters**			

© 2025 Cusick MM et al. JAMA Network Open.

Mortality risk - CKD stage G3a - hazard ratio[29]	1.2	(1.1, 1.3)	Symmetrical beta
Mortality risk - CKD stage G3b - hazard ratio[29]	1.8	(1.7, 1.9)	Symmetrical beta
Mortality risk - CKD stage G4 - hazard ratio [29]	3.2	(3.0, 3.4)	Symmetrical beta
Mortality risk - kidney failure not requiring KRT - hazard ratio[29]	3.2	(3.0, 3.4)	Symmetrical beta
Mortality risk - kidney failure not requiring KRT - hazard ratio[29]	5.9	(5.4, 6.4)	Symmetrical beta
**CKD Quality-of-Life adjustments for health states parameters**			
Quality of life adjustment - CKD Stage G2[3,30]	0.85	(0.7, 0.96)	Beta with induced correlations
Quality of life adjustment - CKD Stage G3a[3,30]	0.81	(0.66, 0.92)	Beta with induced correlations
Quality of life adjustment - CKD Stage G3b[3,30]	0.81	(0.66, 0.92)	Beta with induced correlations
Quality of life adjustment - CKD Stage G4[3,30]	0.74	(0.61, 0.85)	Beta with induced correlations
Quality of life adjustment - kidney failure not requiring KRT[3,30]	0.74	(0.61, 0.85)	Beta with induced correlations
Quality of life adjustment – kidney failure requiring KRT[3,30]	0.60	(0.51, 0.68)	Beta with induced correlations
**CKD stage-specific cost parameters**			
Monthly added cost of CKD Stage G3a (non-diabetic CKD)[7,31]	$32	($24, $41)	Beta with induced correlations
Monthly added cost of CKD Stage G3b (non-diabetic CKD)[7,31]	$108	($80, $140)	Beta with induced correlations
Monthly added cost of CKD Stage G4 (non-diabetic CKD) population[7,31]	$463	($346, $598)	Beta with induced correlations

© 2025 Cusick MM et al. JAMA Network Open.

Monthly added cost of kidney failure not requiring KRT (non-diabetic CKD) population[7,31]	$463	($346, $598)	Beta with induced correlations
Monthly added cost of CKD Stage G3a (diabetic CKD)[7,31]	$438	($243, $688)	Beta with induced correlations
Monthly added cost of CKD Stage G3b (diabetic CKD)[7,31]	$806	($445, $806)	Beta with induced correlations
Monthly added cost of CKD Stage G4 (diabetic CKD) population[7,31]	$1,824	($1022, $2953)	Beta with induced correlations
Monthly added cost of CKD Stage G4 (diabetic CKD) population[7,31]	$1,824	($1022, $2953)	Beta with induced correlations
Monthly added cost of kidney failure not requiring KRT (diabetic CKD) population[7,31]	$1,824	($1022, $2953)	Beta with induced correlations
Monthly added cost of kidney failure requiring KRT[7,31]	$7,020	($5217, $8824)	Beta with induced correlations
Monthly added cost of self-reported diabetes (undetected CKD Stage G3a)[7,31]	$405	($302, $526)	Beta with induced correlations
Monthly added cost of self-reported diabetes (undetected CKD Stage G3b)[7,31]	$684	($509, $887)	Beta with induced correlations
Monthly added cost of self-reported diabetes (undetected CKD Stage G4)[7,31]	$1360	($1009, $1752)	Beta with induced correlations
Monthly added cost of self-reported diabetes (undetected kidney failure not requiring KRT)[7,31]	$1360	($1009, $1752)	Beta with induced correlations
Baseline costs[32]	AHRQ* US expenditure table (2013 converted to 2021)	(75%, 125%)	Symmetrical beta
**CKD stage-specific self-reported diabetes prevalence**[28]			

© 2025 Cusick MM et al. JAMA Network Open.

Hispanic			
Diabetes prevalence (CKD Stage G3a)	27.4%	(19.7%, 35.2%)	Binomial with induced correlations
Diabetes prevalence (CKD Stage G3b)	61.1%	(48.1%, 74.1%)	Binomial with induced correlations
Diabetes prevalence (CKD Stage G4)	55.5%	(34.6%, 73.1%)	Binomial with induced correlations
Non-Hispanic Black			
Diabetes prevalence (CKD Stage G3a)	27.3%	(22.5%, 32.4%)	Binomial with induced correlations
Diabetes prevalence (CKD Stage G3b)	33.2%	(25.0%, 40.9%)	Binomial with induced correlations
Diabetes prevalence (CKD Stage G4)	51.6%	(35.7%, 66.7%)	Binomial with induced correlations
Non-Hispanic white			
Diabetes prevalence (CKD Stage G3a)	23.3%	(19.5%, 27.2%)	Binomial with induced correlations
Diabetes prevalence (CKD Stage G3b)	34.4%	(28.0%, 41.1%)	Binomial with induced correlations
Diabetes prevalence (CKD Stage G4)	32.1%	(19.0%, 47.6%)	Binomial with induced correlations
Additional groups			

© 2025 Cusick MM et al. JAMA Network Open.

Diabetes prevalence (CKD Stage G3a)	42.4%	(31.3%, 53.8%)	Binomial with induced correlations
Diabetes prevalence (CKD Stage G3b)	51.4%	(28.6%, 71.4%)	Binomial with induced correlations
Diabetes prevalence (CKD Stage G4)	64.5%	(30.0%, 90.0%)	Binomial with induced correlations
**Calibration parameters**[1,28]			Calibration

© 2025 Cusick MM et al. JAMA Network Open.

eTable 4. SGLT2 Inhibitor Hazard Ratio Ranges in Sensitivity Analyses

		SGLT2 inhibitor effectiveness hazard ratios				
		40% more effective	20% more effective	Base case	20% less effective	40% less effective
All-cause mortality HR	Diabetic	0.45	0.60	0.75	0.90	1
	Non-diabetic	0.32	0.43	0.54	0.65	0.75
Kidney composite HR	Diabetic	0.34	0.46	0.57	0.68	0.80
	Non-diabetic	0.31	0.41	0.51	0.62	0.72

© 2025 Cusick MM et al. JAMA Network Open.

eTable 5. Concordance Between Calibration Targets and Model Projections

Racial/ethnic group	Overlap between 95% CIs	Model mean in calibration target 95% CI
Hispanic	182 (97%)	160 (85%)
Non-Hispanic Black	181 (96%)	150 (80%)
Non-Hispanic white	184 (98%)	149 (78%)
Additional groups	186 (99%)	174 (93%)

© 2025 Cusick MM et al. JAMA Network Open.

eTable 6. CKD Distribution Estimates and Calibration Targets (NHANES Estimates) and Calibrated Model Projections for Hispanic Adults Aged 40 to 49 Years

	Target	Model
eGFR Stage 1, no albuminuria, not detected	72.62 95% UI:(68.18,76.72)	73.98 95% UI:(69.42,78.42)
eGFR Stage 1, microalbuminuria, not detected	7.7 95% UI:(5.52,10.37)	7.02 95% UI:(5.59,8.69)
eGFR Stage 1, macroalbuminuria, not detected	1.63 95% UI:(0.56,3.72)	0.9 95% UI:(0.5,1.56)
eGFR Stage 2, no albuminuria, not detected	15.05 95% UI:(12.07,18.43)	14.58 95% UI:(10.45,18.78)
eGFR Stage 2, microalbuminuria, not detected	0.89 95% UI:(0.5,1.45)	1.51 95% UI:(0.95,2.22)
eGFR Stage 2, macroalbuminuria, not detected	0.37 95% UI:(0.1,0.94)	0.54 95% UI:(0.27,0.96)
eGFR Stage 3a, no albuminuria, not detected	0.53 95% UI:(0.35,0.74)	0.4 95% UI:(0.1,0.89)
eGFR Stage 3a, microalbuminuria, not detected	0.05 95% UI:(0.02,0.1)	0.09 95% UI:(0.02,0.19)
eGFR Stage 3a, macroalbuminuria, not detected	0.05 95% UI:(0.01,0.14)	0.04 95% UI:(0.01,0.12)
eGFR Stage 3b, no albuminuria, not detected	0.12 95% UI:(0.06,0.21)	0.1 95% UI:(0.04,0.19)
eGFR Stage 3b, microalbuminuria, not detected	0.02 95% UI:(0.01,0.05)	0.03 95% UI:(0.01,0.06)
eGFR Stage 3b, macroalbuminuria, not detected	0.02 95% UI:(0.0,0.06)	0.02 95% UI:(0.01,0.05)
eGFR Stage 4, no albuminuria, not detected	0.02 95% UI:(0.0,0.1)	0.02 95% UI:(0.0,0.04)
eGFR Stage 4, microalbuminuria, not detected	0.01 95% UI:(0.0,0.05)	0.01 95% UI:(0.0,0.02)
eGFR Stage 4, macroalbuminuria, not detected	0.02 95% UI:(0.0,0.05)	0.01 95% UI:(0.0,0.02)
eGFR Stage 1, microalbuminuria, detected	0.15 95% UI:(0.06,0.31)	0.11 95% UI:(0.06,0.21)
eGFR Stage 1, macroalbuminuria, detected	0.09 95% UI:(0.02,0.28)	0.03 95% UI:(0.01,0.08)
eGFR Stage 2, microalbuminuria, detected	0.04 95% UI:(0.01,0.08)	0.02 95% UI:(0.01,0.03)
eGFR Stage 2, macroalbuminuria, detected	0.04 95% UI:(0.01,0.14)	0.02 95% UI:(0.01,0.05)
eGFR Stage 3a, no albuminuria, detected	0.02 95% UI:(0.01,0.06)	0.01 95% UI:(0.0,0.03)
eGFR Stage 3a, microalbuminuria, detected	0.0 95% UI:(0.0,0.01)	0.0 95% UI:(0.0,0.01)
eGFR Stage 3a, macroalbuminuria, detected	0.01 95% UI:(0.0,0.04)	0.0 95% UI:(0.0,0.01)
eGFR Stage 3b, no albuminuria, detected	0.02 95% UI:(0.01,0.05)	0.01 95% UI:(0.0,0.02)
eGFR Stage 3b, microalbuminuria, detected	0.01 95% UI:(0.0,0.02)	0.0 95% UI:(0.0,0.01)
eGFR Stage 3b, macroalbuminuria, detected	0.02 95% UI:(0.0,0.05)	0.0 95% UI:(0.0,0.01)
eGFR Stage 4, no albuminuria, detected	0.01 95% UI:(0.0,0.03)	0.0 95% UI:(0.0,0.01)
eGFR Stage 4, microalbuminuria, detected	0.01 95% UI:(0.0,0.03)	0.0 95% UI:(0.0,0.01)
eGFR Stage 4, macroalbuminuria, detected	0.02 95% UI:(0.0,0.06)	0.01 95% UI:(0.0,0.01)
eGFR Stage 1, microalbuminuria, detected and treated	0.09 95% UI:(0.03,0.2)	0.23 95% UI:(0.15,0.33)
eGFR Stage 1, macroalbuminuria, detected and treated	0.09 95% UI:(0.02,0.28)	0.06 95% UI:(0.03,0.11)
eGFR Stage 2, microalbuminuria, detected and treated	0.02 95% UI:(0.01,0.05)	0.03 95% UI:(0.01,0.05)
eGFR Stage 2, macroalbuminuria, detected and treated	0.04 95% UI:(0.01,0.12)	0.02 95% UI:(0.01,0.06)
eGFR Stage 3a, no albuminuria, detected and treated	0.02 95% UI:(0.01,0.06)	0.03 95% UI:(0.01,0.08)
eGFR Stage 3a, microalbuminuria, detected and treated	0.01 95% UI:(0.0,0.02)	0.01 95% UI:(0.0,0.02)
eGFR Stage 3a, macroalbuminuria, detected and treated	0.02 95% UI:(0.0,0.06)	0.01 95% UI:(0.0,0.02)
eGFR Stage 3b, no albuminuria, detected and treated	0.02 95% UI:(0.01,0.06)	0.02 95% UI:(0.01,0.03)
eGFR Stage 3b, microalbuminuria, detected and treated	0.01 95% UI:(0.0,0.02)	0.01 95% UI:(0.0,0.01)
eGFR Stage 3b, macroalbuminuria, detected and treated	0.03 95% UI:(0.01,0.09)	0.01 95% UI:(0.0,0.01)
eGFR Stage 4, no albuminuria, detected and treated	0.01 95% UI:(0.0,0.03)	0.01 95% UI:(0.0,0.03)
eGFR Stage 4, microalbuminuria, detected and treated	0.01 95% UI:(0.0,0.03)	0.01 95% UI:(0.0,0.01)
eGFR Stage 4, macroalbuminuria, detected and treated	0.04 95% UI:(0.0,0.09)	0.01 95% UI:(0.0,0.02)
Kidney failure pre-kidney replacement therapy, no albuminuria	0.0 95% UI:(0.0,0.0)	0.0 95% UI:(0.0,0.01)
Kidney failure pre-kidney replacement therapy, microalbuminuria	0.0 95% UI:(0.0,0.02)	0.0 95% UI:(0.0,0.01)
Kidney failure pre-kidney replacement therapy, macroalbuminuria	0.0 95% UI:(0.0,0.01)	0.01 95% UI:(0.0,0.02)
Kidney failure on kidney replacement therapy, no albuminuria	0.0 95% UI:(0.0,0.0)	0.0 95% UI:(0.0,0.01)
Kidney failure on kidney replacement therapy, microalbuminuria	0.01 95% UI:(0.0,0.05)	0.0 95% UI:(0.0,0.01)
Kidney failure on kidney replacement therapy, macroalbuminuria	0.03 95% UI:(0.0,0.08)	0.05 95% UI:(0.02,0.1)

© 2025 Cusick MM et al. JAMA Network Open.

eTable 7. CKD Distribution Estimates and Calibration Targets (NHANES Estimates) and Calibrated Model Projections for Hispanic Adults Aged 50 to 59 Years

	Target	Model
eGFR Stage 1, no albuminuria, not detected	61.12 95% UI:(57.03,65.05)	61.93 95% UI:(54.99,68.44)
eGFR Stage 1, microalbuminuria, not detected	7.18 95% UI:(5.26,9.5)	7.8 95% UI:(6.04,9.57)
eGFR Stage 1, macroalbuminuria, not detected	0.74 95% UI:(0.23,1.81)	0.74 95% UI:(0.39,1.24)
eGFR Stage 2, no albuminuria, not detected	26.05 95% UI:(22.77,29.5)	22.3 95% UI:(16.37,28.59)
eGFR Stage 2, microalbuminuria, not detected	1.67 95% UI:(1.01,2.58)	3.29 95% UI:(2.17,4.67)
eGFR Stage 2, macroalbuminuria, not detected	0.38 95% UI:(0.08,1.14)	0.92 95% UI:(0.52,1.5)
eGFR Stage 3a, no albuminuria, not detected	1.14 95% UI:(0.8,1.56)	0.78 95% UI:(0.2,1.79)
eGFR Stage 3a, microalbuminuria, not detected	0.13 95% UI:(0.05,0.29)	0.23 95% UI:(0.06,0.55)
eGFR Stage 3a, macroalbuminuria, not detected	0.06 95% UI:(0.02,0.14)	0.1 95% UI:(0.02,0.28)
eGFR Stage 3b, no albuminuria, not detected	0.29 95% UI:(0.16,0.45)	0.2 95% UI:(0.05,0.46)
eGFR Stage 3b, microalbuminuria, not detected	0.06 95% UI:(0.02,0.13)	0.09 95% UI:(0.03,0.2)
eGFR Stage 3b, macroalbuminuria, not detected	0.03 95% UI:(0.0,0.08)	0.06 95% UI:(0.02,0.15)
eGFR Stage 4, no albuminuria, not detected	0.06 95% UI:(0.0,0.21)	0.04 95% UI:(0.0,0.1)
eGFR Stage 4, microalbuminuria, not detected	0.04 95% UI:(0.0,0.15)	0.02 95% UI:(0.0,0.06)
eGFR Stage 4, macroalbuminuria, not detected	0.03 95% UI:(0.0,0.09)	0.03 95% UI:(0.01,0.07)
eGFR Stage 1, microalbuminuria, detected	0.09 95% UI:(0.03,0.2)	0.2 95% UI:(0.11,0.34)
eGFR Stage 1, macroalbuminuria, detected	0.03 95% UI:(0.01,0.11)	0.04 95% UI:(0.01,0.08)
eGFR Stage 2, microalbuminuria, detected	0.05 95% UI:(0.02,0.11)	0.05 95% UI:(0.02,0.09)
eGFR Stage 2, macroalbuminuria, detected	0.03 95% UI:(0.0,0.1)	0.04 95% UI:(0.01,0.08)
eGFR Stage 3a, no albuminuria, detected	0.04 95% UI:(0.01,0.1)	0.03 95% UI:(0.01,0.07)
eGFR Stage 3a, microalbuminuria, detected	0.01 95% UI:(0.0,0.03)	0.01 95% UI:(0.0,0.03)
eGFR Stage 3a, macroalbuminuria, detected	0.01 95% UI:(0.0,0.03)	0.01 95% UI:(0.0,0.02)
eGFR Stage 3b, no albuminuria, detected	0.04 95% UI:(0.01,0.1)	0.02 95% UI:(0.01,0.03)
eGFR Stage 3b, microalbuminuria, detected	0.02 95% UI:(0.0,0.05)	0.01 95% UI:(0.0,0.02)
eGFR Stage 3b, macroalbuminuria, detected	0.02 95% UI:(0.0,0.06)	0.01 95% UI:(0.0,0.02)
eGFR Stage 4, no albuminuria, detected	0.01 95% UI:(0.0,0.05)	0.01 95% UI:(0.0,0.03)
eGFR Stage 4, microalbuminuria, detected	0.01 95% UI:(0.0,0.05)	0.01 95% UI:(0.0,0.02)
eGFR Stage 4, macroalbuminuria, detected	0.03 95% UI:(0.0,0.1)	0.01 95% UI:(0.0,0.02)
eGFR Stage 1, microalbuminuria, detected and treated	0.1 95% UI:(0.03,0.21)	0.41 95% UI:(0.26,0.57)
eGFR Stage 1, macroalbuminuria, detected and treated	0.04 95% UI:(0.01,0.12)	0.07 95% UI:(0.03,0.12)
eGFR Stage 2, microalbuminuria, detected and treated	0.04 95% UI:(0.01,0.08)	0.08 95% UI:(0.04,0.13)
eGFR Stage 2, macroalbuminuria, detected and treated	0.04 95% UI:(0.01,0.14)	0.05 95% UI:(0.02,0.1)
eGFR Stage 3a, no albuminuria, detected and treated	0.05 95% UI:(0.01,0.13)	0.08 95% UI:(0.02,0.21)
eGFR Stage 3a, microalbuminuria, detected and treated	0.01 95% UI:(0.0,0.04)	0.03 95% UI:(0.01,0.09)
eGFR Stage 3a, macroalbuminuria, detected and treated	0.02 95% UI:(0.0,0.06)	0.02 95% UI:(0.0,0.05)
eGFR Stage 3b, no albuminuria, detected and treated	0.05 95% UI:(0.01,0.12)	0.04 95% UI:(0.01,0.08)
eGFR Stage 3b, microalbuminuria, detected and treated	0.03 95% UI:(0.01,0.06)	0.02 95% UI:(0.01,0.04)
eGFR Stage 3b, macroalbuminuria, detected and treated	0.04 95% UI:(0.01,0.12)	0.01 95% UI:(0.0,0.04)
eGFR Stage 4, no albuminuria, detected and treated	0.01 95% UI:(0.0,0.06)	0.03 95% UI:(0.01,0.07)
eGFR Stage 4, microalbuminuria, detected and treated	0.02 95% UI:(0.0,0.09)	0.02 95% UI:(0.01,0.04)
eGFR Stage 4, macroalbuminuria, detected and treated	0.06 95% UI:(0.0,0.17)	0.02 95% UI:(0.01,0.05)
Kidney failure pre-kidney replacement therapy, no albuminuria	0.0 95% UI:(0.0,0.0)	0.01 95% UI:(0.0,0.02)
Kidney failure pre-kidney replacement therapy, microalbuminuria	0.01 95% UI:(0.0,0.05)	0.01 95% UI:(0.0,0.02)
Kidney failure pre-kidney replacement therapy, macroalbuminuria	0.01 95% UI:(0.0,0.03)	0.01 95% UI:(0.0,0.04)
Kidney failure on kidney replacement therapy, no albuminuria	0.0 95% UI:(0.0,0.0)	0.01 95% UI:(0.0,0.02)
Kidney failure on kidney replacement therapy, microalbuminuria	0.03 95% UI:(0.0,0.11)	0.01 95% UI:(0.0,0.03)
Kidney failure on kidney replacement therapy, macroalbuminuria	0.06 95% UI:(0.0,0.15)	0.12 95% UI:(0.03,0.26)

© 2025 Cusick MM et al. JAMA Network Open.

eTable 8. CKD Distribution Estimates and Calibration Targets (NHANES Estimates) and Calibrated Model Projections for Hispanic Adults Aged 60 to 69 Years

	Target	Model
eGFR Stage 1, no albuminuria, not detected	37.9 95% UI:(34.15,41.65)	38.02 95% UI:(32.02,44.16)
eGFR Stage 1, microalbuminuria, not detected	6.57 95% UI:(4.63,8.93)	5.46 95% UI:(3.85,6.99)
eGFR Stage 1, macroalbuminuria, not detected	0.94 95% UI:(0.43,1.79)	0.27 95% UI:(0.11,0.48)
eGFR Stage 2, no albuminuria, not detected	42.44 95% UI:(39.0,45.9)	38.97 95% UI:(33.41,44.56)
eGFR Stage 2, microalbuminuria, not detected	3.98 95% UI:(2.97,5.2)	7.14 95% UI:(5.28,9.01)
eGFR Stage 2, macroalbuminuria, not detected	1.11 95% UI:(0.59,1.91)	1.26 95% UI:(0.75,1.85)
eGFR Stage 3a, no albuminuria, not detected	2.55 95% UI:(1.81,3.38)	3.25 95% UI:(2.03,4.74)
eGFR Stage 3a, microalbuminuria, not detected	0.4 95% UI:(0.19,0.74)	1.0 95% UI:(0.59,1.55)
eGFR Stage 3a, macroalbuminuria, not detected	0.24 95% UI:(0.09,0.51)	0.36 95% UI:(0.2,0.6)
eGFR Stage 3b, no albuminuria, not detected	0.6 95% UI:(0.31,0.95)	0.71 95% UI:(0.36,1.2)
eGFR Stage 3b, microalbuminuria, not detected	0.17 95% UI:(0.08,0.31)	0.32 95% UI:(0.16,0.57)
eGFR Stage 3b, macroalbuminuria, not detected	0.11 95% UI:(0.03,0.26)	0.23 95% UI:(0.13,0.4)
eGFR Stage 4, no albuminuria, not detected	0.11 95% UI:(0.0,0.5)	0.12 95% UI:(0.04,0.24)
eGFR Stage 4, microalbuminuria, not detected	0.07 95% UI:(0.0,0.29)	0.07 95% UI:(0.02,0.16)
eGFR Stage 4, macroalbuminuria, not detected	0.09 95% UI:(0.01,0.24)	0.12 95% UI:(0.05,0.25)
eGFR Stage 1, microalbuminuria, detected	0.06 95% UI:(0.02,0.15)	0.18 95% UI:(0.09,0.32)
eGFR Stage 1, macroalbuminuria, detected	0.03 95% UI:(0.0,0.08)	0.02 95% UI:(0.0,0.03)
eGFR Stage 2, microalbuminuria, detected	0.08 95% UI:(0.03,0.16)	0.16 95% UI:(0.09,0.28)
eGFR Stage 2, macroalbuminuria, detected	0.07 95% UI:(0.02,0.17)	0.06 95% UI:(0.03,0.12)
eGFR Stage 3a, no albuminuria, detected	0.05 95% UI:(0.01,0.13)	0.05 95% UI:(0.02,0.13)
eGFR Stage 3a, microalbuminuria, detected	0.01 95% UI:(0.0,0.04)	0.04 95% UI:(0.02,0.09)
eGFR Stage 3a, macroalbuminuria, detected	0.02 95% UI:(0.0,0.06)	0.02 95% UI:(0.01,0.04)
eGFR Stage 3b, no albuminuria, detected	0.05 95% UI:(0.01,0.13)	0.03 95% UI:(0.01,0.08)
eGFR Stage 3b, microalbuminuria, detected	0.02 95% UI:(0.0,0.06)	0.02 95% UI:(0.01,0.05)
eGFR Stage 3b, macroalbuminuria, detected	0.03 95% UI:(0.0,0.11)	0.02 95% UI:(0.01,0.04)
eGFR Stage 4, no albuminuria, detected	0.01 95% UI:(0.0,0.07)	0.02 95% UI:(0.01,0.05)
eGFR Stage 4, microalbuminuria, detected	0.01 95% UI:(0.0,0.07)	0.02 95% UI:(0.01,0.04)
eGFR Stage 4, macroalbuminuria, detected	0.04 95% UI:(0.0,0.13)	0.02 95% UI:(0.01,0.06)
eGFR Stage 1, microalbuminuria, detected and treated	0.16 95% UI:(0.06,0.32)	0.41 95% UI:(0.25,0.59)
eGFR Stage 1, macroalbuminuria, detected and treated	0.07 95% UI:(0.02,0.19)	0.03 95% UI:(0.01,0.07)
eGFR Stage 2, microalbuminuria, detected and treated	0.19 95% UI:(0.09,0.33)	0.27 95% UI:(0.17,0.41)
eGFR Stage 2, macroalbuminuria, detected and treated	0.15 95% UI:(0.05,0.33)	0.09 95% UI:(0.05,0.16)
eGFR Stage 3a, no albuminuria, detected and treated	0.21 95% UI:(0.07,0.48)	0.24 95% UI:(0.1,0.48)
eGFR Stage 3a, microalbuminuria, detected and treated	0.08 95% UI:(0.02,0.2)	0.12 95% UI:(0.05,0.24)
eGFR Stage 3a, macroalbuminuria, detected and treated	0.16 95% UI:(0.05,0.35)	0.05 95% UI:(0.02,0.11)
eGFR Stage 3b, no albuminuria, detected and treated	0.17 95% UI:(0.06,0.35)	0.1 95% UI:(0.04,0.21)
eGFR Stage 3b, microalbuminuria, detected and treated	0.13 95% UI:(0.05,0.24)	0.06 95% UI:(0.02,0.13)
eGFR Stage 3b, macroalbuminuria, detected and treated	0.23 95% UI:(0.07,0.49)	0.05 95% UI:(0.02,0.1)
eGFR Stage 4, no albuminuria, detected and treated	0.04 95% UI:(0.0,0.2)	0.07 95% UI:(0.02,0.17)
eGFR Stage 4, microalbuminuria, detected and treated	0.07 95% UI:(0.0,0.28)	0.06 95% UI:(0.02,0.12)
eGFR Stage 4, macroalbuminuria, detected and treated	0.29 95% UI:(0.03,0.63)	0.08 95% UI:(0.03,0.18)
Kidney failure pre-kidney replacement therapy, no albuminuria	0.0 95% UI:(0.0,0.0)	0.02 95% UI:(0.0,0.05)
Kidney failure pre-kidney replacement therapy, microalbuminuria	0.02 95% UI:(0.0,0.12)	0.02 95% UI:(0.0,0.05)
Kidney failure pre-kidney replacement therapy, macroalbuminuria	0.02 95% UI:(0.0,0.08)	0.04 95% UI:(0.01,0.11)
Kidney failure on kidney replacement therapy, no albuminuria	0.0 95% UI:(0.0,0.0)	0.01 95% UI:(0.0,0.05)
Kidney failure on kidney replacement therapy, microalbuminuria	0.07 95% UI:(0.0,0.26)	0.02 95% UI:(0.0,0.08)
Kidney failure on kidney replacement therapy, macroalbuminuria	0.17 95% UI:(0.02,0.41)	0.26 95% UI:(0.05,0.64)

© 2025 Cusick MM et al. JAMA Network Open.

eTable 9. CKD Distribution Estimates and Calibration Targets (NHANES Estimates) and Calibrated Model Projections for Hispanic Adults Aged 70 to 79 Years

	Target	Model
eGFR Stage 1, no albuminuria, not detected	8.24 95% UI:(6.29,10.47)	11.82 95% UI:(7.37,18.01)
eGFR Stage 1, microalbuminuria, not detected	3.78 95% UI:(2.51,5.35)	1.66 95% UI:(0.76,2.85)
eGFR Stage 1, macroalbuminuria, not detected	0.21 95% UI:(0.09,0.43)	0.03 95% UI:(0.01,0.07)
eGFR Stage 2, no albuminuria, not detected	45.54 95% UI:(40.68,50.11)	49.72 95% UI:(41.82,57.05)
eGFR Stage 2, microalbuminuria, not detected	11.22 95% UI:(7.96,15.11)	9.88 95% UI:(7.28,12.34)
eGFR Stage 2, macroalbuminuria, not detected	1.23 95% UI:(0.57,2.27)	0.84 95% UI:(0.36,1.44)
eGFR Stage 3a, no albuminuria, not detected	8.43 95% UI:(6.0,11.05)	9.61 95% UI:(6.06,13.75)
eGFR Stage 3a, microalbuminuria, not detected	3.49 95% UI:(1.87,5.63)	3.23 95% UI:(2.02,4.72)
eGFR Stage 3a, macroalbuminuria, not detected	0.83 95% UI:(0.37,1.55)	0.63 95% UI:(0.31,1.12)
eGFR Stage 3b, no albuminuria, not detected	2.11 95% UI:(1.09,3.49)	2.99 95% UI:(1.72,4.54)
eGFR Stage 3b, microalbuminuria, not detected	1.69 95% UI:(0.86,2.82)	1.42 95% UI:(0.86,2.09)
eGFR Stage 3b, macroalbuminuria, not detected	0.38 95% UI:(0.1,0.92)	0.58 95% UI:(0.33,0.91)
eGFR Stage 4, no albuminuria, not detected	0.5 95% UI:(0.0,2.44)	0.64 95% UI:(0.22,1.24)
eGFR Stage 4, microalbuminuria, not detected	0.65 95% UI:(0.02,2.07)	0.41 95% UI:(0.14,0.84)
eGFR Stage 4, macroalbuminuria, not detected	0.4 95% UI:(0.03,1.26)	0.44 95% UI:(0.2,0.82)
eGFR Stage 1, microalbuminuria, detected	0.04 95% UI:(0.01,0.1)	0.07 95% UI:(0.03,0.15)
eGFR Stage 1, macroalbuminuria, detected	0.01 95% UI:(0.0,0.02)	0.0 95% UI:(0.0,0.0)
eGFR Stage 2, microalbuminuria, detected	0.27 95% UI:(0.11,0.56)	0.28 95% UI:(0.15,0.49)
eGFR Stage 2, macroalbuminuria, detected	0.11 95% UI:(0.02,0.31)	0.04 95% UI:(0.02,0.09)
eGFR Stage 3a, no albuminuria, detected	0.3 95% UI:(0.07,0.74)	0.16 95% UI:(0.07,0.36)
eGFR Stage 3a, microalbuminuria, detected	0.15 95% UI:(0.03,0.4)	0.14 95% UI:(0.06,0.26)
eGFR Stage 3a, macroalbuminuria, detected	0.11 95% UI:(0.03,0.28)	0.04 95% UI:(0.02,0.09)
eGFR Stage 3b, no albuminuria, detected	0.18 95% UI:(0.05,0.42)	0.11 95% UI:(0.05,0.24)
eGFR Stage 3b, microalbuminuria, detected	0.41 95% UI:(0.13,0.9)	0.09 95% UI:(0.04,0.17)
eGFR Stage 3b, macroalbuminuria, detected	0.21 95% UI:(0.05,0.54)	0.05 95% UI:(0.02,0.09)
eGFR Stage 4, no albuminuria, detected	0.06 95% UI:(0.0,0.36)	0.08 95% UI:(0.03,0.18)
eGFR Stage 4, microalbuminuria, detected	0.21 95% UI:(0.01,0.82)	0.07 95% UI:(0.03,0.16)
eGFR Stage 4, macroalbuminuria, detected	0.31 95% UI:(0.02,1.03)	0.09 95% UI:(0.03,0.21)
eGFR Stage 1, microalbuminuria, detected and treated	0.1 95% UI:(0.04,0.21)	0.18 95% UI:(0.07,0.34)
eGFR Stage 1, macroalbuminuria, detected and treated	0.02 95% UI:(0.01,0.06)	0.0 95% UI:(0.0,0.01)
eGFR Stage 2, microalbuminuria, detected and treated	0.58 95% UI:(0.28,1.04)	0.49 95% UI:(0.29,0.71)
eGFR Stage 2, macroalbuminuria, detected and treated	0.21 95% UI:(0.06,0.54)	0.08 95% UI:(0.03,0.14)
eGFR Stage 3a, no albuminuria, detected and treated	0.57 95% UI:(0.2,1.22)	0.8 95% UI:(0.4,1.39)
eGFR Stage 3a, microalbuminuria, detected and treated	0.66 95% UI:(0.23,1.42)	0.43 95% UI:(0.22,0.73)
eGFR Stage 3a, macroalbuminuria, detected and treated	0.5 95% UI:(0.2,1.0)	0.11 95% UI:(0.04,0.23)
eGFR Stage 3b, no albuminuria, detected and treated	0.75 95% UI:(0.32,1.41)	0.41 95% UI:(0.2,0.71)
eGFR Stage 3b, microalbuminuria, detected and treated	1.08 95% UI:(0.49,1.94)	0.26 95% UI:(0.13,0.43)
eGFR Stage 3b, macroalbuminuria, detected and treated	0.86 95% UI:(0.32,1.74)	0.13 95% UI:(0.06,0.23)
eGFR Stage 4, no albuminuria, detected and treated	0.24 95% UI:(0.0,1.25)	0.35 95% UI:(0.12,0.7)
eGFR Stage 4, microalbuminuria, detected and treated	0.57 95% UI:(0.02,1.83)	0.26 95% UI:(0.1,0.52)
eGFR Stage 4, macroalbuminuria, detected and treated	1.18 95% UI:(0.1,2.85)	0.29 95% UI:(0.11,0.66)
Kidney failure pre-kidney replacement therapy, no albuminuria	0.0 95% UI:(0.0,0.0)	0.07 95% UI:(0.01,0.19)
Kidney failure pre-kidney replacement therapy, microalbuminuria	0.17 95% UI:(0.0,0.77)	0.08 95% UI:(0.01,0.2)
Kidney failure pre-kidney replacement therapy, macroalbuminuria	0.07 95% UI:(0.0,0.35)	0.15 95% UI:(0.04,0.35)
Kidney failure on kidney replacement therapy, no albuminuria	0.0 95% UI:(0.0,0.0)	0.04 95% UI:(0.0,0.13)
Kidney failure on kidney replacement therapy, microalbuminuria	0.61 95% UI:(0.02,1.81)	0.07 95% UI:(0.01,0.21)
Kidney failure on kidney replacement therapy, macroalbuminuria	0.78 95% UI:(0.04,2.09)	0.69 95% UI:(0.17,1.5)

© 2025 Cusick MM et al. JAMA Network Open.

eTable 10. CKD Distribution Estimates and Calibration Targets (NHANES Estimates) and Calibrated Model Projections for Non-Hispanic Black Adults Aged 40 to 49 Years

	Target	Model
eGFR Stage 1, no albuminuria, not detected	46.92 95% UI:(42.91,50.92)	45.19 95% UI:(39.8,52.5)
eGFR Stage 1, microalbuminuria, not detected	3.3 95% UI:(2.01,5.08)	4.77 95% UI:(3.37,6.68)
eGFR Stage 1, macroalbuminuria, not detected	0.93 95% UI:(0.29,2.23)	0.26 95% UI:(0.11,0.51)
eGFR Stage 2, no albuminuria, not detected	39.55 95% UI:(36.28,42.79)	38.71 95% UI:(31.61,43.85)
eGFR Stage 2, microalbuminuria, not detected	2.52 95% UI:(1.55,3.85)	4.73 95% UI:(3.24,6.69)
eGFR Stage 2, macroalbuminuria, not detected	0.99 95% UI:(0.39,2.08)	0.65 95% UI:(0.33,1.24)
eGFR Stage 3a, no albuminuria, not detected	3.08 95% UI:(2.41,3.83)	2.07 95% UI:(0.77,4.13)
eGFR Stage 3a, microalbuminuria, not detected	0.29 95% UI:(0.15,0.5)	0.53 95% UI:(0.2,1.07)
eGFR Stage 3a, macroalbuminuria, not detected	0.21 95% UI:(0.06,0.54)	0.11 95% UI:(0.03,0.27)
eGFR Stage 3b, no albuminuria, not detected	0.46 95% UI:(0.27,0.69)	0.64 95% UI:(0.33,1.09)
eGFR Stage 3b, microalbuminuria, not detected	0.04 95% UI:(0.02,0.09)	0.23 95% UI:(0.12,0.4)
eGFR Stage 3b, macroalbuminuria, not detected	0.12 95% UI:(0.04,0.26)	0.08 95% UI:(0.03,0.17)
eGFR Stage 4, no albuminuria, not detected	0.05 95% UI:(0.01,0.13)	0.1 95% UI:(0.02,0.21)
eGFR Stage 4, microalbuminuria, not detected	0.01 95% UI:(0.0,0.03)	0.05 95% UI:(0.01,0.11)
eGFR Stage 4, macroalbuminuria, not detected	0.05 95% UI:(0.01,0.12)	0.04 95% UI:(0.01,0.07)
eGFR Stage 1, microalbuminuria, detected	0.03 95% UI:(0.0,0.08)	0.05 95% UI:(0.02,0.12)
eGFR Stage 1, macroalbuminuria, detected	0.0 95% UI:(0.0,0.01)	0.0 95% UI:(0.0,0.01)
eGFR Stage 2, microalbuminuria, detected	0.04 95% UI:(0.01,0.11)	0.07 95% UI:(0.03,0.15)
eGFR Stage 2, macroalbuminuria, detected	0.01 95% UI:(0.0,0.03)	0.01 95% UI:(0.0,0.02)
eGFR Stage 3a, no albuminuria, detected	0.09 95% UI:(0.02,0.24)	0.09 95% UI:(0.03,0.17)
eGFR Stage 3a, microalbuminuria, detected	0.02 95% UI:(0.0,0.06)	0.04 95% UI:(0.01,0.07)
eGFR Stage 3a, macroalbuminuria, detected	0.01 95% UI:(0.0,0.02)	0.01 95% UI:(0.0,0.02)
eGFR Stage 3b, no albuminuria, detected	0.07 95% UI:(0.01,0.19)	0.08 95% UI:(0.03,0.15)
eGFR Stage 3b, microalbuminuria, detected	0.01 95% UI:(0.0,0.04)	0.03 95% UI:(0.01,0.06)
eGFR Stage 3b, macroalbuminuria, detected	0.02 95% UI:(0.0,0.07)	0.01 95% UI:(0.0,0.02)
eGFR Stage 4, no albuminuria, detected	0.02 95% UI:(0.0,0.08)	0.05 95% UI:(0.02,0.09)
eGFR Stage 4, microalbuminuria, detected	0.01 95% UI:(0.0,0.03)	0.03 95% UI:(0.01,0.05)
eGFR Stage 4, macroalbuminuria, detected	0.02 95% UI:(0.0,0.07)	0.02 95% UI:(0.01,0.03)
eGFR Stage 1, microalbuminuria, detected and treated	0.04 95% UI:(0.01,0.12)	0.1 95% UI:(0.03,0.19)
eGFR Stage 1, macroalbuminuria, detected and treated	0.01 95% UI:(0.0,0.03)	0.01 95% UI:(0.0,0.01)
eGFR Stage 2, microalbuminuria, detected and treated	0.06 95% UI:(0.02,0.15)	0.12 95% UI:(0.05,0.22)
eGFR Stage 2, macroalbuminuria, detected and treated	0.02 95% UI:(0.0,0.05)	0.02 95% UI:(0.01,0.04)
eGFR Stage 3a, no albuminuria, detected and treated	0.23 95% UI:(0.08,0.49)	0.24 95% UI:(0.12,0.43)
eGFR Stage 3a, microalbuminuria, detected and treated	0.05 95% UI:(0.02,0.13)	0.09 95% UI:(0.04,0.16)
eGFR Stage 3a, macroalbuminuria, detected and treated	0.03 95% UI:(0.0,0.09)	0.02 95% UI:(0.0,0.04)
eGFR Stage 3b, no albuminuria, detected and treated	0.21 95% UI:(0.09,0.39)	0.15 95% UI:(0.09,0.24)
eGFR Stage 3b, microalbuminuria, detected and treated	0.05 95% UI:(0.02,0.1)	0.07 95% UI:(0.04,0.11)
eGFR Stage 3b, macroalbuminuria, detected and treated	0.09 95% UI:(0.03,0.21)	0.02 95% UI:(0.01,0.05)
eGFR Stage 4, no albuminuria, detected and treated	0.06 95% UI:(0.01,0.14)	0.11 95% UI:(0.05,0.21)
eGFR Stage 4, microalbuminuria, detected and treated	0.03 95% UI:(0.01,0.07)	0.06 95% UI:(0.03,0.12)
eGFR Stage 4, macroalbuminuria, detected and treated	0.08 95% UI:(0.02,0.19)	0.04 95% UI:(0.02,0.07)
Kidney failure pre-kidney replacement therapy, no albuminuria	0.0 95% UI:(0.0,0.0)	0.03 95% UI:(0.0,0.06)
Kidney failure pre-kidney replacement therapy, microalbuminuria	0.01 95% UI:(0.0,0.05)	0.02 95% UI:(0.0,0.05)
Kidney failure pre-kidney replacement therapy, macroalbuminuria	0.03 95% UI:(0.0,0.09)	0.03 95% UI:(0.01,0.06)
Kidney failure on kidney replacement therapy, no albuminuria	0.0 95% UI:(0.0,0.0)	0.02 95% UI:(0.0,0.05)
Kidney failure on kidney replacement therapy, microalbuminuria	0.04 95% UI:(0.0,0.16)	0.03 95% UI:(0.01,0.09)
Kidney failure on kidney replacement therapy, macroalbuminuria	0.09 95% UI:(0.0,0.18)	0.18 95% UI:(0.08,0.34)

© 2025 Cusick MM et al. JAMA Network Open.

eTable 11. CKD Distribution Estimates and Calibration Targets (NHANES Estimates) and Calibrated Model Projections for Non-Hispanic Black Adults Aged 50 to 59 Years

	Target	Model
eGFR Stage 1, no albuminuria, not detected	28.01 95% UI:(23.81,32.46)	31.8 95% UI:(25.94,41.39)
eGFR Stage 1, microalbuminuria, not detected	4.84 95% UI:(3.47,6.52)	4.08 95% UI:(2.63,6.1)
eGFR Stage 1, macroalbuminuria, not detected	0.36 95% UI:(0.13,0.8)	0.18 95% UI:(0.06,0.39)
eGFR Stage 2, no albuminuria, not detected	47.11 95% UI:(43.86,50.17)	45.95 95% UI:(35.74,54.1)
eGFR Stage 2, microalbuminuria, not detected	7.32 95% UI:(5.99,8.8)	7.32 95% UI:(5.02,9.97)
eGFR Stage 2, macroalbuminuria, not detected	0.79 95% UI:(0.31,1.66)	0.9 95% UI:(0.45,1.6)
eGFR Stage 3a, no albuminuria, not detected	5.31 95% UI:(4.14,6.64)	3.14 95% UI:(1.04,6.26)
eGFR Stage 3a, microalbuminuria, not detected	1.22 95% UI:(0.73,1.86)	1.02 95% UI:(0.33,2.14)
eGFR Stage 3a, macroalbuminuria, not detected	0.23 95% UI:(0.07,0.55)	0.23 95% UI:(0.08,0.51)
eGFR Stage 3b, no albuminuria, not detected	0.85 95% UI:(0.5,1.27)	1.01 95% UI:(0.39,2.04)
eGFR Stage 3b, microalbuminuria, not detected	0.18 95% UI:(0.07,0.35)	0.49 95% UI:(0.2,0.95)
eGFR Stage 3b, macroalbuminuria, not detected	0.14 95% UI:(0.05,0.33)	0.19 95% UI:(0.06,0.4)
eGFR Stage 4, no albuminuria, not detected	0.1 95% UI:(0.03,0.22)	0.17 95% UI:(0.04,0.41)
eGFR Stage 4, microalbuminuria, not detected	0.04 95% UI:(0.01,0.11)	0.11 95% UI:(0.03,0.27)
eGFR Stage 4, macroalbuminuria, not detected	0.05 95% UI:(0.01,0.14)	0.09 95% UI:(0.03,0.19)
eGFR Stage 1, microalbuminuria, detected	0.02 95% UI:(0.0,0.07)	0.05 95% UI:(0.01,0.12)
eGFR Stage 1, macroalbuminuria, detected	0.0 95% UI:(0.0,0.0)	0.0 95% UI:(0.0,0.01)
eGFR Stage 2, microalbuminuria, detected	0.06 95% UI:(0.01,0.17)	0.11 95% UI:(0.04,0.21)
eGFR Stage 2, macroalbuminuria, detected	0.0 95% UI:(0.0,0.01)	0.02 95% UI:(0.01,0.04)
eGFR Stage 3a, no albuminuria, detected	0.06 95% UI:(0.01,0.17)	0.11 95% UI:(0.03,0.28)
eGFR Stage 3a, microalbuminuria, detected	0.03 95% UI:(0.0,0.09)	0.06 95% UI:(0.02,0.14)
eGFR Stage 3a, macroalbuminuria, detected	0.0 95% UI:(0.0,0.01)	0.01 95% UI:(0.0,0.04)
eGFR Stage 3b, no albuminuria, detected	0.05 95% UI:(0.01,0.16)	0.1 95% UI:(0.04,0.19)
eGFR Stage 3b, microalbuminuria, detected	0.02 95% UI:(0.0,0.07)	0.06 95% UI:(0.02,0.11)
eGFR Stage 3b, macroalbuminuria, detected	0.01 95% UI:(0.0,0.04)	0.02 95% UI:(0.0,0.05)
eGFR Stage 4, no albuminuria, detected	0.02 95% UI:(0.0,0.05)	0.08 95% UI:(0.03,0.16)
eGFR Stage 4, microalbuminuria, detected	0.01 95% UI:(0.0,0.05)	0.06 95% UI:(0.03,0.11)
eGFR Stage 4, macroalbuminuria, detected	0.01 95% UI:(0.0,0.03)	0.03 95% UI:(0.01,0.06)
eGFR Stage 1, microalbuminuria, detected and treated	0.09 95% UI:(0.02,0.25)	0.11 95% UI:(0.03,0.24)
eGFR Stage 1, macroalbuminuria, detected and treated	0.0 95% UI:(0.0,0.02)	0.01 95% UI:(0.0,0.02)
eGFR Stage 2, microalbuminuria, detected and treated	0.29 95% UI:(0.11,0.62)	0.22 95% UI:(0.09,0.41)
eGFR Stage 2, macroalbuminuria, detected and treated	0.02 95% UI:(0.01,0.06)	0.03 95% UI:(0.01,0.08)
eGFR Stage 3a, no albuminuria, detected and treated	0.59 95% UI:(0.28,1.07)	0.37 95% UI:(0.12,0.78)
eGFR Stage 3a, microalbuminuria, detected and treated	0.33 95% UI:(0.14,0.64)	0.17 95% UI:(0.05,0.37)
eGFR Stage 3a, macroalbuminuria, detected and treated	0.04 95% UI:(0.01,0.11)	0.04 95% UI:(0.01,0.11)
eGFR Stage 3b, no albuminuria, detected and treated	0.53 95% UI:(0.27,0.87)	0.24 95% UI:(0.11,0.42)
eGFR Stage 3b, microalbuminuria, detected and treated	0.3 95% UI:(0.14,0.53)	0.14 95% UI:(0.07,0.25)
eGFR Stage 3b, macroalbuminuria, detected and treated	0.15 95% UI:(0.05,0.34)	0.05 95% UI:(0.01,0.12)
eGFR Stage 4, no albuminuria, detected and treated	0.16 95% UI:(0.06,0.32)	0.22 95% UI:(0.08,0.47)
eGFR Stage 4, microalbuminuria, detected and treated	0.16 95% UI:(0.06,0.33)	0.16 95% UI:(0.07,0.32)
eGFR Stage 4, macroalbuminuria, detected and treated	0.14 95% UI:(0.04,0.31)	0.1 95% UI:(0.04,0.19)
Kidney failure pre-kidney replacement therapy, no albuminuria	0.0 95% UI:(0.0,0.0)	0.05 95% UI:(0.01,0.13)
Kidney failure pre-kidney replacement therapy, microalbuminuria	0.03 95% UI:(0.0,0.1)	0.05 95% UI:(0.01,0.14)
Kidney failure pre-kidney replacement therapy, macroalbuminuria	0.04 95% UI:(0.0,0.14)	0.06 95% UI:(0.01,0.15)
Kidney failure on kidney replacement therapy, no albuminuria	0.0 95% UI:(0.0,0.0)	0.04 95% UI:(0.0,0.13)
Kidney failure on kidney replacement therapy, microalbuminuria	0.13 95% UI:(0.0,0.38)	0.08 95% UI:(0.01,0.24)
Kidney failure on kidney replacement therapy, macroalbuminuria	0.15 95% UI:(0.0,0.37)	0.48 95% UI:(0.16,0.97)

© 2025 Cusick MM et al. JAMA Network Open.

eTable 12. CKD Distribution Estimates and Calibration Targets (NHANES Estimates) and Calibrated Model Projections for Non-Hispanic Black Adults Aged 60 to 69 Years

	Target	Model
eGFR Stage 1, no albuminuria, not detected	12.93 95% UI:(10.72,15.39)	16.05 95% UI:(11.58,22.12)
eGFR Stage 1, microalbuminuria, not detected	2.31 95% UI:(1.64,3.15)	2.13 95% UI:(1.14,3.47)
eGFR Stage 1, macroalbuminuria, not detected	0.28 95% UI:(0.1,0.62)	0.05 95% UI:(0.02,0.13)
eGFR Stage 2, no albuminuria, not detected	49.53 95% UI:(46.48,52.35)	46.89 95% UI:(40.32,53.12)
eGFR Stage 2, microalbuminuria, not detected	7.99 95% UI:(6.2,10.08)	8.36 95% UI:(6.01,10.52)
eGFR Stage 2, macroalbuminuria, not detected	1.37 95% UI:(0.61,2.64)	0.64 95% UI:(0.27,1.13)
eGFR Stage 3a, no albuminuria, not detected	11.54 95% UI:(9.55,13.67)	10.29 95% UI:(7.35,13.36)
eGFR Stage 3a, microalbuminuria, not detected	2.84 95% UI:(1.95,3.93)	3.33 95% UI:(2.24,4.49)
eGFR Stage 3a, macroalbuminuria, not detected	0.85 95% UI:(0.36,1.69)	0.6 95% UI:(0.36,0.94)
eGFR Stage 3b, no albuminuria, not detected	2.48 95% UI:(1.65,3.43)	2.69 95% UI:(1.53,3.94)
eGFR Stage 3b, microalbuminuria, not detected	0.51 95% UI:(0.25,0.89)	1.36 95% UI:(0.76,2.03)
eGFR Stage 3b, macroalbuminuria, not detected	0.76 95% UI:(0.36,1.36)	0.5 95% UI:(0.28,0.8)
eGFR Stage 4, no albuminuria, not detected	0.35 95% UI:(0.1,0.78)	0.42 95% UI:(0.18,0.82)
eGFR Stage 4, microalbuminuria, not detected	0.14 95% UI:(0.04,0.33)	0.31 95% UI:(0.12,0.59)
eGFR Stage 4, macroalbuminuria, not detected	0.3 95% UI:(0.08,0.67)	0.26 95% UI:(0.12,0.48)
eGFR Stage 1, microalbuminuria, detected	0.01 95% UI:(0.0,0.03)	0.03 95% UI:(0.01,0.08)
eGFR Stage 1, macroalbuminuria, detected	0.0 95% UI:(0.0,0.0)	0.0 95% UI:(0.0,0.0)
eGFR Stage 2, microalbuminuria, detected	0.06 95% UI:(0.02,0.15)	0.13 95% UI:(0.05,0.26)
eGFR Stage 2, macroalbuminuria, detected	0.0 95% UI:(0.0,0.02)	0.01 95% UI:(0.0,0.03)
eGFR Stage 3a, no albuminuria, detected	0.13 95% UI:(0.03,0.34)	0.17 95% UI:(0.06,0.4)
eGFR Stage 3a, microalbuminuria, detected	0.07 95% UI:(0.02,0.19)	0.12 95% UI:(0.05,0.26)
eGFR Stage 3a, macroalbuminuria, detected	0.01 95% UI:(0.0,0.04)	0.03 95% UI:(0.01,0.06)
eGFR Stage 3b, no albuminuria, detected	0.2 95% UI:(0.05,0.49)	0.13 95% UI:(0.06,0.3)
eGFR Stage 3b, microalbuminuria, detected	0.07 95% UI:(0.01,0.2)	0.1 95% UI:(0.04,0.2)
eGFR Stage 3b, macroalbuminuria, detected	0.06 95% UI:(0.01,0.18)	0.04 95% UI:(0.01,0.09)
eGFR Stage 4, no albuminuria, detected	0.07 95% UI:(0.01,0.23)	0.11 95% UI:(0.05,0.23)
eGFR Stage 4, microalbuminuria, detected	0.06 95% UI:(0.01,0.17)	0.1 95% UI:(0.05,0.2)
eGFR Stage 4, macroalbuminuria, detected	0.06 95% UI:(0.01,0.2)	0.06 95% UI:(0.02,0.14)
eGFR Stage 1, microalbuminuria, detected and treated	0.01 95% UI:(0.0,0.04)	0.08 95% UI:(0.02,0.18)
eGFR Stage 1, macroalbuminuria, detected and treated	0.0 95% UI:(0.0,0.01)	0.0 95% UI:(0.0,0.01)
eGFR Stage 2, microalbuminuria, detected and treated	0.2 95% UI:(0.08,0.42)	0.31 95% UI:(0.11,0.59)
eGFR Stage 2, macroalbuminuria, detected and treated	0.03 95% UI:(0.01,0.07)	0.03 95% UI:(0.01,0.07)
eGFR Stage 3a, no albuminuria, detected and treated	0.89 95% UI:(0.46,1.53)	0.83 95% UI:(0.4,1.5)
eGFR Stage 3a, microalbuminuria, detected and treated	0.45 95% UI:(0.21,0.81)	0.42 95% UI:(0.22,0.73)
eGFR Stage 3a, macroalbuminuria, detected and treated	0.07 95% UI:(0.02,0.18)	0.09 95% UI:(0.04,0.19)
eGFR Stage 3b, no albuminuria, detected and treated	0.65 95% UI:(0.28,1.21)	0.45 95% UI:(0.22,0.82)
eGFR Stage 3b, microalbuminuria, detected and treated	0.61 95% UI:(0.31,1.04)	0.3 95% UI:(0.14,0.53)
eGFR Stage 3b, macroalbuminuria, detected and treated	0.3 95% UI:(0.11,0.63)	0.11 95% UI:(0.05,0.23)
eGFR Stage 4, no albuminuria, detected and treated	0.19 95% UI:(0.04,0.48)	0.42 95% UI:(0.18,0.81)
eGFR Stage 4, microalbuminuria, detected and treated	0.29 95% UI:(0.1,0.59)	0.35 95% UI:(0.16,0.66)
eGFR Stage 4, macroalbuminuria, detected and treated	0.38 95% UI:(0.12,0.81)	0.25 95% UI:(0.1,0.5)
Kidney failure pre-kidney replacement therapy, no albuminuria	0.0 95% UI:(0.0,0.0)	0.09 95% UI:(0.02,0.22)
Kidney failure pre-kidney replacement therapy, microalbuminuria	0.08 95% UI:(0.0,0.31)	0.11 95% UI:(0.02,0.25)
Kidney failure pre-kidney replacement therapy, macroalbuminuria	0.18 95% UI:(0.0,0.48)	0.14 95% UI:(0.05,0.29)
Kidney failure on kidney replacement therapy, no albuminuria	0.0 95% UI:(0.0,0.0)	0.07 95% UI:(0.01,0.2)
Kidney failure on kidney replacement therapy, microalbuminuria	0.29 95% UI:(0.0,0.9)	0.14 95% UI:(0.02,0.4)
Kidney failure on kidney replacement therapy, macroalbuminuria	0.41 95% UI:(0.01,0.92)	0.9 95% UI:(0.28,1.85)

© 2025 Cusick MM et al. JAMA Network Open.

eTable 13. CKD Distribution Estimates and Calibration Targets (NHANES Estimates) and Calibrated Model Projections for Non-Hispanic Black Adults Aged 70 to 79 Years

	Target	Model
eGFR Stage 1, no albuminuria, not detected	3.83 95% UI:(3.1,4.68)	4.43 95% UI:(1.99,8.07)
eGFR Stage 1, microalbuminuria, not detected	0.81 95% UI:(0.55,1.15)	0.51 95% UI:(0.13,1.15)
eGFR Stage 1, macroalbuminuria, not detected	0.05 95% UI:(0.01,0.15)	0.01 95% UI:(0.0,0.02)
eGFR Stage 2, no albuminuria, not detected	32.52 95% UI:(29.04,35.96)	33.91 95% UI:(27.04,41.02)
eGFR Stage 2, microalbuminuria, not detected	6.32 95% UI:(4.26,8.86)	5.73 95% UI:(3.49,8.2)
eGFR Stage 2, macroalbuminuria, not detected	0.58 95% UI:(0.19,1.38)	0.18 95% UI:(0.05,0.42)
eGFR Stage 3a, no albuminuria, not detected	21.26 95% UI:(18.26,24.26)	19.51 95% UI:(15.07,24.46)
eGFR Stage 3a, microalbuminuria, not detected	6.25 95% UI:(4.54,8.25)	6.52 95% UI:(4.7,8.45)
eGFR Stage 3a, macroalbuminuria, not detected	0.94 95% UI:(0.34,2.07)	0.76 95% UI:(0.35,1.41)
eGFR Stage 3b, no albuminuria, not detected	7.67 95% UI:(5.56,10.0)	7.35 95% UI:(5.27,9.58)
eGFR Stage 3b, microalbuminuria, not detected	2.46 95% UI:(1.37,3.9)	3.73 95% UI:(2.75,4.73)
eGFR Stage 3b, macroalbuminuria, not detected	1.4 95% UI:(0.59,2.71)	0.89 95% UI:(0.44,1.6)
eGFR Stage 4, no albuminuria, not detected	1.36 95% UI:(0.46,2.8)	1.45 95% UI:(0.57,2.62)
eGFR Stage 4, microalbuminuria, not detected	0.84 95% UI:(0.24,1.91)	1.08 95% UI:(0.44,1.97)
eGFR Stage 4, macroalbuminuria, not detected	0.8 95% UI:(0.25,1.76)	0.64 95% UI:(0.27,1.27)
eGFR Stage 1, microalbuminuria, detected	0.0 95% UI:(0.0,0.01)	0.01 95% UI:(0.0,0.03)
eGFR Stage 1, macroalbuminuria, detected	0.0 95% UI:(0.0,0.0)	0.0 95% UI:(0.0,0.0)
eGFR Stage 2, microalbuminuria, detected	0.01 95% UI:(0.0,0.04)	0.1 95% UI:(0.03,0.23)
eGFR Stage 2, macroalbuminuria, detected	0.0 95% UI:(0.0,0.0)	0.0 95% UI:(0.0,0.01)
eGFR Stage 3a, no albuminuria, detected	0.07 95% UI:(0.02,0.18)	0.36 95% UI:(0.16,0.7)
eGFR Stage 3a, microalbuminuria, detected	0.02 95% UI:(0.0,0.09)	0.25 95% UI:(0.12,0.48)
eGFR Stage 3a, macroalbuminuria, detected	0.01 95% UI:(0.0,0.04)	0.04 95% UI:(0.01,0.09)
eGFR Stage 3b, no albuminuria, detected	0.31 95% UI:(0.09,0.76)	0.29 95% UI:(0.14,0.56)
eGFR Stage 3b, microalbuminuria, detected	0.11 95% UI:(0.03,0.29)	0.23 95% UI:(0.11,0.42)
eGFR Stage 3b, macroalbuminuria, detected	0.01 95% UI:(0.0,0.06)	0.06 95% UI:(0.02,0.14)
eGFR Stage 4, no albuminuria, detected	0.06 95% UI:(0.01,0.23)	0.25 95% UI:(0.11,0.5)
eGFR Stage 4, microalbuminuria, detected	0.21 95% UI:(0.03,0.67)	0.24 95% UI:(0.11,0.49)
eGFR Stage 4, macroalbuminuria, detected	0.02 95% UI:(0.0,0.08)	0.14 95% UI:(0.05,0.34)
eGFR Stage 1, microalbuminuria, detected and treated	0.01 95% UI:(0.0,0.01)	0.02 95% UI:(0.0,0.07)
eGFR Stage 1, macroalbuminuria, detected and treated	0.0 95% UI:(0.0,0.0)	0.0 95% UI:(0.0,0.0)
eGFR Stage 2, microalbuminuria, detected and treated	0.1 95% UI:(0.03,0.25)	0.26 95% UI:(0.09,0.53)
eGFR Stage 2, macroalbuminuria, detected and treated	0.01 95% UI:(0.0,0.02)	0.01 95% UI:(0.0,0.04)
eGFR Stage 3a, no albuminuria, detected and treated	0.87 95% UI:(0.43,1.54)	1.95 95% UI:(1.1,3.23)
eGFR Stage 3a, microalbuminuria, detected and treated	0.72 95% UI:(0.27,1.52)	0.97 95% UI:(0.56,1.6)
eGFR Stage 3a, macroalbuminuria, detected and treated	0.06 95% UI:(0.01,0.16)	0.15 95% UI:(0.05,0.3)
eGFR Stage 3b, no albuminuria, detected and treated	1.76 95% UI:(0.87,3.03)	1.18 95% UI:(0.68,2.01)
eGFR Stage 3b, microalbuminuria, detected and treated	1.52 95% UI:(0.77,2.59)	0.76 95% UI:(0.48,1.18)
eGFR Stage 3b, macroalbuminuria, detected and treated	0.6 95% UI:(0.22,1.28)	0.22 95% UI:(0.09,0.4)
eGFR Stage 4, no albuminuria, detected and treated	0.92 95% UI:(0.28,1.99)	1.22 95% UI:(0.55,2.21)
eGFR Stage 4, microalbuminuria, detected and treated	1.15 95% UI:(0.38,2.37)	1.04 95% UI:(0.49,1.93)
eGFR Stage 4, macroalbuminuria, detected and treated	0.88 95% UI:(0.29,1.89)	0.63 95% UI:(0.24,1.32)
Kidney failure pre-kidney replacement therapy, no albuminuria	0.0 95% UI:(0.0,0.0)	0.24 95% UI:(0.07,0.53)
Kidney failure pre-kidney replacement therapy, microalbuminuria	0.54 95% UI:(0.0,1.78)	0.3 95% UI:(0.09,0.65)
Kidney failure pre-kidney replacement therapy, macroalbuminuria	0.79 95% UI:(0.01,2.2)	0.34 95% UI:(0.13,0.7)
Kidney failure on kidney replacement therapy, no albuminuria	0.0 95% UI:(0.0,0.0)	0.14 95% UI:(0.03,0.39)
Kidney failure on kidney replacement therapy, microalbuminuria	1.07 95% UI:(0.01,2.97)	0.29 95% UI:(0.07,0.72)
Kidney failure on kidney replacement therapy, macroalbuminuria	1.07 95% UI:(0.01,2.75)	1.59 95% UI:(0.65,2.91)

© 2025 Cusick MM et al. JAMA Network Open.

eTable 14. CKD Distribution Estimates and Calibration Targets (NHANES Estimates) and Calibrated Model Projections for Non-Hispanic White Adults Aged 40 to 49 Years

	Target	Model
eGFR Stage 1, no albuminuria, not detected	60.25 95% UI:(53.07,64.96)	58.95 95% UI:(51.11,67.37)
eGFR Stage 1, microalbuminuria, not detected	3.52 95% UI:(2.48,4.79)	4.4 95% UI:(3.12,5.97)
eGFR Stage 1, macroalbuminuria, not detected	1.05 95% UI:(0.01,7.56)	0.35 95% UI:(0.13,0.78)
eGFR Stage 2, no albuminuria, not detected	31.47 95% UI:(27.15,35.54)	31.42 95% UI:(23.26,39.2)
eGFR Stage 2, microalbuminuria, not detected	1.23 95% UI:(0.76,1.87)	2.42 95% UI:(1.48,3.59)
eGFR Stage 2, macroalbuminuria, not detected	0.41 95% UI:(0.0,3.01)	0.47 95% UI:(0.18,1.08)
eGFR Stage 3a, no albuminuria, not detected	1.18 95% UI:(0.8,1.56)	0.83 95% UI:(0.33,2.04)
eGFR Stage 3a, microalbuminuria, not detected	0.07 95% UI:(0.03,0.13)	0.14 95% UI:(0.05,0.34)
eGFR Stage 3a, macroalbuminuria, not detected	0.05 95% UI:(0.0,0.34)	0.04 95% UI:(0.01,0.11)
eGFR Stage 3b, no albuminuria, not detected	0.24 95% UI:(0.11,0.34)	0.24 95% UI:(0.11,0.47)
eGFR Stage 3b, microalbuminuria, not detected	0.02 95% UI:(0.01,0.04)	0.06 95% UI:(0.02,0.11)
eGFR Stage 3b, macroalbuminuria, not detected	0.02 95% UI:(0.0,0.13)	0.02 95% UI:(0.0,0.06)
eGFR Stage 4, no albuminuria, not detected	0.02 95% UI:(0.0,0.04)	0.04 95% UI:(0.0,0.09)
eGFR Stage 4, microalbuminuria, not detected	0.0 95% UI:(0.0,0.01)	0.01 95% UI:(0.0,0.03)
eGFR Stage 4, macroalbuminuria, not detected	0.01 95% UI:(0.0,0.03)	0.01 95% UI:(0.0,0.03)
eGFR Stage 1, microalbuminuria, detected	0.07 95% UI:(0.03,0.17)	0.05 95% UI:(0.02,0.1)
eGFR Stage 1, macroalbuminuria, detected	0.02 95% UI:(0.0,0.16)	0.01 95% UI:(0.0,0.02)
eGFR Stage 2, microalbuminuria, detected	0.04 95% UI:(0.02,0.08)	0.03 95% UI:(0.01,0.07)
eGFR Stage 2, macroalbuminuria, detected	0.01 95% UI:(0.0,0.11)	0.01 95% UI:(0.0,0.03)
eGFR Stage 3a, no albuminuria, detected	0.07 95% UI:(0.02,0.13)	0.04 95% UI:(0.02,0.09)
eGFR Stage 3a, microalbuminuria, detected	0.01 95% UI:(0.0,0.02)	0.01 95% UI:(0.0,0.02)
eGFR Stage 3a, macroalbuminuria, detected	0.01 95% UI:(0.0,0.04)	0.0 95% UI:(0.0,0.01)
eGFR Stage 3b, no albuminuria, detected	0.03 95% UI:(0.01,0.06)	0.02 95% UI:(0.01,0.04)
eGFR Stage 3b, microalbuminuria, detected	0.01 95% UI:(0.0,0.01)	0.01 95% UI:(0.0,0.02)
eGFR Stage 3b, macroalbuminuria, detected	0.0 95% UI:(0.0,0.03)	0.0 95% UI:(0.0,0.01)
eGFR Stage 4, no albuminuria, detected	0.01 95% UI:(0.0,0.02)	0.01 95% UI:(0.0,0.02)
eGFR Stage 4, microalbuminuria, detected	0.0 95% UI:(0.0,0.01)	0.0 95% UI:(0.0,0.01)
eGFR Stage 4, macroalbuminuria, detected	0.01 95% UI:(0.0,0.02)	0.0 95% UI:(0.0,0.01)
eGFR Stage 1, microalbuminuria, detected and treated	0.03 95% UI:(0.01,0.08)	0.05 95% UI:(0.02,0.1)
eGFR Stage 1, macroalbuminuria, detected and treated	0.01 95% UI:(0.0,0.1)	0.01 95% UI:(0.0,0.02)
eGFR Stage 2, microalbuminuria, detected and treated	0.01 95% UI:(0.0,0.03)	0.03 95% UI:(0.01,0.07)
eGFR Stage 2, macroalbuminuria, detected and treated	0.01 95% UI:(0.0,0.06)	0.01 95% UI:(0.0,0.04)
eGFR Stage 3a, no albuminuria, detected and treated	0.04 95% UI:(0.01,0.09)	0.09 95% UI:(0.04,0.18)
eGFR Stage 3a, microalbuminuria, detected and treated	0.01 95% UI:(0.0,0.02)	0.02 95% UI:(0.01,0.05)
eGFR Stage 3a, macroalbuminuria, detected and treated	0.01 95% UI:(0.0,0.05)	0.0 95% UI:(0.0,0.01)
eGFR Stage 3b, no albuminuria, detected and treated	0.02 95% UI:(0.0,0.05)	0.04 95% UI:(0.02,0.08)
eGFR Stage 3b, microalbuminuria, detected and treated	0.0 95% UI:(0.0,0.01)	0.01 95% UI:(0.0,0.02)
eGFR Stage 3b, macroalbuminuria, detected and treated	0.01 95% UI:(0.0,0.04)	0.0 95% UI:(0.0,0.01)
eGFR Stage 4, no albuminuria, detected and treated	0.0 95% UI:(0.0,0.01)	0.03 95% UI:(0.01,0.06)
eGFR Stage 4, microalbuminuria, detected and treated	0.0 95% UI:(0.0,0.01)	0.01 95% UI:(0.0,0.02)
eGFR Stage 4, macroalbuminuria, detected and treated	0.01 95% UI:(0.0,0.02)	0.01 95% UI:(0.0,0.02)
Kidney failure pre-kidney replacement therapy, no albuminuria	0.0 95% UI:(0.0,0.0)	0.01 95% UI:(0.0,0.02)
Kidney failure pre-kidney replacement therapy, microalbuminuria	0.0 95% UI:(0.0,0.0)	0.0 95% UI:(0.0,0.01)
Kidney failure pre-kidney replacement therapy, macroalbuminuria	0.0 95% UI:(0.0,0.0)	0.0 95% UI:(0.0,0.02)
Kidney failure on kidney replacement therapy, no albuminuria	0.01 95% UI:(0.0,0.03)	0.0 95% UI:(0.0,0.02)
Kidney failure on kidney replacement therapy, microalbuminuria	0.01 95% UI:(0.0,0.03)	0.01 95% UI:(0.0,0.02)
Kidney failure on kidney replacement therapy, macroalbuminuria	0.01 95% UI:(0.0,0.03)	0.03 95% UI:(0.01,0.08)

© 2025 Cusick MM et al. JAMA Network Open.

eTable 15. CKD Distribution Estimates and Calibration Targets (NHANES Estimates) and Calibrated Model Projections for Non-Hispanic White Adults Aged 50 to 59 Years

	Target	Model
eGFR Stage 1, no albuminuria, not detected	46.5 95% UI:(42.93,50.05)	45.58 95% UI:(34.55,57.12)
eGFR Stage 1, microalbuminuria, not detected	4.09 95% UI:(2.66,5.98)	4.58 95% UI:(2.83,6.67)
eGFR Stage 1, macroalbuminuria, not detected	1.01 95% UI:(0.44,1.96)	0.25 95% UI:(0.08,0.57)
eGFR Stage 2, no albuminuria, not detected	41.94 95% UI:(38.89,44.97)	40.68 95% UI:(29.19,51.44)
eGFR Stage 2, microalbuminuria, not detected	2.44 95% UI:(1.65,3.45)	4.62 95% UI:(2.77,6.74)
eGFR Stage 2, macroalbuminuria, not detected	0.59 95% UI:(0.24,1.22)	0.75 95% UI:(0.34,1.45)
eGFR Stage 3a, no albuminuria, not detected	1.93 95% UI:(1.51,2.43)	1.37 95% UI:(0.4,3.56)
eGFR Stage 3a, microalbuminuria, not detected	0.17 95% UI:(0.09,0.28)	0.32 95% UI:(0.09,0.84)
eGFR Stage 3a, macroalbuminuria, not detected	0.11 95% UI:(0.04,0.24)	0.09 95% UI:(0.02,0.25)
eGFR Stage 3b, no albuminuria, not detected	0.39 95% UI:(0.28,0.51)	0.36 95% UI:(0.13,0.96)
eGFR Stage 3b, microalbuminuria, not detected	0.05 95% UI:(0.03,0.08)	0.13 95% UI:(0.04,0.31)
eGFR Stage 3b, macroalbuminuria, not detected	0.05 95% UI:(0.02,0.1)	0.05 95% UI:(0.01,0.14)
eGFR Stage 4, no albuminuria, not detected	0.02 95% UI:(0.01,0.05)	0.06 95% UI:(0.01,0.19)
eGFR Stage 4, microalbuminuria, not detected	0.01 95% UI:(0.0,0.01)	0.03 95% UI:(0.0,0.09)
eGFR Stage 4, macroalbuminuria, not detected	0.02 95% UI:(0.01,0.04)	0.02 95% UI:(0.0,0.07)
eGFR Stage 1, microalbuminuria, detected	0.06 95% UI:(0.02,0.13)	0.04 95% UI:(0.02,0.1)
eGFR Stage 1, macroalbuminuria, detected	0.01 95% UI:(0.0,0.04)	0.0 95% UI:(0.0,0.01)
eGFR Stage 2, microalbuminuria, detected	0.06 95% UI:(0.02,0.14)	0.05 95% UI:(0.02,0.1)
eGFR Stage 2, macroalbuminuria, detected	0.01 95% UI:(0.0,0.05)	0.02 95% UI:(0.0,0.04)
eGFR Stage 3a, no albuminuria, detected	0.07 95% UI:(0.02,0.15)	0.07 95% UI:(0.02,0.17)
eGFR Stage 3a, microalbuminuria, detected	0.01 95% UI:(0.0,0.03)	0.02 95% UI:(0.01,0.06)
eGFR Stage 3a, macroalbuminuria, detected	0.01 95% UI:(0.0,0.03)	0.01 95% UI:(0.0,0.02)
eGFR Stage 3b, no albuminuria, detected	0.04 95% UI:(0.01,0.08)	0.04 95% UI:(0.01,0.09)
eGFR Stage 3b, microalbuminuria, detected	0.01 95% UI:(0.0,0.02)	0.02 95% UI:(0.01,0.04)
eGFR Stage 3b, macroalbuminuria, detected	0.01 95% UI:(0.0,0.02)	0.01 95% UI:(0.0,0.02)
eGFR Stage 4, no albuminuria, detected	0.01 95% UI:(0.0,0.02)	0.02 95% UI:(0.0,0.06)
eGFR Stage 4, microalbuminuria, detected	0.0 95% UI:(0.0,0.01)	0.01 95% UI:(0.0,0.03)
eGFR Stage 4, macroalbuminuria, detected	0.01 95% UI:(0.0,0.02)	0.01 95% UI:(0.0,0.02)
eGFR Stage 1, microalbuminuria, detected and treated	0.05 95% UI:(0.01,0.11)	0.06 95% UI:(0.02,0.12)
eGFR Stage 1, macroalbuminuria, detected and treated	0.01 95% UI:(0.0,0.04)	0.01 95% UI:(0.0,0.02)
eGFR Stage 2, microalbuminuria, detected and treated	0.04 95% UI:(0.01,0.09)	0.07 95% UI:(0.03,0.12)
eGFR Stage 2, macroalbuminuria, detected and treated	0.02 95% UI:(0.0,0.05)	0.02 95% UI:(0.01,0.06)
eGFR Stage 3a, no albuminuria, detected and treated	0.09 95% UI:(0.03,0.19)	0.16 95% UI:(0.05,0.42)
eGFR Stage 3a, microalbuminuria, detected and treated	0.02 95% UI:(0.01,0.05)	0.05 95% UI:(0.01,0.13)
eGFR Stage 3a, macroalbuminuria, detected and treated	0.02 95% UI:(0.0,0.05)	0.01 95% UI:(0.0,0.04)
eGFR Stage 3b, no albuminuria, detected and treated	0.03 95% UI:(0.01,0.06)	0.08 95% UI:(0.03,0.19)
eGFR Stage 3b, microalbuminuria, detected and treated	0.01 95% UI:(0.0,0.03)	0.03 95% UI:(0.01,0.07)
eGFR Stage 3b, macroalbuminuria, detected and treated	0.02 95% UI:(0.01,0.05)	0.01 95% UI:(0.0,0.03)
eGFR Stage 4, no albuminuria, detected and treated	0.01 95% UI:(0.0,0.01)	0.06 95% UI:(0.01,0.15)
eGFR Stage 4, microalbuminuria, detected and treated	0.0 95% UI:(0.0,0.01)	0.03 95% UI:(0.01,0.07)
eGFR Stage 4, macroalbuminuria, detected and treated	0.02 95% UI:(0.01,0.04)	0.02 95% UI:(0.01,0.05)
Kidney failure pre-kidney replacement therapy, no albuminuria	0.0 95% UI:(0.0,0.0)	0.01 95% UI:(0.0,0.05)
Kidney failure pre-kidney replacement therapy, microalbuminuria	0.0 95% UI:(0.0,0.0)	0.01 95% UI:(0.0,0.04)
Kidney failure pre-kidney replacement therapy, macroalbuminuria	0.0 95% UI:(0.0,0.0)	0.01 95% UI:(0.0,0.04)
Kidney failure on kidney replacement therapy, no albuminuria	0.01 95% UI:(0.0,0.04)	0.01 95% UI:(0.0,0.06)
Kidney failure on kidney replacement therapy, microalbuminuria	0.01 95% UI:(0.0,0.05)	0.02 95% UI:(0.0,0.07)
Kidney failure on kidney replacement therapy, macroalbuminuria	0.02 95% UI:(0.0,0.05)	0.1 95% UI:(0.01,0.29)

© 2025 Cusick MM et al. JAMA Network Open.

eTable 16. CKD Distribution Estimates and Calibration Targets (NHANES Estimates) and Calibrated Model Projections for Non-Hispanic White Adults Aged 60 to 69 Years

	Target	Model
eGFR Stage 1, no albuminuria, not detected	20.95 95% UI:(18.09,24.0)	23.43 95% UI:(16.63,30.68)
eGFR Stage 1, microalbuminuria, not detected	3.19 95% UI:(2.15,4.5)	2.62 95% UI:(1.39,4.22)
eGFR Stage 1, macroalbuminuria, not detected	0.3 95% UI:(0.09,0.76)	0.07 95% UI:(0.02,0.15)
eGFR Stage 2, no albuminuria, not detected	58.9 95% UI:(55.48,62.09)	52.66 95% UI:(45.5,59.15)
eGFR Stage 2, microalbuminuria, not detected	5.97 95% UI:(4.07,8.39)	7.47 95% UI:(4.89,10.07)
eGFR Stage 2, macroalbuminuria, not detected	0.52 95% UI:(0.2,1.09)	0.75 95% UI:(0.33,1.3)
eGFR Stage 3a, no albuminuria, not detected	5.47 95% UI:(4.44,6.62)	6.54 95% UI:(4.26,9.27)
eGFR Stage 3a, microalbuminuria, not detected	0.78 95% UI:(0.48,1.17)	1.59 95% UI:(0.92,2.49)
eGFR Stage 3a, macroalbuminuria, not detected	0.2 95% UI:(0.05,0.55)	0.37 95% UI:(0.18,0.66)
eGFR Stage 3b, no albuminuria, not detected	1.09 95% UI:(0.81,1.4)	1.19 95% UI:(0.64,2.07)
eGFR Stage 3b, microalbuminuria, not detected	0.22 95% UI:(0.12,0.34)	0.44 95% UI:(0.2,0.81)
eGFR Stage 3b, macroalbuminuria, not detected	0.09 95% UI:(0.03,0.2)	0.21 95% UI:(0.1,0.4)
eGFR Stage 4, no albuminuria, not detected	0.07 95% UI:(0.02,0.14)	0.16 95% UI:(0.05,0.36)
eGFR Stage 4, microalbuminuria, not detected	0.03 95% UI:(0.01,0.07)	0.09 95% UI:(0.02,0.21)
eGFR Stage 4, macroalbuminuria, not detected	0.05 95% UI:(0.01,0.1)	0.1 95% UI:(0.04,0.22)
eGFR Stage 1, microalbuminuria, detected	0.04 95% UI:(0.01,0.1)	0.03 95% UI:(0.01,0.06)
eGFR Stage 1, macroalbuminuria, detected	0.0 95% UI:(0.0,0.01)	0.0 95% UI:(0.0,0.0)
eGFR Stage 2, microalbuminuria, detected	0.15 95% UI:(0.06,0.3)	0.07 95% UI:(0.03,0.14)
eGFR Stage 2, macroalbuminuria, detected	0.01 95% UI:(0.0,0.04)	0.01 95% UI:(0.0,0.04)
eGFR Stage 3a, no albuminuria, detected	0.16 95% UI:(0.07,0.31)	0.13 95% UI:(0.04,0.32)
eGFR Stage 3a, microalbuminuria, detected	0.04 95% UI:(0.01,0.09)	0.06 95% UI:(0.02,0.14)
eGFR Stage 3a, macroalbuminuria, detected	0.01 95% UI:(0.0,0.04)	0.02 95% UI:(0.0,0.04)
eGFR Stage 3b, no albuminuria, detected	0.05 95% UI:(0.02,0.11)	0.07 95% UI:(0.03,0.17)
eGFR Stage 3b, microalbuminuria, detected	0.02 95% UI:(0.01,0.05)	0.04 95% UI:(0.01,0.08)
eGFR Stage 3b, macroalbuminuria, detected	0.01 95% UI:(0.0,0.03)	0.02 95% UI:(0.0,0.04)
eGFR Stage 4, no albuminuria, detected	0.01 95% UI:(0.0,0.04)	0.04 95% UI:(0.01,0.1)
eGFR Stage 4, microalbuminuria, detected	0.01 95% UI:(0.0,0.04)	0.03 95% UI:(0.01,0.06)
eGFR Stage 4, macroalbuminuria, detected	0.02 95% UI:(0.0,0.05)	0.02 95% UI:(0.01,0.05)
eGFR Stage 1, microalbuminuria, detected and treated	0.06 95% UI:(0.02,0.13)	0.04 95% UI:(0.02,0.09)
eGFR Stage 1, macroalbuminuria, detected and treated	0.01 95% UI:(0.0,0.03)	0.0 95% UI:(0.0,0.01)
eGFR Stage 2, microalbuminuria, detected and treated	0.16 95% UI:(0.07,0.32)	0.12 95% UI:(0.05,0.2)
eGFR Stage 2, macroalbuminuria, detected and treated	0.02 95% UI:(0.0,0.06)	0.02 95% UI:(0.01,0.05)
eGFR Stage 3a, no albuminuria, detected and treated	0.43 95% UI:(0.24,0.71)	0.48 95% UI:(0.21,0.97)
eGFR Stage 3a, microalbuminuria, detected and treated	0.22 95% UI:(0.1,0.38)	0.17 95% UI:(0.07,0.35)
eGFR Stage 3a, macroalbuminuria, detected and treated	0.05 95% UI:(0.01,0.15)	0.04 95% UI:(0.01,0.1)
eGFR Stage 3b, no albuminuria, detected and treated	0.27 95% UI:(0.15,0.42)	0.19 95% UI:(0.08,0.41)
eGFR Stage 3b, microalbuminuria, detected and treated	0.13 95% UI:(0.07,0.22)	0.09 95% UI:(0.04,0.18)
eGFR Stage 3b, macroalbuminuria, detected and treated	0.05 95% UI:(0.01,0.12)	0.04 95% UI:(0.01,0.09)
eGFR Stage 4, no albuminuria, detected and treated	0.04 95% UI:(0.01,0.09)	0.11 95% UI:(0.02,0.28)
eGFR Stage 4, microalbuminuria, detected and treated	0.03 95% UI:(0.01,0.08)	0.07 95% UI:(0.02,0.17)
eGFR Stage 4, macroalbuminuria, detected and treated	0.06 95% UI:(0.02,0.13)	0.06 95% UI:(0.02,0.13)
Kidney failure pre-kidney replacement therapy, no albuminuria	0.0 95% UI:(0.0,0.0)	0.02 95% UI:(0.0,0.09)
Kidney failure pre-kidney replacement therapy, microalbuminuria	0.0 95% UI:(0.0,0.0)	0.02 95% UI:(0.0,0.08)
Kidney failure pre-kidney replacement therapy, macroalbuminuria	0.0 95% UI:(0.0,0.0)	0.03 95% UI:(0.0,0.09)
Kidney failure on kidney replacement therapy, no albuminuria	0.02 95% UI:(0.0,0.14)	0.02 95% UI:(0.0,0.1)
Kidney failure on kidney replacement therapy, microalbuminuria	0.05 95% UI:(0.0,0.17)	0.04 95% UI:(0.0,0.14)
Kidney failure on kidney replacement therapy, macroalbuminuria	0.04 95% UI:(0.0,0.16)	0.23 95% UI:(0.02,0.68)

© 2025 Cusick MM et al. JAMA Network Open.

eTable 17. CKD Distribution Estimates and Calibration Targets (NHANES Estimates) and Calibrated Model Projections for Non-Hispanic White Adults Aged 70 to 79 Years

	Target	Model
eGFR Stage 1, no albuminuria, not detected	4.44 95% UI:(3.69,5.26)	5.42 95% UI:(2.83,9.02)
eGFR Stage 1, microalbuminuria, not detected	1.04 95% UI:(0.68,1.5)	0.56 95% UI:(0.17,1.15)
eGFR Stage 1, macroalbuminuria, not detected	0.08 95% UI:(0.03,0.18)	0.0 95% UI:(0.0,0.02)
eGFR Stage 2, no albuminuria, not detected	48.85 95% UI:(46.14,51.52)	49.49 95% UI:(42.14,57.09)
eGFR Stage 2, microalbuminuria, not detected	7.42 95% UI:(5.97,9.07)	7.51 95% UI:(4.72,10.52)
eGFR Stage 2, macroalbuminuria, not detected	0.6 95% UI:(0.18,1.48)	0.35 95% UI:(0.1,0.7)
eGFR Stage 3a, no albuminuria, not detected	17.34 95% UI:(15.28,19.44)	16.87 95% UI:(12.28,21.86)
eGFR Stage 3a, microalbuminuria, not detected	3.84 95% UI:(2.92,4.86)	4.63 95% UI:(3.05,6.54)
eGFR Stage 3a, macroalbuminuria, not detected	0.73 95% UI:(0.38,1.24)	0.65 95% UI:(0.27,1.22)
eGFR Stage 3b, no albuminuria, not detected	5.24 95% UI:(4.15,6.41)	4.36 95% UI:(2.69,6.14)
eGFR Stage 3b, microalbuminuria, not detected	1.66 95% UI:(1.09,2.38)	1.73 95% UI:(1.0,2.58)
eGFR Stage 3b, macroalbuminuria, not detected	0.56 95% UI:(0.34,0.85)	0.55 95% UI:(0.27,0.92)
eGFR Stage 4, no albuminuria, not detected	0.29 95% UI:(0.09,0.62)	0.69 95% UI:(0.21,1.41)
eGFR Stage 4, microalbuminuria, not detected	0.24 95% UI:(0.09,0.5)	0.39 95% UI:(0.13,0.91)
eGFR Stage 4, macroalbuminuria, not detected	0.3 95% UI:(0.12,0.58)	0.33 95% UI:(0.1,0.73)
eGFR Stage 1, microalbuminuria, detected	0.01 95% UI:(0.0,0.02)	0.01 95% UI:(0.0,0.02)
eGFR Stage 1, macroalbuminuria, detected	0.0 95% UI:(0.0,0.0)	0.0 95% UI:(0.0,0.0)
eGFR Stage 2, microalbuminuria, detected	0.1 95% UI:(0.04,0.2)	0.08 95% UI:(0.03,0.15)
eGFR Stage 2, macroalbuminuria, detected	0.01 95% UI:(0.0,0.02)	0.01 95% UI:(0.0,0.02)
eGFR Stage 3a, no albuminuria, detected	0.33 95% UI:(0.12,0.68)	0.38 95% UI:(0.16,0.8)
eGFR Stage 3a, microalbuminuria, detected	0.15 95% UI:(0.05,0.33)	0.18 95% UI:(0.08,0.36)
eGFR Stage 3a, macroalbuminuria, detected	0.02 95% UI:(0.0,0.06)	0.03 95% UI:(0.01,0.08)
eGFR Stage 3b, no albuminuria, detected	0.15 95% UI:(0.05,0.33)	0.21 95% UI:(0.09,0.42)
eGFR Stage 3b, microalbuminuria, detected	0.11 95% UI:(0.04,0.24)	0.11 95% UI:(0.05,0.22)
eGFR Stage 3b, macroalbuminuria, detected	0.03 95% UI:(0.0,0.09)	0.04 95% UI:(0.01,0.09)
eGFR Stage 4, no albuminuria, detected	0.04 95% UI:(0.01,0.12)	0.11 95% UI:(0.04,0.24)
eGFR Stage 4, microalbuminuria, detected	0.05 95% UI:(0.01,0.14)	0.08 95% UI:(0.03,0.17)
eGFR Stage 4, macroalbuminuria, detected	0.05 95% UI:(0.01,0.14)	0.06 95% UI:(0.01,0.17)
eGFR Stage 1, microalbuminuria, detected and treated	0.02 95% UI:(0.01,0.04)	0.01 95% UI:(0.0,0.03)
eGFR Stage 1, macroalbuminuria, detected and treated	0.0 95% UI:(0.0,0.01)	0.0 95% UI:(0.0,0.0)
eGFR Stage 2, microalbuminuria, detected and treated	0.28 95% UI:(0.14,0.49)	0.14 95% UI:(0.06,0.23)
eGFR Stage 2, macroalbuminuria, detected and treated	0.02 95% UI:(0.0,0.07)	0.01 95% UI:(0.0,0.03)
eGFR Stage 3a, no albuminuria, detected and treated	1.81 95% UI:(1.04,2.86)	1.58 95% UI:(0.84,2.57)
eGFR Stage 3a, microalbuminuria, detected and treated	0.98 95% UI:(0.54,1.58)	0.58 95% UI:(0.3,1.0)
eGFR Stage 3a, macroalbuminuria, detected and treated	0.21 95% UI:(0.07,0.46)	0.1 95% UI:(0.03,0.23)
eGFR Stage 3b, no albuminuria, detected and treated	0.72 95% UI:(0.44,1.09)	0.65 95% UI:(0.34,1.07)
eGFR Stage 3b, microalbuminuria, detected and treated	0.67 95% UI:(0.39,1.05)	0.31 95% UI:(0.15,0.52)
eGFR Stage 3b, macroalbuminuria, detected and treated	0.21 95% UI:(0.09,0.4)	0.11 95% UI:(0.04,0.22)
eGFR Stage 4, no albuminuria, detected and treated	0.18 95% UI:(0.05,0.41)	0.38 95% UI:(0.14,0.78)
eGFR Stage 4, microalbuminuria, detected and treated	0.23 95% UI:(0.08,0.47)	0.25 95% UI:(0.09,0.54)
eGFR Stage 4, macroalbuminuria, detected and treated	0.34 95% UI:(0.14,0.64)	0.21 95% UI:(0.06,0.5)
Kidney failure pre-kidney replacement therapy, no albuminuria	0.0 95% UI:(0.0,0.0)	0.07 95% UI:(0.01,0.21)
Kidney failure pre-kidney replacement therapy, microalbuminuria	0.0 95% UI:(0.0,0.0)	0.07 95% UI:(0.01,0.2)
Kidney failure pre-kidney replacement therapy, macroalbuminuria	0.0 95% UI:(0.0,0.0)	0.1 95% UI:(0.02,0.25)
Kidney failure on kidney replacement therapy, no albuminuria	0.11 95% UI:(0.0,0.73)	0.04 95% UI:(0.0,0.17)
Kidney failure on kidney replacement therapy, microalbuminuria	0.29 95% UI:(0.0,0.98)	0.07 95% UI:(0.01,0.25)
Kidney failure on kidney replacement therapy, macroalbuminuria	0.24 95% UI:(0.0,0.89)	0.49 95% UI:(0.06,1.24)

© 2025 Cusick MM et al. JAMA Network Open.

eTable 18. CKD Distribution Estimates and Calibration Targets (NHANES Estimates) and Calibrated Model Projections for Adults From Additional Racial and Ethnic Groups Aged 40 to 49 Years

	Target	Model
eGFR Stage 1, no albuminuria, not detected	71.66 95% UI:(65.41,77.22)	69.16 95% UI:(63.17,75.86)
eGFR Stage 1, microalbuminuria, not detected	4.63 95% UI:(3.21,6.43)	5.81 95% UI:(4.15,7.61)
eGFR Stage 1, macroalbuminuria, not detected	0.79 95% UI:(0.19,2.19)	0.52 95% UI:(0.12,1.51)
eGFR Stage 2, no albuminuria, not detected	19.62 95% UI:(14.74,25.24)	20.57 95% UI:(14.31,26.26)
eGFR Stage 2, microalbuminuria, not detected	1.12 95% UI:(0.66,1.76)	2.0 95% UI:(1.17,3.0)
eGFR Stage 2, macroalbuminuria, not detected	0.49 95% UI:(0.09,1.55)	0.54 95% UI:(0.1,1.65)
eGFR Stage 3a, no albuminuria, not detected	0.59 95% UI:(0.23,1.05)	0.42 95% UI:(0.14,1.02)
eGFR Stage 3a, microalbuminuria, not detected	0.06 95% UI:(0.01,0.14)	0.1 95% UI:(0.03,0.22)
eGFR Stage 3a, macroalbuminuria, not detected	0.15 95% UI:(0.01,0.49)	0.04 95% UI:(0.01,0.12)
eGFR Stage 3b, no albuminuria, not detected	0.07 95% UI:(0.0,0.19)	0.13 95% UI:(0.05,0.28)
eGFR Stage 3b, microalbuminuria, not detected	0.02 95% UI:(0.0,0.1)	0.04 95% UI:(0.02,0.09)
eGFR Stage 3b, macroalbuminuria, not detected	0.04 95% UI:(0.0,0.14)	0.03 95% UI:(0.01,0.09)
eGFR Stage 4, no albuminuria, not detected	0.02 95% UI:(0.0,0.09)	0.03 95% UI:(0.0,0.06)
eGFR Stage 4, microalbuminuria, not detected	0.01 95% UI:(0.0,0.03)	0.01 95% UI:(0.0,0.03)
eGFR Stage 4, macroalbuminuria, not detected	0.01 95% UI:(0.0,0.04)	0.02 95% UI:(0.0,0.05)
eGFR Stage 1, microalbuminuria, detected	0.13 95% UI:(0.04,0.31)	0.06 95% UI:(0.02,0.15)
eGFR Stage 1, macroalbuminuria, detected	0.05 95% UI:(0.0,0.19)	0.02 95% UI:(0.0,0.07)
eGFR Stage 2, microalbuminuria, detected	0.07 95% UI:(0.02,0.18)	0.03 95% UI:(0.01,0.08)
eGFR Stage 2, macroalbuminuria, detected	0.07 95% UI:(0.01,0.26)	0.03 95% UI:(0.0,0.1)
eGFR Stage 3a, no albuminuria, detected	0.01 95% UI:(0.0,0.04)	0.01 95% UI:(0.0,0.02)
eGFR Stage 3a, microalbuminuria, detected	0.0 95% UI:(0.0,0.01)	0.0 95% UI:(0.0,0.01)
eGFR Stage 3a, macroalbuminuria, detected	0.02 95% UI:(0.0,0.08)	0.0 95% UI:(0.0,0.01)
eGFR Stage 3b, no albuminuria, detected	0.01 95% UI:(0.0,0.02)	0.01 95% UI:(0.0,0.01)
eGFR Stage 3b, microalbuminuria, detected	0.0 95% UI:(0.0,0.02)	0.0 95% UI:(0.0,0.01)
eGFR Stage 3b, macroalbuminuria, detected	0.01 95% UI:(0.0,0.05)	0.0 95% UI:(0.0,0.01)
eGFR Stage 4, no albuminuria, detected	0.01 95% UI:(0.0,0.06)	0.01 95% UI:(0.0,0.02)
eGFR Stage 4, microalbuminuria, detected	0.01 95% UI:(0.0,0.05)	0.0 95% UI:(0.0,0.01)
eGFR Stage 4, macroalbuminuria, detected	0.02 95% UI:(0.0,0.08)	0.01 95% UI:(0.0,0.02)
eGFR Stage 1, microalbuminuria, detected and treated	0.06 95% UI:(0.01,0.16)	0.08 95% UI:(0.03,0.17)
eGFR Stage 1, macroalbuminuria, detected and treated	0.03 95% UI:(0.0,0.14)	0.02 95% UI:(0.0,0.08)
eGFR Stage 2, microalbuminuria, detected and treated	0.03 95% UI:(0.0,0.09)	0.03 95% UI:(0.01,0.08)
eGFR Stage 2, macroalbuminuria, detected and treated	0.04 95% UI:(0.0,0.17)	0.03 95% UI:(0.0,0.11)
eGFR Stage 3a, no albuminuria, detected and treated	0.01 95% UI:(0.0,0.03)	0.04 95% UI:(0.01,0.08)
eGFR Stage 3a, microalbuminuria, detected and treated	0.0 95% UI:(0.0,0.01)	0.01 95% UI:(0.0,0.03)
eGFR Stage 3a, macroalbuminuria, detected and treated	0.02 95% UI:(0.0,0.09)	0.01 95% UI:(0.0,0.02)
eGFR Stage 3b, no albuminuria, detected and treated	0.0 95% UI:(0.0,0.02)	0.02 95% UI:(0.01,0.03)
eGFR Stage 3b, microalbuminuria, detected and treated	0.0 95% UI:(0.0,0.02)	0.01 95% UI:(0.0,0.01)
eGFR Stage 3b, macroalbuminuria, detected and treated	0.02 95% UI:(0.0,0.06)	0.01 95% UI:(0.0,0.02)
eGFR Stage 4, no albuminuria, detected and treated	0.01 95% UI:(0.0,0.04)	0.02 95% UI:(0.0,0.04)
eGFR Stage 4, microalbuminuria, detected and treated	0.01 95% UI:(0.0,0.04)	0.01 95% UI:(0.0,0.02)
eGFR Stage 4, macroalbuminuria, detected and treated	0.02 95% UI:(0.0,0.09)	0.01 95% UI:(0.0,0.04)
Kidney failure pre-kidney replacement therapy, no albuminuria	0.01 95% UI:(0.0,0.06)	0.01 95% UI:(0.0,0.02)
Kidney failure pre-kidney replacement therapy, microalbuminuria	0.0 95% UI:(0.0,0.0)	0.0 95% UI:(0.0,0.01)
Kidney failure pre-kidney replacement therapy, macroalbuminuria	0.0 95% UI:(0.0,0.03)	0.01 95% UI:(0.0,0.03)
Kidney failure on kidney replacement therapy, no albuminuria	0.02 95% UI:(0.0,0.09)	0.01 95% UI:(0.0,0.03)
Kidney failure on kidney replacement therapy, microalbuminuria	0.0 95% UI:(0.0,0.0)	0.01 95% UI:(0.0,0.03)
Kidney failure on kidney replacement therapy, macroalbuminuria	0.04 95% UI:(0.0,0.14)	0.08 95% UI:(0.02,0.19)

© 2025 Cusick MM et al. JAMA Network Open.

eTable 19. CKD Distribution Estimates and Calibration Targets (NHANES Estimates) and Calibrated Model Projections for Adults From Additional Racial and Ethnic Groups Aged 50 to 59 Years

	Target	Model
eGFR Stage 1, no albuminuria, not detected	50.11 95% UI:(41.27,58.69)	56.76 95% UI:(47.89,65.48)
eGFR Stage 1, microalbuminuria, not detected	8.31 95% UI:(4.41,13.85)	6.51 95% UI:(4.42,8.56)
eGFR Stage 1, macroalbuminuria, not detected	0.69 95% UI:(0.24,1.57)	0.44 95% UI:(0.15,1.12)
eGFR Stage 2, no albuminuria, not detected	31.76 95% UI:(24.91,39.01)	28.96 95% UI:(20.25,37.76)
eGFR Stage 2, microalbuminuria, not detected	4.56 95% UI:(2.75,6.99)	4.01 95% UI:(2.29,5.88)
eGFR Stage 2, macroalbuminuria, not detected	0.91 95% UI:(0.34,1.96)	0.86 95% UI:(0.27,2.24)
eGFR Stage 3a, no albuminuria, not detected	1.21 95% UI:(0.69,1.94)	0.71 95% UI:(0.2,1.83)
eGFR Stage 3a, microalbuminuria, not detected	0.29 95% UI:(0.1,0.62)	0.21 95% UI:(0.05,0.54)
eGFR Stage 3a, macroalbuminuria, not detected	0.32 95% UI:(0.1,0.74)	0.07 95% UI:(0.01,0.2)
eGFR Stage 3b, no albuminuria, not detected	0.14 95% UI:(0.01,0.38)	0.23 95% UI:(0.07,0.59)
eGFR Stage 3b, microalbuminuria, not detected	0.08 95% UI:(0.0,0.3)	0.1 95% UI:(0.03,0.25)
eGFR Stage 3b, macroalbuminuria, not detected	0.1 95% UI:(0.01,0.29)	0.05 95% UI:(0.01,0.15)
eGFR Stage 4, no albuminuria, not detected	0.04 95% UI:(0.0,0.21)	0.05 95% UI:(0.01,0.13)
eGFR Stage 4, microalbuminuria, not detected	0.02 95% UI:(0.0,0.11)	0.03 95% UI:(0.0,0.08)
eGFR Stage 4, macroalbuminuria, not detected	0.02 95% UI:(0.0,0.09)	0.03 95% UI:(0.01,0.08)
eGFR Stage 1, microalbuminuria, detected	0.12 95% UI:(0.02,0.34)	0.06 95% UI:(0.02,0.13)
eGFR Stage 1, macroalbuminuria, detected	0.01 95% UI:(0.0,0.06)	0.01 95% UI:(0.0,0.04)
eGFR Stage 2, microalbuminuria, detected	0.22 95% UI:(0.06,0.53)	0.04 95% UI:(0.01,0.1)
eGFR Stage 2, macroalbuminuria, detected	0.05 95% UI:(0.01,0.17)	0.03 95% UI:(0.0,0.12)
eGFR Stage 3a, no albuminuria, detected	0.02 95% UI:(0.0,0.06)	0.02 95% UI:(0.01,0.06)
eGFR Stage 3a, microalbuminuria, detected	0.01 95% UI:(0.0,0.04)	0.01 95% UI:(0.0,0.03)
eGFR Stage 3a, macroalbuminuria, detected	0.02 95% UI:(0.0,0.07)	0.0 95% UI:(0.0,0.02)
eGFR Stage 3b, no albuminuria, detected	0.01 95% UI:(0.0,0.03)	0.01 95% UI:(0.0,0.04)
eGFR Stage 3b, microalbuminuria, detected	0.01 95% UI:(0.0,0.03)	0.01 95% UI:(0.0,0.02)
eGFR Stage 3b, macroalbuminuria, detected	0.01 95% UI:(0.0,0.05)	0.01 95% UI:(0.0,0.02)
eGFR Stage 4, no albuminuria, detected	0.01 95% UI:(0.0,0.07)	0.01 95% UI:(0.0,0.03)
eGFR Stage 4, microalbuminuria, detected	0.01 95% UI:(0.0,0.08)	0.01 95% UI:(0.0,0.02)
eGFR Stage 4, macroalbuminuria, detected	0.01 95% UI:(0.0,0.07)	0.01 95% UI:(0.0,0.02)
eGFR Stage 1, microalbuminuria, detected and treated	0.16 95% UI:(0.03,0.41)	0.09 95% UI:(0.03,0.18)
eGFR Stage 1, macroalbuminuria, detected and treated	0.05 95% UI:(0.01,0.16)	0.02 95% UI:(0.0,0.06)
eGFR Stage 2, microalbuminuria, detected and treated	0.14 95% UI:(0.03,0.38)	0.06 95% UI:(0.02,0.14)
eGFR Stage 2, macroalbuminuria, detected and treated	0.13 95% UI:(0.03,0.38)	0.04 95% UI:(0.01,0.16)
eGFR Stage 3a, no albuminuria, detected and treated	0.03 95% UI:(0.0,0.08)	0.07 95% UI:(0.02,0.19)
eGFR Stage 3a, microalbuminuria, detected and treated	0.02 95% UI:(0.0,0.06)	0.03 95% UI:(0.01,0.08)
eGFR Stage 3a, macroalbuminuria, detected and treated	0.06 95% UI:(0.01,0.17)	0.01 95% UI:(0.0,0.04)
eGFR Stage 3b, no albuminuria, detected and treated	0.01 95% UI:(0.0,0.04)	0.04 95% UI:(0.01,0.09)
eGFR Stage 3b, microalbuminuria, detected and treated	0.02 95% UI:(0.0,0.08)	0.02 95% UI:(0.01,0.05)
eGFR Stage 3b, macroalbuminuria, detected and treated	0.05 95% UI:(0.0,0.16)	0.01 95% UI:(0.0,0.04)
eGFR Stage 4, no albuminuria, detected and treated	0.03 95% UI:(0.0,0.15)	0.04 95% UI:(0.01,0.09)
eGFR Stage 4, microalbuminuria, detected and treated	0.03 95% UI:(0.0,0.15)	0.03 95% UI:(0.01,0.06)
eGFR Stage 4, macroalbuminuria, detected and treated	0.07 95% UI:(0.0,0.28)	0.02 95% UI:(0.01,0.06)
Kidney failure pre-kidney replacement therapy, no albuminuria	0.02 95% UI:(0.0,0.13)	0.01 95% UI:(0.0,0.04)
Kidney failure pre-kidney replacement therapy, microalbuminuria	0.0 95% UI:(0.0,0.0)	0.01 95% UI:(0.0,0.03)
Kidney failure pre-kidney replacement therapy, macroalbuminuria	0.01 95% UI:(0.0,0.06)	0.02 95% UI:(0.0,0.05)
Kidney failure on kidney replacement therapy, no albuminuria	0.04 95% UI:(0.0,0.19)	0.01 95% UI:(0.0,0.04)
Kidney failure on kidney replacement therapy, microalbuminuria	0.0 95% UI:(0.0,0.0)	0.02 95% UI:(0.0,0.06)
Kidney failure on kidney replacement therapy, macroalbuminuria	0.09 95% UI:(0.0,0.29)	0.19 95% UI:(0.04,0.44)

© 2025 Cusick MM et al. JAMA Network Open.

eTable 20. CKD Distribution Estimates and Calibration Targets (NHANES Estimates) and Calibrated Model Projections for Adults From Additional Racial and Ethnic Groups Aged 60 to 69 Years

	Target	Model
eGFR Stage 1, no albuminuria, not detected	34.17 95% UI:(27.22,41.57)	33.11 95% UI:(25.95,40.87)
eGFR Stage 1, microalbuminuria, not detected	4.95 95% UI:(3.11,7.36)	4.27 95% UI:(2.58,6.03)
eGFR Stage 1, macroalbuminuria, not detected	0.28 95% UI:(0.07,0.75)	0.15 95% UI:(0.05,0.33)
eGFR Stage 2, no albuminuria, not detected	46.93 95% UI:(40.46,53.16)	45.37 95% UI:(38.37,52.06)
eGFR Stage 2, microalbuminuria, not detected	5.87 95% UI:(4.02,8.14)	7.83 95% UI:(5.12,10.2)
eGFR Stage 2, macroalbuminuria, not detected	0.79 95% UI:(0.26,1.82)	1.1 95% UI:(0.45,2.29)
eGFR Stage 3a, no albuminuria, not detected	2.86 95% UI:(1.72,4.38)	3.46 95% UI:(2.19,5.23)
eGFR Stage 3a, microalbuminuria, not detected	0.58 95% UI:(0.25,1.13)	1.05 95% UI:(0.57,1.68)
eGFR Stage 3a, macroalbuminuria, not detected	0.44 95% UI:(0.11,1.11)	0.32 95% UI:(0.14,0.64)
eGFR Stage 3b, no albuminuria, not detected	0.37 95% UI:(0.03,0.93)	0.7 95% UI:(0.33,1.21)
eGFR Stage 3b, microalbuminuria, not detected	0.17 95% UI:(0.0,0.68)	0.33 95% UI:(0.15,0.62)
eGFR Stage 3b, macroalbuminuria, not detected	0.16 95% UI:(0.01,0.52)	0.2 95% UI:(0.08,0.39)
eGFR Stage 4, no albuminuria, not detected	0.09 95% UI:(0.0,0.44)	0.11 95% UI:(0.03,0.25)
eGFR Stage 4, microalbuminuria, not detected	0.04 95% UI:(0.0,0.22)	0.07 95% UI:(0.02,0.17)
eGFR Stage 4, macroalbuminuria, not detected	0.03 95% UI:(0.0,0.16)	0.09 95% UI:(0.03,0.21)
eGFR Stage 1, microalbuminuria, detected	0.05 95% UI:(0.01,0.15)	0.04 95% UI:(0.01,0.09)
eGFR Stage 1, macroalbuminuria, detected	0.0 95% UI:(0.0,0.02)	0.0 95% UI:(0.0,0.01)
eGFR Stage 2, microalbuminuria, detected	0.11 95% UI:(0.01,0.38)	0.07 95% UI:(0.03,0.16)
eGFR Stage 2, macroalbuminuria, detected	0.03 95% UI:(0.0,0.11)	0.03 95% UI:(0.01,0.12)
eGFR Stage 3a, no albuminuria, detected	0.02 95% UI:(0.0,0.06)	0.04 95% UI:(0.01,0.11)
eGFR Stage 3a, microalbuminuria, detected	0.01 95% UI:(0.0,0.04)	0.03 95% UI:(0.01,0.07)
eGFR Stage 3a, macroalbuminuria, detected	0.01 95% UI:(0.0,0.04)	0.01 95% UI:(0.0,0.04)
eGFR Stage 3b, no albuminuria, detected	0.01 95% UI:(0.0,0.03)	0.03 95% UI:(0.01,0.08)
eGFR Stage 3b, microalbuminuria, detected	0.01 95% UI:(0.0,0.04)	0.02 95% UI:(0.01,0.05)
eGFR Stage 3b, macroalbuminuria, detected	0.01 95% UI:(0.0,0.05)	0.01 95% UI:(0.0,0.03)
eGFR Stage 4, no albuminuria, detected	0.01 95% UI:(0.0,0.08)	0.02 95% UI:(0.01,0.06)
eGFR Stage 4, microalbuminuria, detected	0.01 95% UI:(0.0,0.07)	0.02 95% UI:(0.01,0.05)
eGFR Stage 4, macroalbuminuria, detected	0.01 95% UI:(0.0,0.08)	0.02 95% UI:(0.0,0.04)
eGFR Stage 1, microalbuminuria, detected and treated	0.15 95% UI:(0.04,0.34)	0.07 95% UI:(0.02,0.13)
eGFR Stage 1, macroalbuminuria, detected and treated	0.03 95% UI:(0.0,0.09)	0.01 95% UI:(0.0,0.02)
eGFR Stage 2, microalbuminuria, detected and treated	0.46 95% UI:(0.12,1.17)	0.13 95% UI:(0.06,0.24)
eGFR Stage 2, macroalbuminuria, detected and treated	0.16 95% UI:(0.03,0.45)	0.05 95% UI:(0.01,0.16)
eGFR Stage 3a, no albuminuria, detected and treated	0.12 95% UI:(0.03,0.3)	0.22 95% UI:(0.1,0.44)
eGFR Stage 3a, microalbuminuria, detected and treated	0.06 95% UI:(0.01,0.19)	0.1 95% UI:(0.04,0.2)
eGFR Stage 3a, macroalbuminuria, detected and treated	0.12 95% UI:(0.02,0.35)	0.03 95% UI:(0.01,0.08)
eGFR Stage 3b, no albuminuria, detected and treated	0.04 95% UI:(0.0,0.18)	0.09 95% UI:(0.03,0.21)
eGFR Stage 3b, microalbuminuria, detected and treated	0.05 95% UI:(0.0,0.25)	0.06 95% UI:(0.02,0.12)
eGFR Stage 3b, macroalbuminuria, detected and treated	0.11 95% UI:(0.01,0.36)	0.03 95% UI:(0.01,0.08)
eGFR Stage 4, no albuminuria, detected and treated	0.09 95% UI:(0.0,0.44)	0.08 95% UI:(0.02,0.18)
eGFR Stage 4, microalbuminuria, detected and treated	0.09 95% UI:(0.0,0.47)	0.06 95% UI:(0.02,0.14)
eGFR Stage 4, macroalbuminuria, detected and treated	0.15 95% UI:(0.0,0.65)	0.06 95% UI:(0.02,0.14)
Kidney failure pre-kidney replacement therapy, no albuminuria	0.05 95% UI:(0.0,0.28)	0.02 95% UI:(0.0,0.07)
Kidney failure pre-kidney replacement therapy, microalbuminuria	0.0 95% UI:(0.0,0.0)	0.03 95% UI:(0.0,0.07)
Kidney failure pre-kidney replacement therapy, macroalbuminuria	0.02 95% UI:(0.0,0.11)	0.04 95% UI:(0.01,0.11)
Kidney failure on kidney replacement therapy, no albuminuria	0.1 95% UI:(0.0,0.44)	0.02 95% UI:(0.0,0.07)
Kidney failure on kidney replacement therapy, microalbuminuria	0.0 95% UI:(0.0,0.0)	0.04 95% UI:(0.0,0.14)
Kidney failure on kidney replacement therapy, macroalbuminuria	0.19 95% UI:(0.0,0.63)	0.37 95% UI:(0.09,0.86)

© 2025 Cusick MM et al. JAMA Network Open.

eTable 21. CKD Distribution Estimates and Calibration Targets (NHANES Estimates) and Calibrated Model Projections for Adults From Additional Racial and Ethnic Groups Aged 70 to 79 Years

	Target	Model
eGFR Stage 1, no albuminuria, not detected	7.83 95% UI:(5.46,10.78)	9.19 95% UI:(5.32,13.95)
eGFR Stage 1, microalbuminuria, not detected	1.55 95% UI:(0.83,2.6)	1.1 95% UI:(0.36,2.22)
eGFR Stage 1, macroalbuminuria, not detected	0.09 95% UI:(0.02,0.31)	0.01 95% UI:(0.0,0.04)
eGFR Stage 2, no albuminuria, not detected	51.13 95% UI:(45.59,56.49)	53.56 95% UI:(45.95,60.86)
eGFR Stage 2, microalbuminuria, not detected	8.76 95% UI:(6.01,12.17)	9.82 95% UI:(6.64,12.75)
eGFR Stage 2, macroalbuminuria, not detected	1.24 95% UI:(0.31,3.32)	0.69 95% UI:(0.26,1.43)
eGFR Stage 3a, no albuminuria, not detected	11.78 95% UI:(8.03,16.09)	10.84 95% UI:(7.02,14.99)
eGFR Stage 3a, microalbuminuria, not detected	3.2 95% UI:(1.52,5.67)	3.68 95% UI:(2.27,5.44)
eGFR Stage 3a, macroalbuminuria, not detected	2.18 95% UI:(0.81,4.51)	0.72 95% UI:(0.31,1.4)
eGFR Stage 3b, no albuminuria, not detected	1.83 95% UI:(0.15,4.66)	2.67 95% UI:(1.29,4.21)
eGFR Stage 3b, microalbuminuria, not detected	1.15 95% UI:(0.03,4.1)	1.35 95% UI:(0.69,2.14)
eGFR Stage 3b, macroalbuminuria, not detected	1.21 95% UI:(0.13,3.41)	0.57 95% UI:(0.3,0.92)
eGFR Stage 4, no albuminuria, not detected	0.54 95% UI:(0.0,2.48)	0.44 95% UI:(0.09,1.03)
eGFR Stage 4, microalbuminuria, not detected	0.25 95% UI:(0.0,1.3)	0.31 95% UI:(0.06,0.76)
eGFR Stage 4, macroalbuminuria, not detected	0.35 95% UI:(0.0,1.78)	0.3 95% UI:(0.09,0.67)
eGFR Stage 1, microalbuminuria, detected	0.01 95% UI:(0.0,0.04)	0.01 95% UI:(0.0,0.03)
eGFR Stage 1, macroalbuminuria, detected	0.0 95% UI:(0.0,0.01)	0.0 95% UI:(0.0,0.0)
eGFR Stage 2, microalbuminuria, detected	0.16 95% UI:(0.03,0.49)	0.1 95% UI:(0.04,0.2)
eGFR Stage 2, macroalbuminuria, detected	0.04 95% UI:(0.0,0.16)	0.02 95% UI:(0.0,0.05)
eGFR Stage 3a, no albuminuria, detected	0.1 95% UI:(0.0,0.48)	0.16 95% UI:(0.06,0.36)
eGFR Stage 3a, microalbuminuria, detected	0.04 95% UI:(0.0,0.17)	0.1 95% UI:(0.04,0.22)
eGFR Stage 3a, macroalbuminuria, detected	0.05 95% UI:(0.0,0.18)	0.03 95% UI:(0.01,0.08)
eGFR Stage 3b, no albuminuria, detected	0.03 95% UI:(0.0,0.15)	0.09 95% UI:(0.03,0.2)
eGFR Stage 3b, microalbuminuria, detected	0.05 95% UI:(0.0,0.3)	0.07 95% UI:(0.02,0.14)
eGFR Stage 3b, macroalbuminuria, detected	0.08 95% UI:(0.0,0.38)	0.03 95% UI:(0.01,0.09)
eGFR Stage 4, no albuminuria, detected	0.07 95% UI:(0.0,0.45)	0.06 95% UI:(0.02,0.14)
eGFR Stage 4, microalbuminuria, detected	0.07 95% UI:(0.0,0.41)	0.05 95% UI:(0.02,0.12)
eGFR Stage 4, macroalbuminuria, detected	0.2 95% UI:(0.0,1.21)	0.05 95% UI:(0.01,0.13)
eGFR Stage 1, microalbuminuria, detected and treated	0.03 95% UI:(0.01,0.1)	0.02 95% UI:(0.01,0.06)
eGFR Stage 1, macroalbuminuria, detected and treated	0.0 95% UI:(0.0,0.02)	0.0 95% UI:(0.0,0.0)
eGFR Stage 2, microalbuminuria, detected and treated	0.53 95% UI:(0.15,1.28)	0.18 95% UI:(0.08,0.3)
eGFR Stage 2, macroalbuminuria, detected and treated	0.16 95% UI:(0.03,0.51)	0.03 95% UI:(0.01,0.1)
eGFR Stage 3a, no albuminuria, detected and treated	0.17 95% UI:(0.01,0.73)	0.84 95% UI:(0.45,1.47)
eGFR Stage 3a, microalbuminuria, detected and treated	0.29 95% UI:(0.05,0.85)	0.39 95% UI:(0.2,0.67)
eGFR Stage 3a, macroalbuminuria, detected and treated	0.43 95% UI:(0.09,1.16)	0.1 95% UI:(0.03,0.23)
eGFR Stage 3b, no albuminuria, detected and treated	0.19 95% UI:(0.01,0.79)	0.34 95% UI:(0.14,0.62)
eGFR Stage 3b, microalbuminuria, detected and treated	0.18 95% UI:(0.0,0.96)	0.21 95% UI:(0.1,0.37)
eGFR Stage 3b, macroalbuminuria, detected and treated	0.36 95% UI:(0.02,1.38)	0.1 95% UI:(0.04,0.2)
eGFR Stage 4, no albuminuria, detected and treated	0.38 95% UI:(0.0,1.84)	0.25 95% UI:(0.07,0.57)
eGFR Stage 4, microalbuminuria, detected and treated	0.51 95% UI:(0.0,2.29)	0.2 95% UI:(0.07,0.45)
eGFR Stage 4, macroalbuminuria, detected and treated	0.57 95% UI:(0.0,2.46)	0.18 95% UI:(0.05,0.46)
Kidney failure pre-kidney replacement therapy, no albuminuria	0.32 95% UI:(0.0,1.69)	0.07 95% UI:(0.01,0.17)
Kidney failure pre-kidney replacement therapy, microalbuminuria	0.0 95% UI:(0.0,0.0)	0.08 95% UI:(0.02,0.2)
Kidney failure pre-kidney replacement therapy, macroalbuminuria	0.26 95% UI:(0.0,1.18)	0.12 95% UI:(0.04,0.3)
Kidney failure on kidney replacement therapy, no albuminuria	0.56 95% UI:(0.0,2.55)	0.04 95% UI:(0.01,0.14)
Kidney failure on kidney replacement therapy, microalbuminuria	0.0 95% UI:(0.0,0.0)	0.09 95% UI:(0.01,0.25)
Kidney failure on kidney replacement therapy, macroalbuminuria	1.07 95% UI:(0.0,3.44)	0.74 95% UI:(0.22,1.58)

© 2025 Cusick MM et al. JAMA Network Open.

eFigure 1. Calibrated eGFR Stage Marginal Distribution Ribbon Plot for a) Hispanic Adults, b) Non-Hispanic Black Adults, c) Non-Hispanic White Adults, and d) Adults From Additional Racial and Ethnic Groups

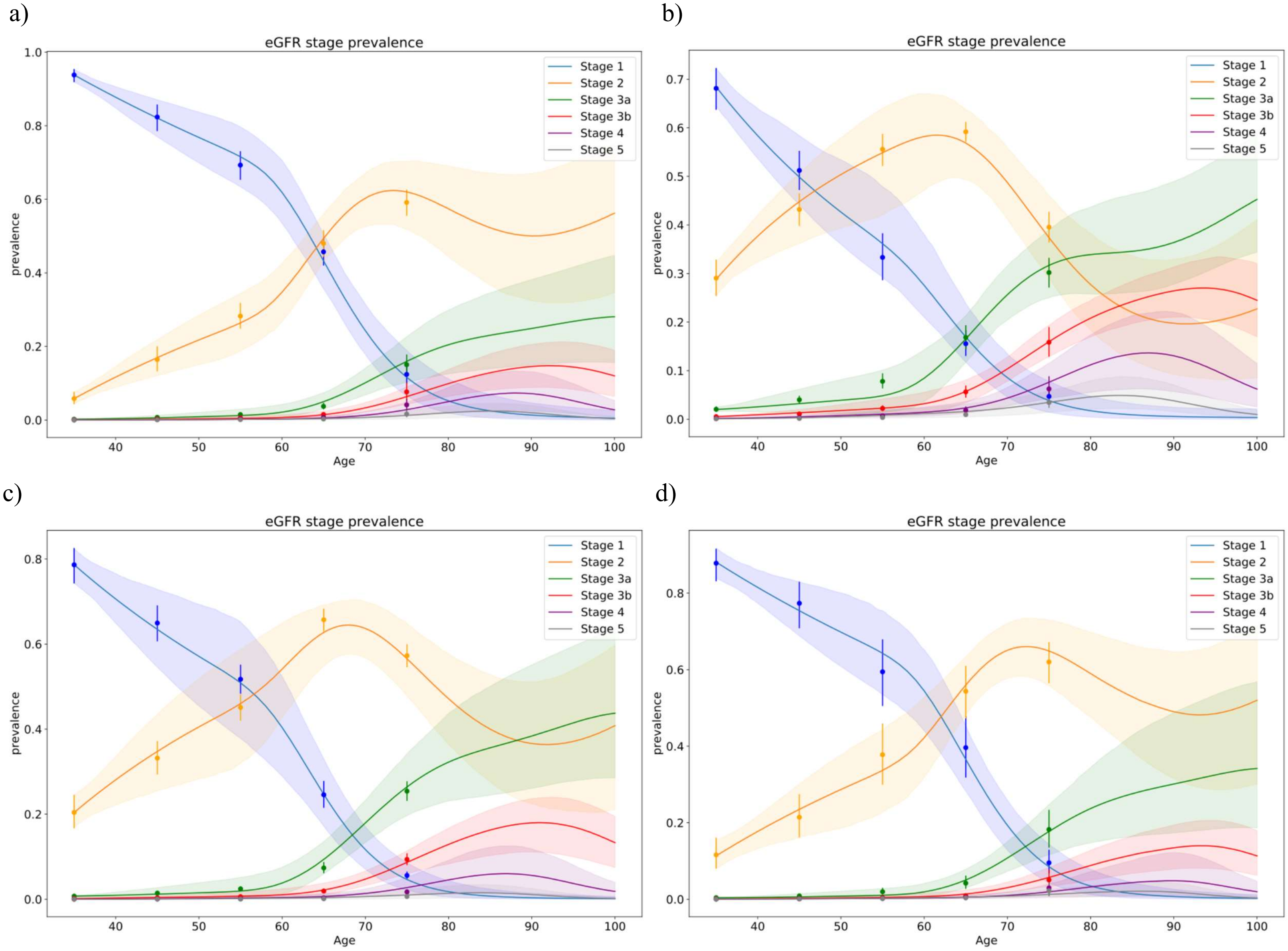

© 2025 Cusick MM et al. JAMA Network Open.

eFigure 2. Calibrated Stage 5 (eGFR <15) and Kidney Failure Receiving Kidney Replacement Therapy Marginal Distribution Ribbon Plot for a) Hispanic Adults, b) Non-Hispanic Black Adults, c) Non-Hispanic White Adults, and d) Adults From Additional Racial and Ethnic Groups

a)

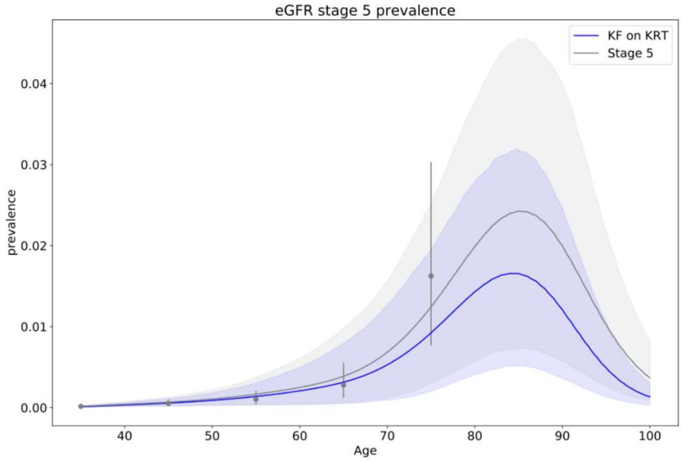

b)

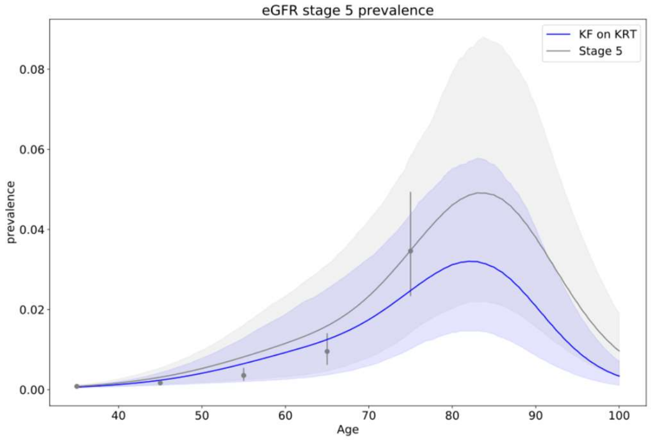

c)

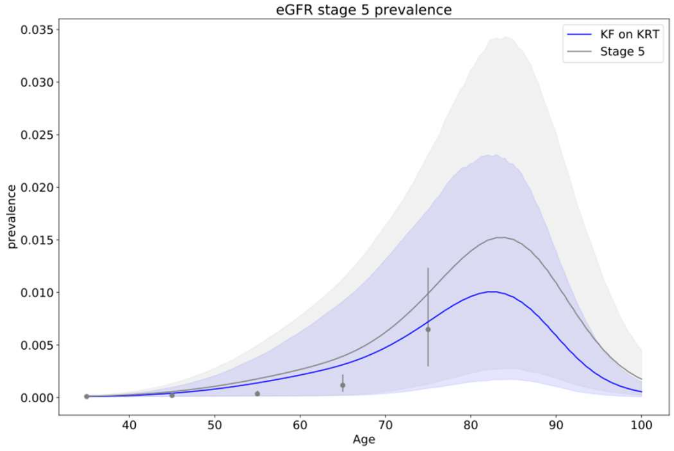

d)

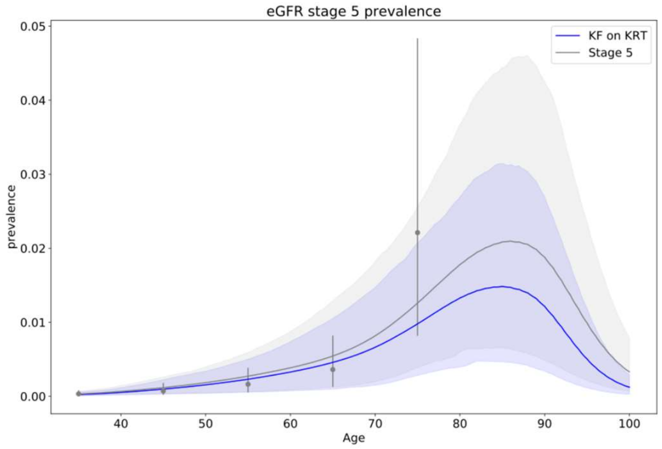

© 2025 Cusick MM et al. JAMA Network Open.

eFigure 3. Calibrated Albuminuria Stage Prevalence Marginal Distribution Ribbon plot for a) Hispanic Adults, b) Non-Hispanic Black Adults, c) Non-Hispanic White Adults, and d) Adults From Additional Racial and Ethnic Groups

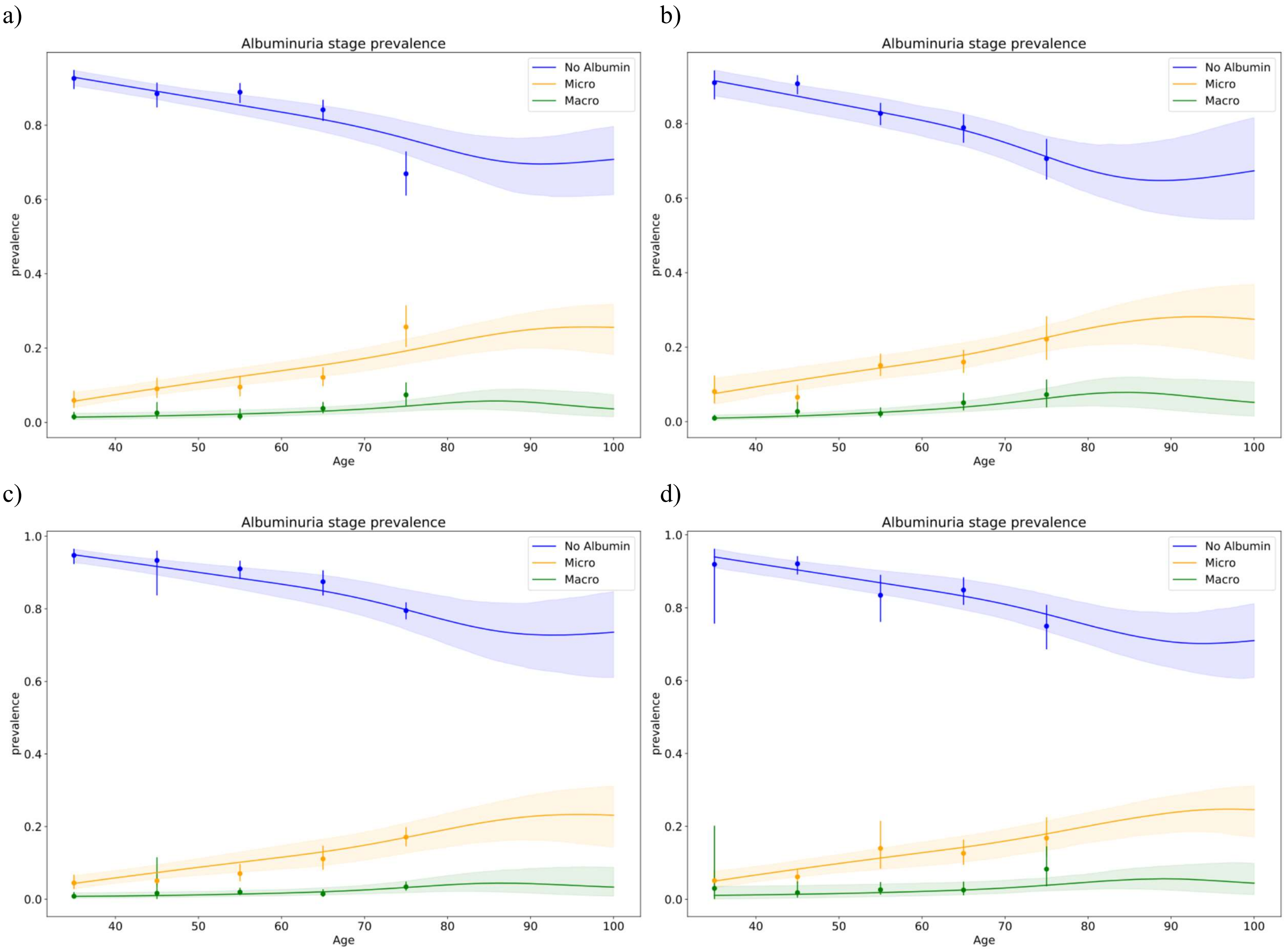

© 2025 Cusick MM et al. JAMA Network Open.

eFigure 4. Calibrated CKD Detection/Awareness Prevalence Marginal Distribution Ribbon Plot for a) Hispanic Adults, b) Non-Hispanic Black Adults, c) Non-Hispanic White Adults, and d) Adults From Additional Racial and Ethnic Groups

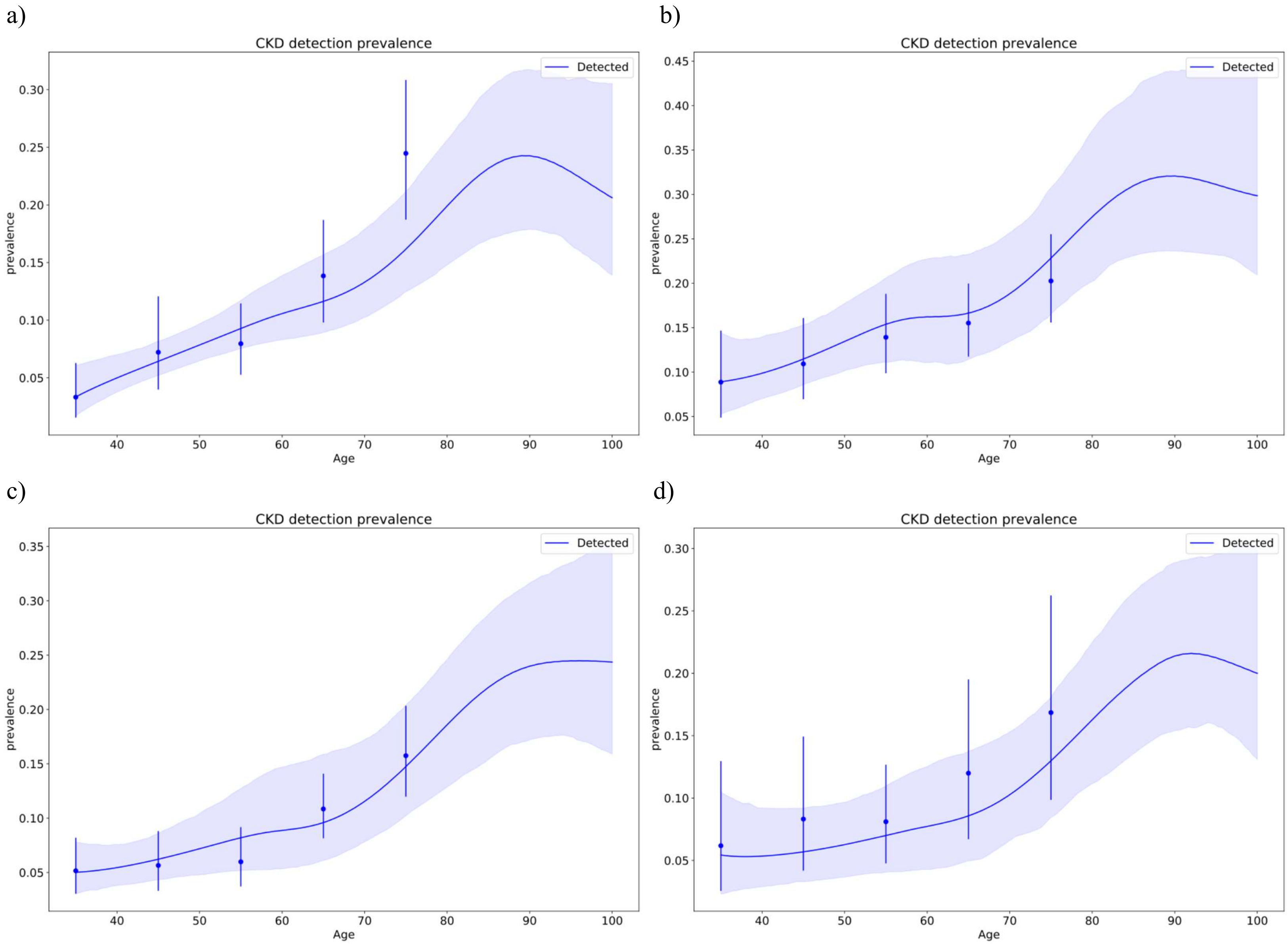

© 2025 Cusick MM et al. JAMA Network Open.

eFigure 5. Calibrated CKD Detection and Treatment Marginal Distribution Ribbon Plot for a) Hispanic Adults, b) Non-Hispanic Black Adults, c) Non-Hispanic White Adults, and d) Adults From Additional Racial and Ethnic Groups

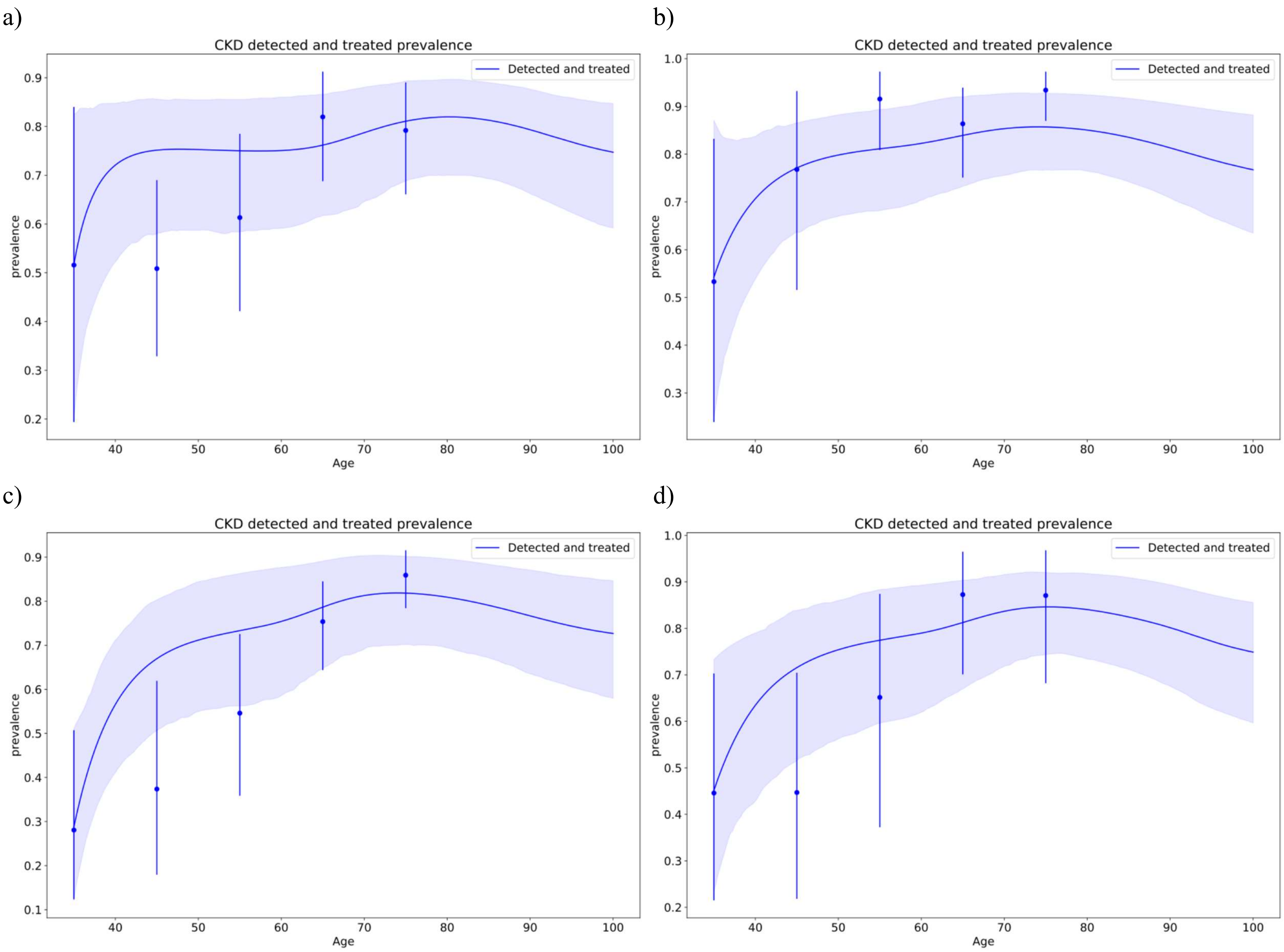

© 2025 Cusick MM et al. JAMA Network Open.

eTable 22. Projected Cases of Kidney Failure (KF) Requiring Kidney Replacement Therapy (KRT) in the US Population by Racial and Ethnic Group

Racial/ethnic group	Strategy	Cases of KF requiring KRT	Averted cases	Averted cases per 10,000 people screened	% reduction
Hispanic	Status quo with ACEi	846,000			
	Screen q10y 55-75y with SGLT2i	709,000	136,000	57	16%
	Screen q5y 55-75y with SGLT2i	699,000	147,000	62	17%
	Screen q5y 45-75y with SGLT2i	695,000	150,000	63	17%
	Screen q5y 35-75y	694,000	151,000	63	18%
Non-Hispanic Black	Status quo with ACEi	1,133,000	---		---
	Screen q10y 55-75y with SGLT2i	1,018,000	115,000	61	10%
	Screen q5y 55-75y with SGLT2i	1,006,000	127,000	68	11%
	Screen q5y 45-75y with SGLT2i	999,000	134,000	72	12%
	Screen q5y 35-75y	997,000	136,000	73	12%
Non-Hispanic white	Status quo with ACEi	2,317,000	---		---
	Screen q10y 55-75y with SGLT2i	2,004,000	313,000	30	14%
	Screen q5y 55-75y with SGLT2i	1,974,000	342,000	33	15%
	Screen q5y 45-75y with SGLT2i	1,966,000	351,000	34	15%
	Screen q5y 35-75y	1,964,000	353,000	34	15%
Additional groups	Status quo with ACEi	399,000			
	Screen q10y 55-75y with SGLT2i	331,000	68,000	54	17%
	Screen q5y 55-75y with SGLT2i	326,000	73,000	58	18%
	Screen q5y 45-75y with SGLT2i	329,000	75,000	59	18%
	Screen q5y 35-75y	324,000	76,000	60	19%

Screening every 10 years (q10y) or every 5 years (q5y)

With ACEi: with conventional CKD therapy comprising of angiotensin-converting enzyme (ACE) inhibitors or angiotensin receptor blocker (ARB) therapy

With SGLT2i: With the addition of sodium–glucose cotransporter-2 (SGLT2) inhibitors to conventional CKD therapy

Additional racial and ethnic groups includes those self-identifying as Non-Hispanic Asian or Other Race (including multi-racial).

© 2025 Cusick MM et al. JAMA Network Open.

eTable 23. Cost-Effectiveness Table for the 35-Year-Old Cohort Across Racial and Ethnic Groups

Racial/ethnic group	Strategy	Cost	Effect	Incremental Cost	Incremental QALYs	ICER
Hispanic	Status quo with ACEi	256200	20.1			
	Status quo with SGLT2i	257000	20.12	800	0.02	46100
	Screen 65y with SGLT2i	259200	20.16	2200	0.04	54300
	Screen q10y 55-65y with SGLT2i	260200	20.17	900	0.01	69000
	Screen q10y 55-75y with SGLT2i	260600	20.18	500	0.01	72000
	Screen q5y 55-75y with SGLT2i	261200	20.18	500	0.01	102600
	Screen q5y 45-75y with SGLT2i	262200	20.19	1000	0.01	194700
	Screen q5y 35-75y with SGLT2i	263200	20.19	1000	0	235200
Non-Hispanic Black	Status quo with ACEi	232100	17.57			
	Status quo with SGLT2i	233500	17.61	1400	0.04	37400
	Screen 55y with SGLT2i	236400	17.67	2900	0.06	49200
	Screen q10y 55-65y with SGLT2i	237400	17.69	900	0.02	59100
	Screen q10y 55-75y with SGLT2i	237800	17.69	400	0.01	68800
	Screen q5y 55-75y with SGLT2i	238500	17.7	700	0.01	73400
	Screen q10y 45-75y with SGLT2i	239100	17.71	600	0.01	75800
	Screen q10y 35-75y with SGLT2i	240100	17.72	1000	0.01	83700
	Screen q5y 35-75y with SGLT2i	241300	17.73	1300	0.01	115000
Non-Hispanic white	Status quo with ACEi	239900	18.97			
	Status quo with SGLT2i	240600	18.99	700	0.02	45700
	Screen 65y with SGLT2i	242800	19.03	2200	0.04	58500
	Screen q10y 65-75y with SGLT2i	243400	19.04	600	0.01	69800
	Screen q10y 55-75y with SGLT2i	244400	19.05	1000	0.01	77900
	Screen q5y 55-75y with SGLT2i	245000	19.05	600	0.01	105700
	Screen q5y 45-75y with SGLT2i	246100	19.06	1200	0.01	190000
	Screen q5y 35-75y with SGLT2i	247200	19.06	1100	0	247800
Additional groups	Status quo with ACEi	260400	20.06			
	Status quo with SGLT2i	261000	20.07	500	0.01	37900
	Screen 65y with SGLT2i	263100	20.11	2100	0.04	55700
	Screen q10y 65-75y with SGLT2i	263700	20.12	600	0.01	69200
	Screen q10y 55-75y with SGLT2i	264500	20.13	800	0.01	76300
	Screen q5y 55-75y with SGLT2i	265000	20.13	500	0	110500
	Screen q5y 45-75y with SGLT2i	266000	20.14	1000	0.01	194900
	Screen q5y 35-75y with SGLT2i	267000	20.14	1000	0	220800
Overall	Status quo with ACEi	244300	19.14			
	Status quo with SGLT2i	245200	19.16	800	0.02	43100
	Screen 65y with SGLT2i	247400	19.2	2200	0.04	56200
	Screen q10y 55-65y with SGLT2i	248500	19.21	1100	0.02	68700

© 2025 Cusick MM et al. JAMA Network Open.

	Screen q10y 55-75y with SGLT2i	248900	19.22	500	0.01	71500
	Screen q5y 55-75y with SGLT2i	249500	19.22	600	0.01	99100
	Screen q5y 45-75y with SGLT2i	250700	19.23	1200	0.01	158700
	Screen q5y 35-75y with SGLT2i	251800	19.24	1100	0.01	200700

Screening every 10 years (q10y) or every 5 years (q5y)

With ACEi: with conventional CKD therapy comprising of angiotensin-converting enzyme (ACE) inhibitors or angiotensin receptor blocker (ARB) therapy

With SGLT2i: With the addition of sodium–glucose cotransporter-2 (SGLT2) inhibitors to conventional CKD therapy

Additional racial and ethnic groups includes those self-identifying as Non-Hispanic Asian or Other Race (including multi-racial).

© 2025 Cusick MM et al. JAMA Network Open.

eTable 24. Cost-Effectiveness Table for the 45-Year-Old Cohort Across Racial and Ethnic Groups

Racial/ethnic group	Strategy	Cost	Effect	Incremental Cost	Incremental QALYs	ICER
Hispanic	Status quo with ACEi	283100	17.05			
	Status quo with SGLT2i	284200	17.07	1200	0.02	47000
	Screen 65y with SGLT2i	287200	17.12	3000	0.05	54600
	Screen q10y 55-65y with SGLT2i	288500	17.14	1300	0.02	69000
	Screen q10y 55-75y with SGLT2i	289100	17.15	700	0.01	72000
	Screen q5y 55-75y with SGLT2i	289800	17.16	700	0.01	102600
	Screen q5y 45-75y with SGLT2i	291200	17.17	1300	0.01	186400
Non-Hispanic Black	Status quo with ACEi	253200	14.42			
	Status quo with SGLT2i	255200	14.47	2000	0.05	38900
	Screen 55y with SGLT2i	259200	14.55	4100	0.08	49700
	Screen q10y 55-65y with SGLT2i	260500	14.57	1300	0.02	59100
	Screen q10y 55-75y with SGLT2i	261200	14.58	600	0.01	68800
	Screen q10y 45-75y with SGLT2i	262800	14.61	1600	0.02	71500
	Screen q5y 45-75y with SGLT2i	264100	14.62	1300	0.01	93800
Non-Hispanic white	Status quo with ACEi	262800	15.92			
	Status quo with SGLT2i	263900	15.94	1000	0.02	46100
	Screen 65y with SGLT2i	266800	15.99	3000	0.05	58900
	Screen q10y 65-75y with SGLT2i	267700	16	800	0.01	69800
	Screen q10y 55-75y with SGLT2i	269000	16.02	1300	0.02	77900
	Screen q5y 55-75y with SGLT2i	269800	16.03	800	0.01	105700
	Screen q5y 45-75y with SGLT2i	271300	16.03	1500	0.01	183000
Additional groups	Status quo with ACEi	287700	17.03			
	Status quo with SGLT2i	288400	17.05	700	0.02	39200
	Screen 65y with SGLT2i	291300	17.1	2900	0.05	56100
	Screen q10y 65-75y with SGLT2i	292200	17.11	900	0.01	69200
	Screen q10y 55-75y with SGLT2i	293200	17.13	1000	0.01	76300
	Screen q5y 55-75y with SGLT2i	293900	17.13	700	0.01	110500
	Screen q5y 45-75y with SGLT2i	295200	17.14	1300	0.01	186700
Overall	Status quo with ACEi	267700	16.04			
	Status quo with SGLT2i	268800	16.07	1100	0.03	44000
	Screen 65y with SGLT2i	271800	16.12	3000	0.05	56800
	Screen q10y 55-65y with SGLT2i	273300	16.14	1500	0.02	68800
	Screen q10y 55-75y with SGLT2i	274000	16.15	600	0.01	71500
	Screen q5y 55-75y with SGLT2i	274800	16.16	800	0.01	99100
	Screen q5y 45-75y with SGLT2i	276300	16.17	1500	0.01	152800

Screening every 10 years (q10y) or every 5 years (q5y)

© 2025 Cusick MM et al. JAMA Network Open.

With ACEi: with conventional CKD therapy comprising of angiotensin-converting enzyme (ACE) inhibitors or angiotensin receptor blocker (ARB) therapy

With SGLT2i: With the addition of sodium–glucose cotransporter-2 (SGLT2) inhibitors to conventional CKD therapy

Additional racial and ethnic groups includes those self-identifying as Non-Hispanic Asian or Other Race (including multi-racial).

© 2025 Cusick MM et al. JAMA Network Open.

eTable 25. Cost-Effectiveness Table for the 55-Year-Old Cohort Across Racial and Ethnic Groups

Racial/ethnic group	Strategy	Cost	Effect	Incremental Cost	Incremental QALYs	ICER
Hispanic	Status quo with ACEi	293200	13.62			
	Status quo with SGLT2i	294800	13.65	1600	0.03	48700
	Screen 65y with SGLT2i	298900	13.73	4100	0.08	54600
	Screen q10y 55-65y with SGLT2i	300600	13.76	1700	0.03	66400
	Screen q10y 55-75y with SGLT2i	301500	13.77	900	0.01	72000
	Screen q5y 55-75y with SGLT2i	302500	13.78	1000	0.01	102400
Non-Hispanic Black	Status quo with ACEi	256700	11.06			
	Status quo with SGLT2i	259600	11.12	2900	0.07	44600
	Screen 55y with SGLT2i	265300	11.24	5700	0.12	49000
	Screen q10y 55-65y with SGLT2i	267200	11.27	1800	0.03	59100
	Screen q10y 55-75y with SGLT2i	268100	11.28	900	0.01	68800
	Screen q5y 55-75y with SGLT2i	269400	11.3	1300	0.02	73400
Non-Hispanic white	Status quo with ACEi	268900	12.56			
	Status quo with SGLT2i	270300	12.59	1400	0.03	47900
	Screen 65y with SGLT2i	274500	12.66	4200	0.07	58900
	Screen q10y 65-75y with SGLT2i	275700	12.68	1200	0.02	69700
	Screen q10y 55-75y with SGLT2i	277500	12.7	1800	0.02	75000
	Screen q5y 55-75y with SGLT2i	278600	12.71	1100	0.01	105500
Additional groups	Status quo with ACEi	297100	13.63			
	Status quo with SGLT2i	298100	13.65	1100	0.03	43100
	Screen 65y with SGLT2i	302100	13.72	4000	0.07	56100
	Screen q10y 65-75y with SGLT2i	303300	13.74	1200	0.02	69100
	Screen q10y 55-75y with SGLT2i	304700	13.76	1300	0.02	72600
	Screen q5y 55-75y with SGLT2i	305600	13.77	1000	0.01	110400
Overall	Status quo with ACEi	272600	12.6			
	Status quo with SGLT2i	274200	12.63	1600	0.03	47000
	Screen 65y with SGLT2i	278400	12.7	4200	0.07	57100
	Screen q10y 55-65y with SGLT2i	280500	12.73	2100	0.03	67200
	Screen q10y 55-75y with SGLT2i	281400	12.75	900	0.01	71500
	Screen q5y 55-75y with SGLT2i	282500	12.76	1100	0.01	99300

Screening every 10 years (q10y) or every 5 years (q5y)

With ACEi: with conventional CKD therapy comprising of angiotensin-converting enzyme (ACE) inhibitors or angiotensin receptor blocker (ARB) therapy

With SGLT2i: With the addition of sodium–glucose cotransporter-2 (SGLT2) inhibitors to conventional CKD therapy

© 2025 Cusick MM et al. JAMA Network Open.

Additional racial and ethnic groups includes those self-identifying as Non-Hispanic Asian or Other Race (including multi-racial).

© 2025 Cusick MM et al. JAMA Network Open.

eTable 26. Cost-Effectiveness Table for the 65-Year-Old Cohort Across Racial and Ethnic Groups

Racial/ethnic group	Strategy	Cost	Effect	Incremental Cost	Incremental QALYs	ICER
Hispanic	Status quo with ACEi	272900	9.94			
	Status quo with SGLT2i	275100	9.98	2300	0.04	51600
	Screen 65y with SGLT2i	281000	10.09	5900	0.11	53800
	Screen q10y 65-75y with SGLT2i	282800	10.12	1800	0.03	69400
	Screen q5y 65-75y with SGLT2i	283600	10.13	900	0.01	79100
Non-Hispanic Black	Status quo with ACEi	233300	7.74			
	Screen 65y with SGLT2i	244700	7.96	11400	0.22	52000
	Screen q10y 65-75y with SGLT2i	246500	7.99	1800	0.03	67300
	Screen q5y 65-75y with SGLT2i	247600	8	1200	0.02	68300
Non-Hispanic white	Status quo with ACEi	245200	9.05			
	Status quo with SGLT2i	247200	9.09	2000	0.04	51600
	Screen 65y with SGLT2i	253300	9.19	6100	0.1	58100
	Screen q10y 65-75y with SGLT2i	255100	9.22	1700	0.03	69600
	Screen q5y 65-75y with SGLT2i	256000	9.23	1000	0.01	83400
Additional groups	Status quo with ACEi	271800	9.86			
	Status quo with SGLT2i	273400	9.89	1600	0.03	48500
	Screen 65y with SGLT2i	279000	9.99	5600	0.1	55300
	Screen q10y 65-75y with SGLT2i	280700	10.01	1700	0.02	69000
	Screen q5y 65-75y with SGLT2i	281500	10.02	800	0.01	83600
Overall	Status quo with ACEi	248300	9.05			
	Status quo with SGLT2i	250600	9.09	2300	0.04	51900
	Screen 65y with SGLT2i	256700	9.2	6100	0.11	56600
	Screen q10y 65-75y with SGLT2i	258400	9.23	1700	0.03	69300
	Screen q5y 65-75y with SGLT2i	259400	9.24	1000	0.01	80800

Screening every 10 years (q10y) or every 5 years (q5y)

With ACEi: with conventional CKD therapy comprising of angiotensin-converting enzyme (ACE) inhibitors or angiotensin receptor blocker (ARB) therapy

With SGLT2i: With the addition of sodium–glucose cotransporter-2 (SGLT2) inhibitors to conventional CKD therapy

Additional racial and ethnic groups includes those self-identifying as Non-Hispanic Asian or Other Race (including multi-racial).

© 2025 Cusick MM et al. JAMA Network Open.

eTable 27. Cost-Effectiveness Table for the 75-Year-Old Cohort Across Racial and Ethnic Groups

Racial/ethnic group	Strategy	Cost	Effect	Incremental Cost	Incremental QALYs	ICER
Hispanic	Status quo with ACEi	214600	6.39			
	Status quo with SGLT2i	217700	6.45	3100	0.05	59100
	Screen 75y with SGLT2i	225000	6.57	7300	0.12	60800
Non-Hispanic Black	Status quo with ACEi	179000	4.8			
	Screen 75y with SGLT2i	192400	5.01	13400	0.21	63800
Non-Hispanic white	Status quo with ACEi	184700	5.67			
	Status quo with SGLT2i	187600	5.72	2800	0.05	58600
	Screen 75y with SGLT2i	194700	5.83	7200	0.12	61500
Additional groups	Status quo with ACEi	204300	6.13			
	Status quo with SGLT2i	206600	6.17	2300	0.04	58400
	Screen 75y with SGLT2i	213600	6.29	7000	0.12	60400
Overall	Status quo with ACEi	187800	5.68			
	Status quo with SGLT2i	190900	5.73	3100	0.05	60000
	Screen 75y with SGLT2i	198100	5.85	7200	0.12	61400

Screening every 10 years (q10y) or every 5 years (q5y)

With ACEi: with conventional CKD therapy comprising of angiotensin-converting enzyme (ACE) inhibitors or angiotensin receptor blocker (ARB) therapy

With SGLT2i: With the addition of sodium–glucose cotransporter-2 (SGLT2) inhibitors to conventional CKD therapy

Additional racial and ethnic groups includes those self-identifying as Non-Hispanic Asian or Other Race (including multi-racial).

© 2025 Cusick MM et al. JAMA Network Open.

eFigure 6. Univariate Sensitivity Analysis for Every 5 Years Screening From Ages 55 to 75 Years With SGLT2i for a) Hispanic Adults, b) Non-Hispanic Black Adults, c) Non-Hispanic White Adults, d) Adults From Additional Racial and Ethnic Groups, and e) the Overall Population Aged 35 Years

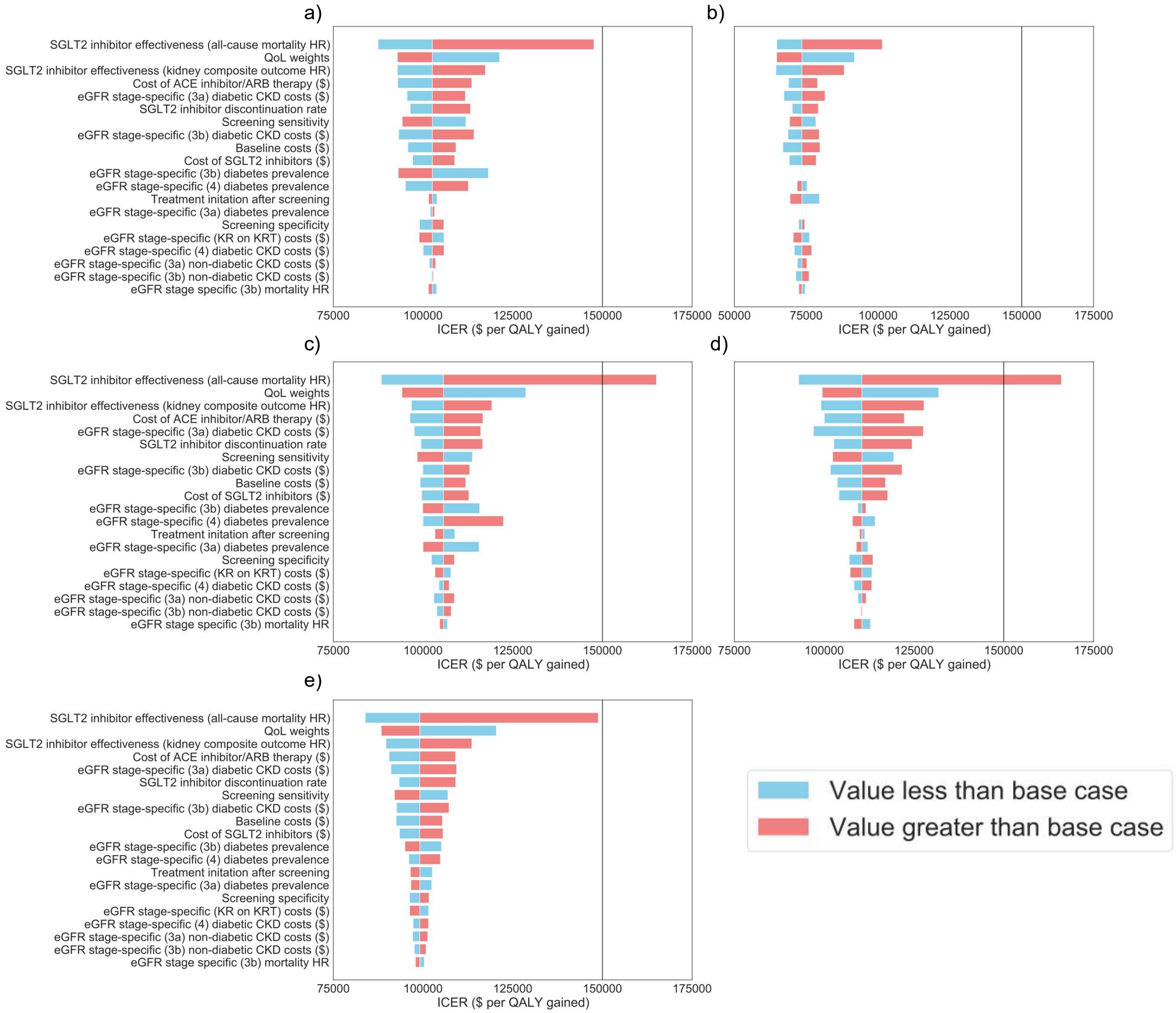

q5y: every 5 years; With SGLT2i: With the addition of sodium–glucose cotransporter-2 (SGLT2) inhibitors to conventional CKD therapy (angiotensin-converting enzyme [ACE] inhibitors or angiotensin receptor blocker [ARB] therapy

© 2025 Cusick MM et al. JAMA Network Open.

eFigure 7. Univariate Sensitivity Analysis for Every 5 Years Screening From Ages 45 to 75 Years With SGLT2i for a) Hispanic Adults, b) Non-Hispanic Black Adults, c) Non-Hispanic White Adults, d) Adults From Additional Racial and Ethnic Groups, and e) the Overall Population Aged 35 Years

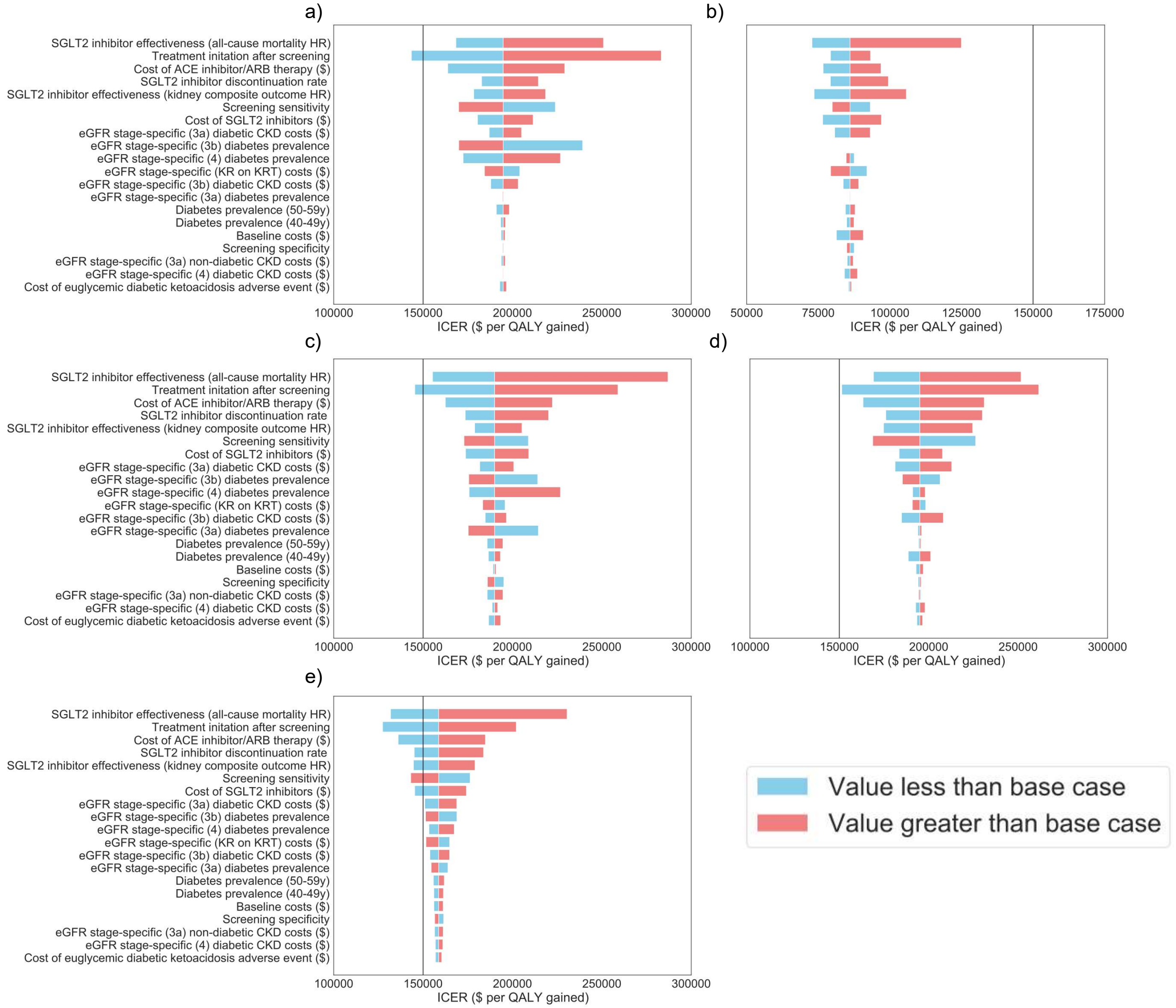

q5y: every 5 years; With SGLT2i: With the addition of sodium–glucose cotransporter-2 (SGLT2) inhibitors to conventional CKD therapy (angiotensin-converting enzyme [ACE] inhibitors or angiotensin receptor blocker [ARB] therapy

© 2025 Cusick MM et al. JAMA Network Open.

eFigure 8. Univariate Sensitivity Analysis for Every 5 Years Screening From Ages 35 to 75 Years With SGLT2i for a) Hispanic Adults, b) Non-Hispanic Black Adults, c) Non-Hispanic White Adults, d) Adults From Additional Racial and Ethnic Groups, and e) the Overall Population Aged 35 Years

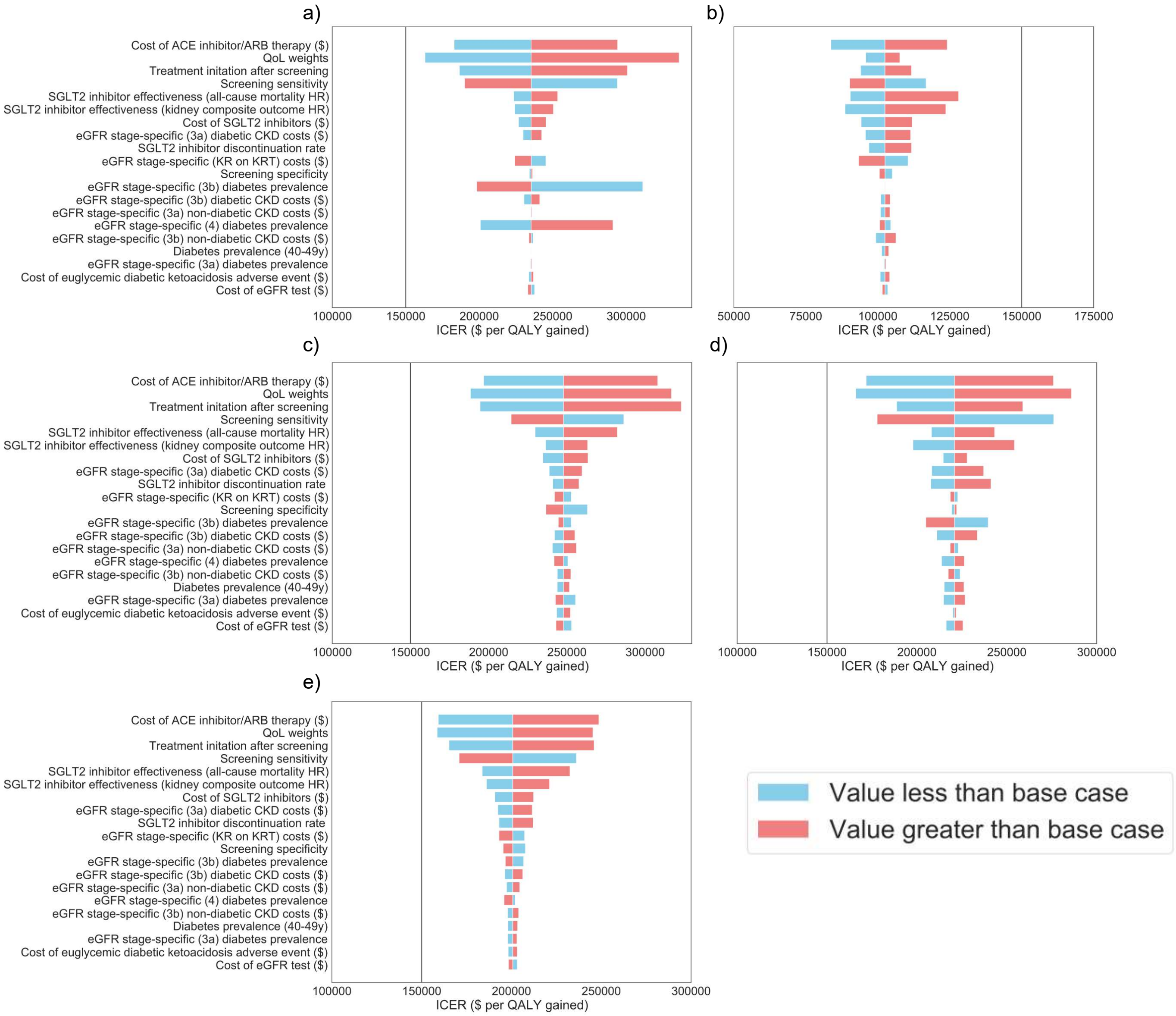

© 2025 Cusick MM et al. JAMA Network Open.

q5y: every 5 years; With SGLT2i: With the addition of sodium–glucose cotransporter-2 (SGLT2) inhibitors to conventional CKD therapy (angiotensin-converting enzyme [ACE] inhibitors or angiotensin receptor blocker [ARB] therapy

© 2025 Cusick MM et al. JAMA Network Open.

eReferences

1. Cusick MM, Tisdale RL, Chertow GM, Owens DK, Goldhaber-Fiebert JD. Population-Wide Screening for Chronic Kidney Disease: A Cost-Effectiveness Analysis. *Annals of Internal Medicine*. Published online May 23, 2023.

2. U.S. Census Bureau, Population Division. Annual Estimates of the Resident Population by Sex, Age, Race, and Hispanic Origin for the United States: April 1, 2010 to July 1, 2019 (NC-EST2019-ASR6H).

3. Goldhaber-Fiebert JD, Jalal HJ. Some Health States Are Better Than Others: Using Health State Rank Order to Improve Probabilistic Analyses. *Med Decis Making*. 2016;36(8):927-940. doi:10.1177/0272989X15605091

4. Turin TC, Tonelli M, Manns BJ, et al. Lifetime Risk of ESRD. *JASN*. 2012;23(9):1569-1578. doi:10.1681/ASN.2012020164

5. Wu HY, Peng YS, Chiang CK, et al. Diagnostic Performance of Random Urine Samples Using Albumin Concentration vs Ratio of Albumin to Creatinine for Microalbuminuria Screening in Patients With Diabetes Mellitus: A Systematic Review and Meta-analysis. *JAMA Internal Medicine*. 2014;174(7):1108-1115. doi:10.1001/jamainternmed.2014.1363

6. Creatinine, 24-Hour Urine. Find Lab Tests Online. Accessed June 27, 2022. https://www.findlabtest.com/lab-test/kidney-function-test/creatinine-24-hour-urine-quest-381

7. Sanders GD, Neumann PJ, Basu A, et al. Recommendations for Conduct, Methodological Practices, and Reporting of Cost-effectiveness Analyses: Second Panel on Cost-Effectiveness in Health and Medicine. *JAMA*. 2016;316(10):1093. doi:10.1001/jama.2016.12195

8. Golan L, Birkmeyer JD, Welch HG. The Cost-Effectiveness of Treating All Patients with Type 2 Diabetes with Angiotensin-Converting Enzyme Inhibitors. *Ann Intern Med*. 1999;131(9):660-667. doi:10.7326/0003-4819-131-9-199911020-00005

9. Boulware LE, Jaar BG, Tarver-Carr ME, Brancati FL, Powe NR. Screening for Proteinuria in US AdultsA Cost-effectiveness Analysis. *JAMA*. 2003;290(23):3101-3114. doi:10.1001/jama.290.23.3101

10. Serum Creatinine Test Cost. Find Lab Tests Online. Accessed June 27, 2022. https://www.findlabtest.com/lab-test/kidney-function-test/serum-creatinine-test-cost-quest-375

11. Abdominal Ultrasound Cost and Procedure Comparison | NCH. NewChoiceHealth.com. Accessed June 28, 2022. https://www.newchoicehealth.com/procedures/abdominal-ultrasound

12. Ruggenenti P, Perna A, Gherardi G, et al. Renoprotective properties of ACE-inhibition in non-diabetic nephropathies with non-nephrotic proteinuria. *The Lancet*. 1999;354(9176):359-364. doi:10.1016/S0140-6736(98)10363-X

13. Brenner BM, Cooper ME, de Zeeuw D, et al. Effects of Losartan on Renal and Cardiovascular Outcomes in Patients with Type 2 Diabetes and Nephropathy. *N Engl J Med*. 2001;345(12):861-869. doi:10.1056/NEJMoa011161

14. Hou FF, Zhang X, Zhang GH, et al. Efficacy and Safety of Benazepril for Advanced Chronic Renal Insufficiency. *New England Journal of Medicine*. 2006;354(2):131-140. doi:10.1056/NEJMoa053107

15. Hou FF, Xie D, Zhang X, et al. Renoprotection of Optimal Antiproteinuric Doses (ROAD) Study: A Randomized Controlled Study of Benazepril and Losartan in Chronic Renal Insufficiency. *JASN*. 2007;18(6):1889-1898. doi:10.1681/ASN.2006121372

© 2025 Cusick MM et al. JAMA Network Open.

16. Medicare Part D Spending by Drug | CMS Data. Accessed October 23, 2024. https://data.cms.gov/summary-statistics-on-use-and-payments/medicare-medicaid-spending-by-drug/medicare-part-d-spending-by-drug

17. Heerspink HJL, Stefánsson BV, Correa-Rotter R, et al. Dapagliflozin in Patients with Chronic Kidney Disease. *N Engl J Med*. Published online September 24, 2020:NEJMoa2024816. doi:10.1056/NEJMoa2024816

18. Medicare Drug Price Negotiation Program: Negotiated Prices for Initial Price Applicability Year 2026 | CMS. Accessed October 23, 2024. https://www.cms.gov/newsroom/fact-sheets/medicare-drug-price-negotiation-program-negotiated-prices-initial-price-applicability-year-2026

19. Richman IB, Fairley M, Jørgensen ME, Schuler A, Owens DK, Goldhaber-Fiebert JD. Cost-effectiveness of Intensive Blood Pressure Management. *JAMA Cardiology*. 2016;1(8):872-879. doi:10.1001/jamacardio.2016.3517

20. Files for FY 2010 Final Rule and Correction Notice | CMS. Accessed June 27, 2022. https://www.cms.gov/Medicare/Medicare-Fee-for-Service-Payment/AcuteInpatientPPS/Acute-Inpatient-Files-for-Download-Items/CMS1247873

21. Vleeming W, van Amsterdam JG, Stricker BH, de Wildt DJ. ACE inhibitor-induced angioedema. Incidence, prevention and management. *Drug Saf*. 1998;18(3):171-188. doi:10.2165/00002018-199818030-00003

22. Sullivan PW, Ghushchyan VH. EQ-5D Scores for Diabetes-Related Comorbidities. *Value in Health*. 2016;19(8):1002-1008. doi:10.1016/j.jval.2016.05.018

23. Cost-Effectiveness Analysis of Canagliflozin 300 mg Versus Dapagliflozin 10 mg Added to Metformin in Patients with Type 2 Diabetes in the United States - PMC. Accessed January 20, 2023. https://www-ncbi-nlm-nih-gov.stanford.idm.oclc.org/pmc/articles/PMC6104269/

24. Clar C, Gill JA, Court R, Waugh N. Systematic review of SGLT2 receptor inhibitors in dual or triple therapy in type 2 diabetes. *BMJ Open*. 2012;2(5):e001007. doi:10.1136/bmjopen-2012-001007

25. Peasgood T, Brennan A, Mansell P, Elliott J, Basarir H, Kruger J. The Impact of Diabetes-Related Complications on Preference-Based Measures of Health-Related Quality of Life in Adults with Type I Diabetes. *Med Decis Making*. 2016;36(8):1020-1033. doi:10.1177/0272989X16658660

26. Recurrent DKA results in high societal costs – a retrospective study identifying social predictors of recurrence for potential future intervention | Clinical Diabetes and Endocrinology | Full Text. Accessed January 20, 2023. https://clindiabetesendo.biomedcentral.com/articles/10.1186/s40842-021-00127-6

27. Sodium–Glucose Cotransporter-2 Inhibitors and the Risk for Diabetic Ketoacidosis: A Multicenter Cohort Study: Annals of Internal Medicine: Vol 173, No 6. Accessed January 20, 2023. https://www-acpjournals-org.laneproxy.stanford.edu/doi/10.7326/M20-0289?url_ver=Z39.88-2003&rfr_id=ori:rid:crossref.org&rfr_dat=cr_pub%20%200pubmed

28. Centers for Disease Control and Prevention (CDC). National Center for Health Statistics (NCHS). *National Health and Nutrition Examination Survey Data*. U.S. Department of Health and Human Services, Centers for Disease Control and Prevention; 2019. https://www.cdc.gov/nchs/nhanes/index.htm

29. Go AS, Chertow GM, Fan D, McCulloch CE, Hsu C yuan. Chronic Kidney Disease and the Risks of Death, Cardiovascular Events, and Hospitalization. *N Engl J Med*. 2004;351(13):1296-1305. doi:10.1056/NEJMoa041031

30. Cooper JT, Lloyd A, Sanchez JJG, Sörstadius E, Briggs A, McFarlane P. Health related quality of life utility weights for economic evaluation through different stages of chronic kidney disease: a systematic literature review. *Health Qual Life Outcomes*. 2020;18(1):310. doi:10.1186/s12955-020-01559-x

© 2025 Cusick MM et al. JAMA Network Open.

31. Nichols GA, Ustyugova A, Déruaz-Luyet A, O'Keeffe-Rosetti M, Brodovicz KG. Health Care Costs by Type of Expenditure across eGFR Stages among Patients with and without Diabetes, Cardiovascular Disease, and Heart Failure. *Journal of the American Society of Nephrology*. 2020;31(7):1594. doi:10.1681/ASN.2019121308

32. Agency for Healthcare Research and Quality. Medical Expenditure Panel Survey (MEPS). Published online July 2024. https://www.ahrq.gov/data/meps.html

© 2025 Cusick MM et al. JAMA Network Open.
