# Supplementary material for: Balancing Efficiency and Equity in Population-Wide CKD Screening
Source: JAMA Netw Open. 2025 Apr 14;8(4):e254740. doi: 10.1001/jamanetworkopen.2025.4740 (PMC11997725; doi:10.1001/jamanetworkopen.2025.4740)
Supplement: Supplement 2. — Data Sharing Statement [file jamanetwopen-e254740-s002.pdf]

Data Sharing Statement

Cusick. Balancing Efficiency and Equity in Population-Wide CKD Screening. *JAMA Netw Open*. Published April 14, 2025. doi:10.1001/jamanetworkopen.2025.4740

Data
Data available: No

Additional Information
Explanation for why data not available: No individual patient data were used in this study. Our model was informed by data that are available from published literature and publicly available data sources cited in our manuscript. Our code is available: https://github.com/marikamaecusick/CKDScreeningCEA
